# Supplementary figures and images for: Novel axonemal protein ZMYND12 interacts with TTC29 and DNAH1, and is required for male fertility and flagellum function
Source: eLife. 2023 Nov 7;12:RP87698. doi: 10.7554/eLife.87698 (PMC10629824; doi:10.7554/eLife.87698)

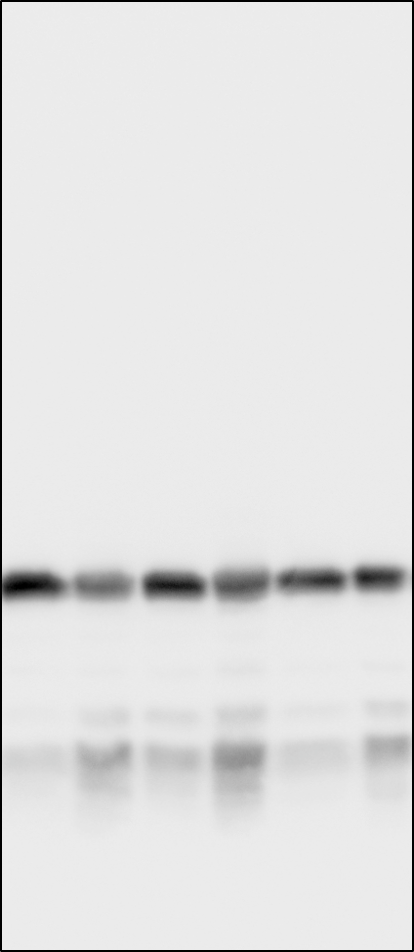

Supplement: Figure 3—source data 1. [file elife-87698-fig3-data1.zip › Figure 3-source data 1/Figure 3B-antiSAXO.tif]

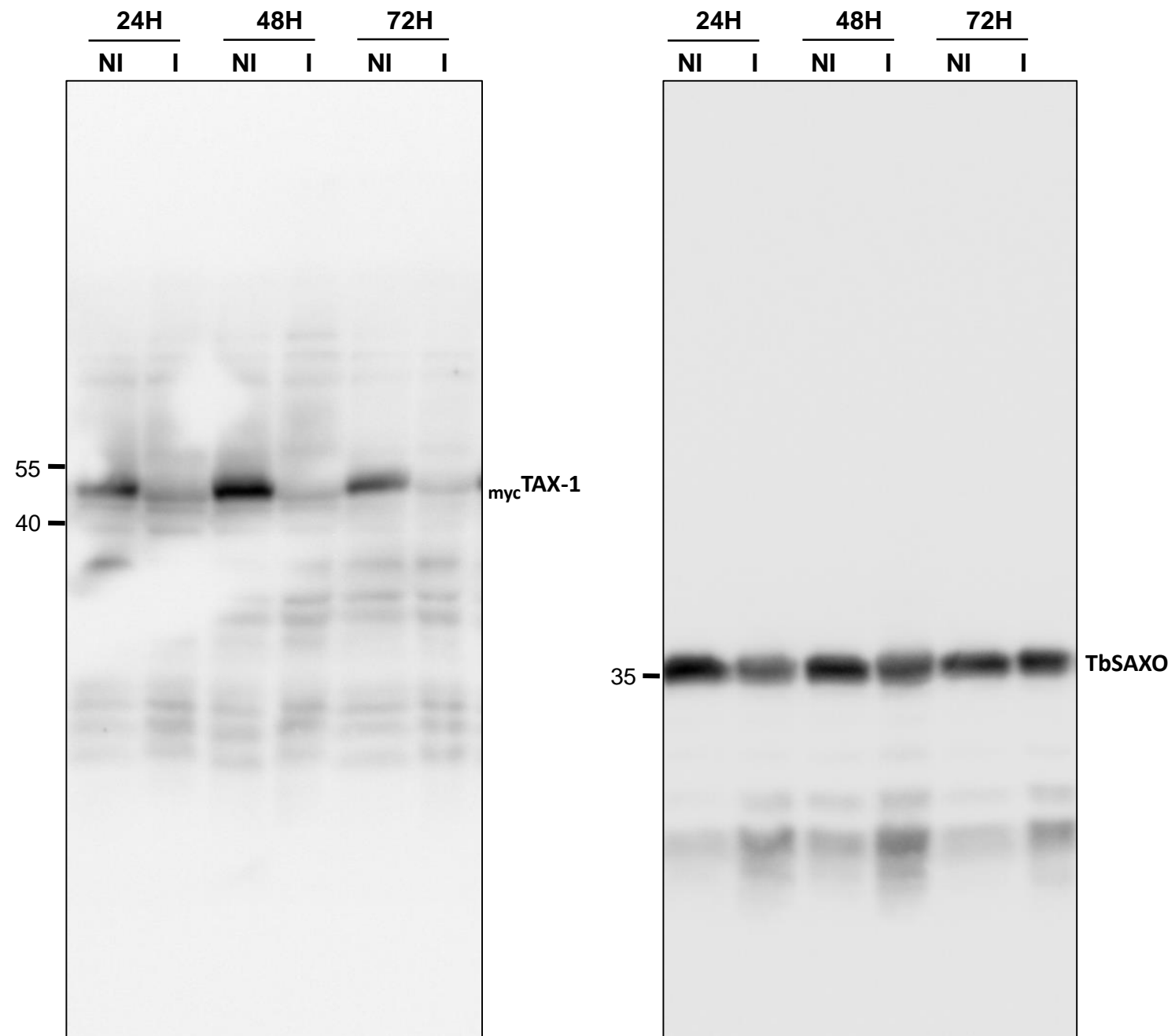

Supplement: Figure 3—source data 1. [file elife-87698-fig3-data1.zip › Figure 3-source data 1/Figure 3B-uncropped blot.pdf]

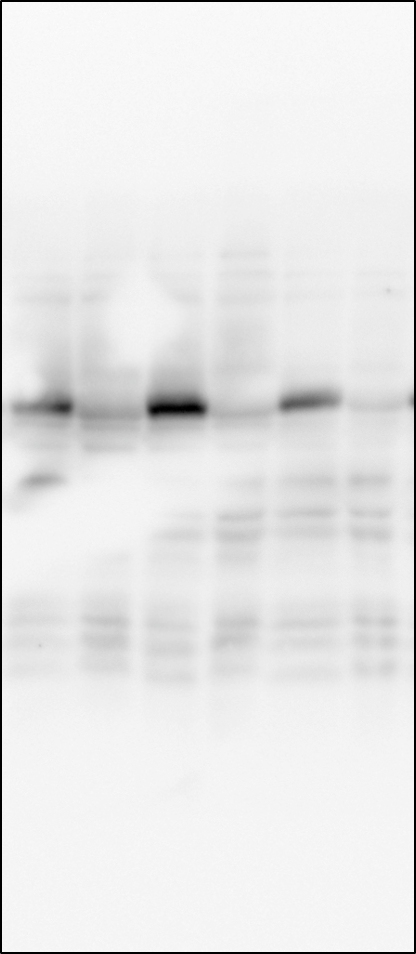

Supplement: Figure 3—source data 1. [file elife-87698-fig3-data1.zip › Figure 3-source data 1/Figure 3B-antiTAX-1myc.tif]

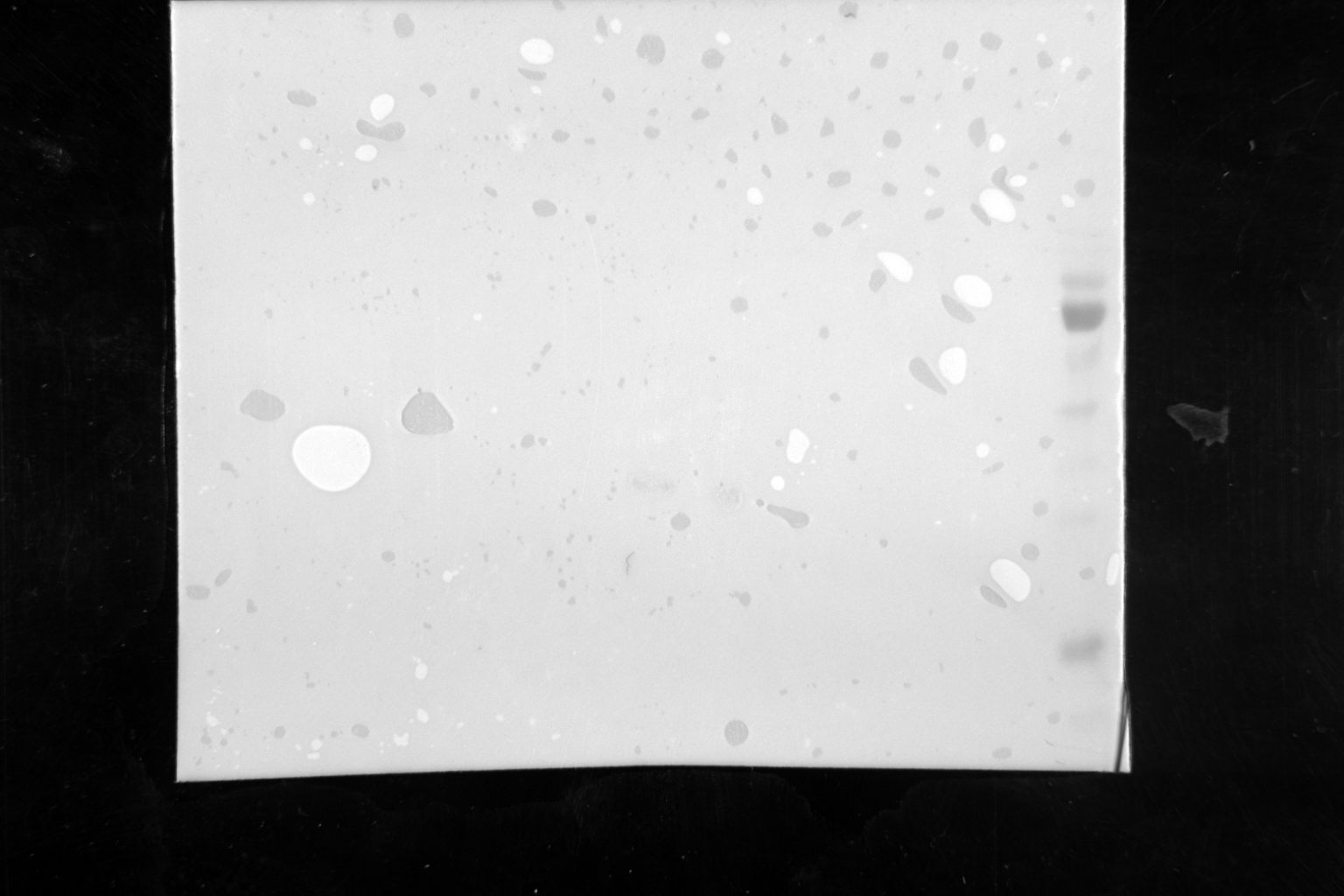

Supplement: Figure 3—source data 1. [file elife-87698-fig3-data1.zip › Figure 3-source data 1/Raw images/MW V.tif]

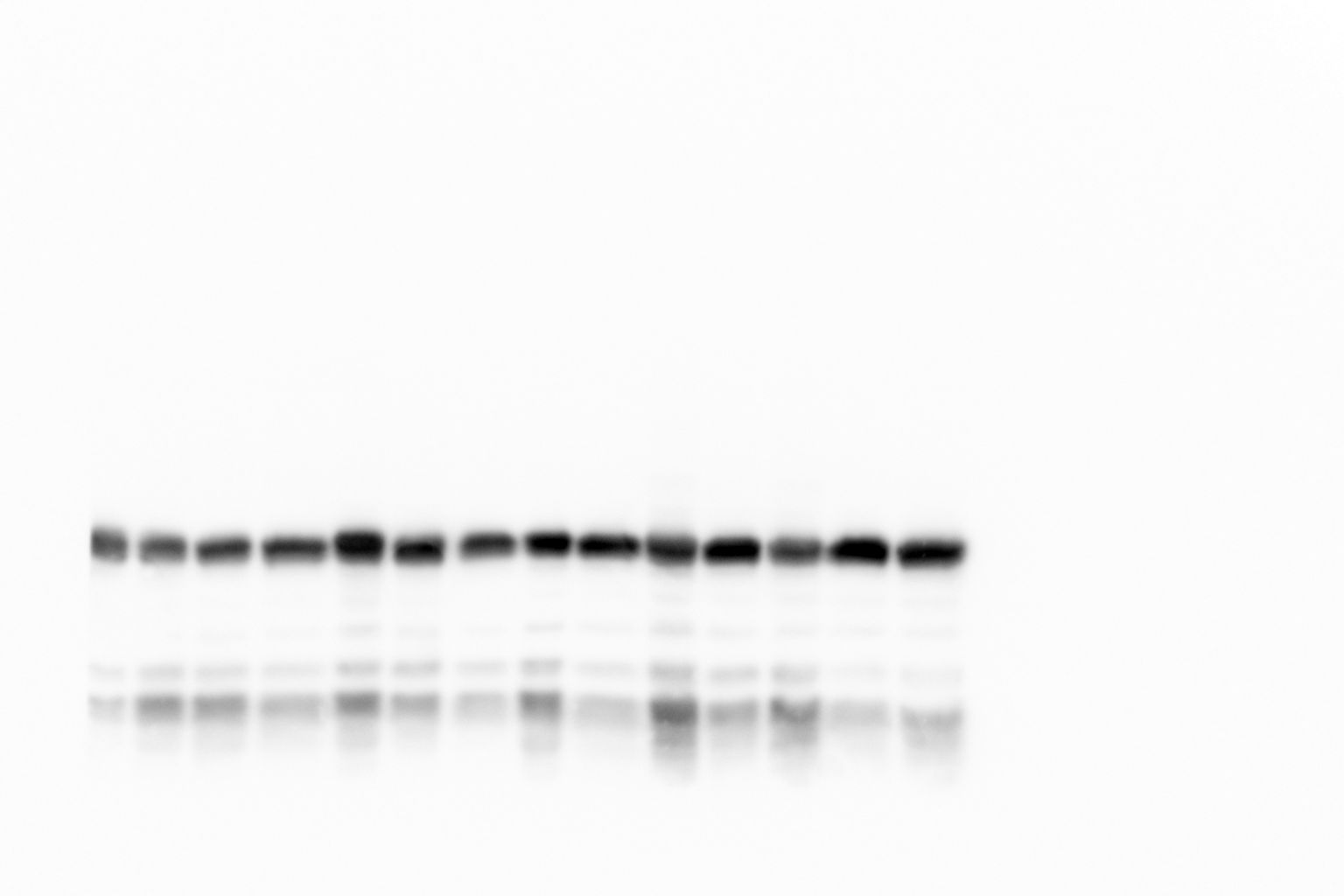

Supplement: Figure 3—source data 1. [file elife-87698-fig3-data1.zip › Figure 3-source data 1/Raw images/Figure 3B-anti Saxo.tif]

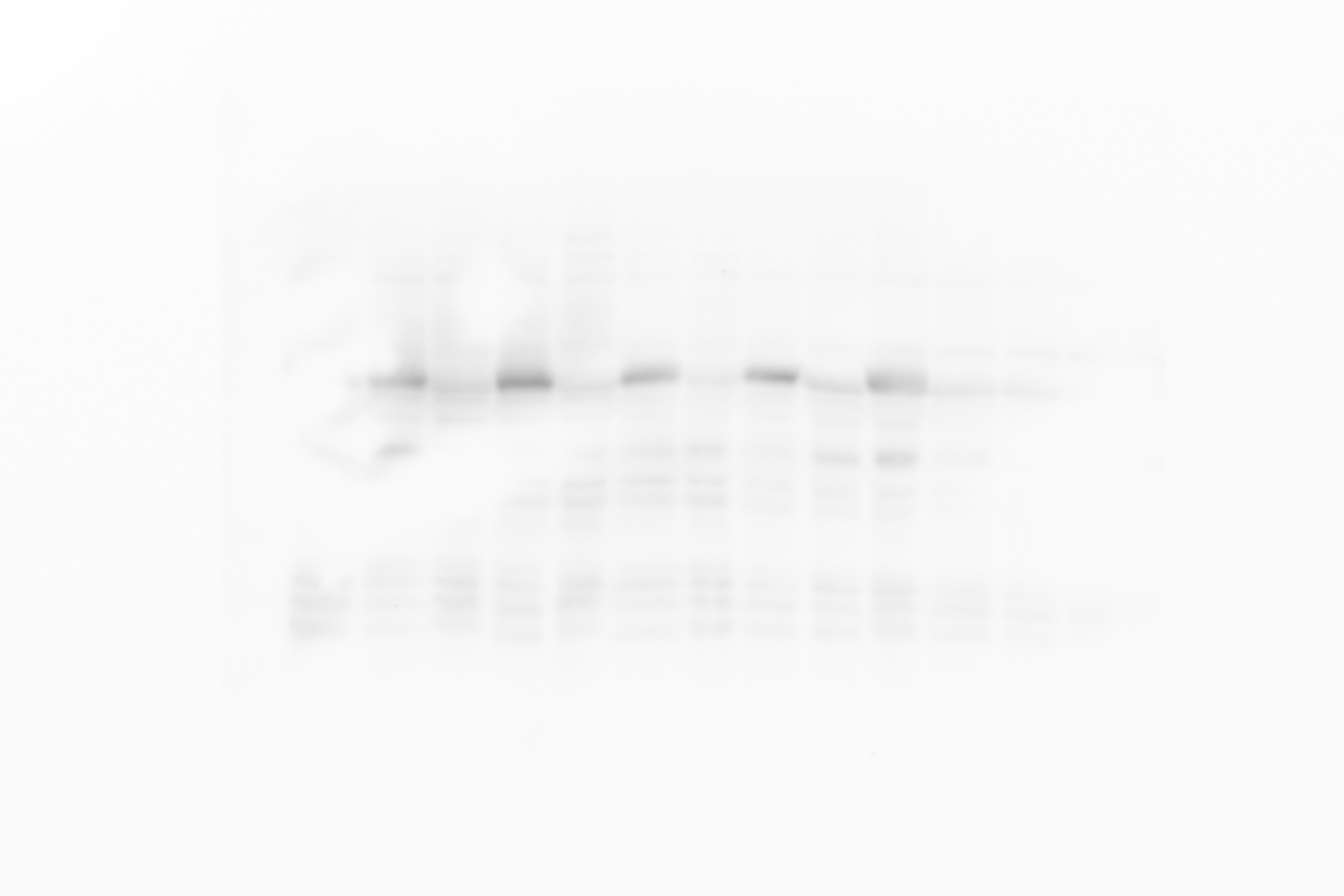

Supplement: Figure 3—source data 1. [file elife-87698-fig3-data1.zip › Figure 3-source data 1/Raw images/Figure 3B-antiTAX-1myc.tif]

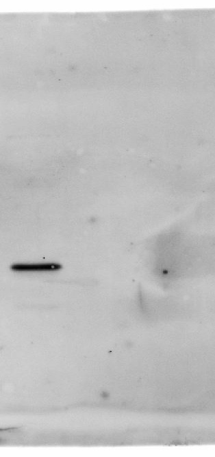

Supplement: Figure 4—source data 1. [file elife-87698-fig4-data1.zip › Figure 4-source data 1/Figure 4B-antiTAX1TruncTy1.tif]

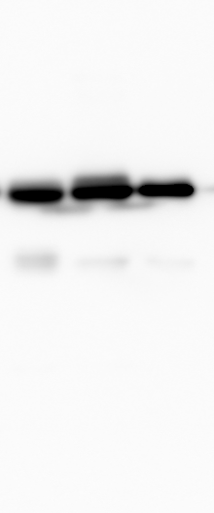

Supplement: Figure 4—source data 1. [file elife-87698-fig4-data1.zip › Figure 4-source data 1/Figure 4B-antiTubulin.tif]

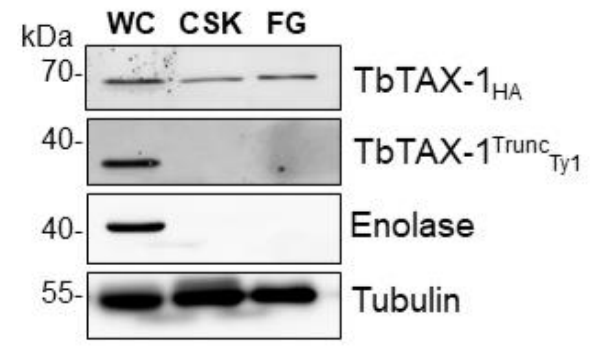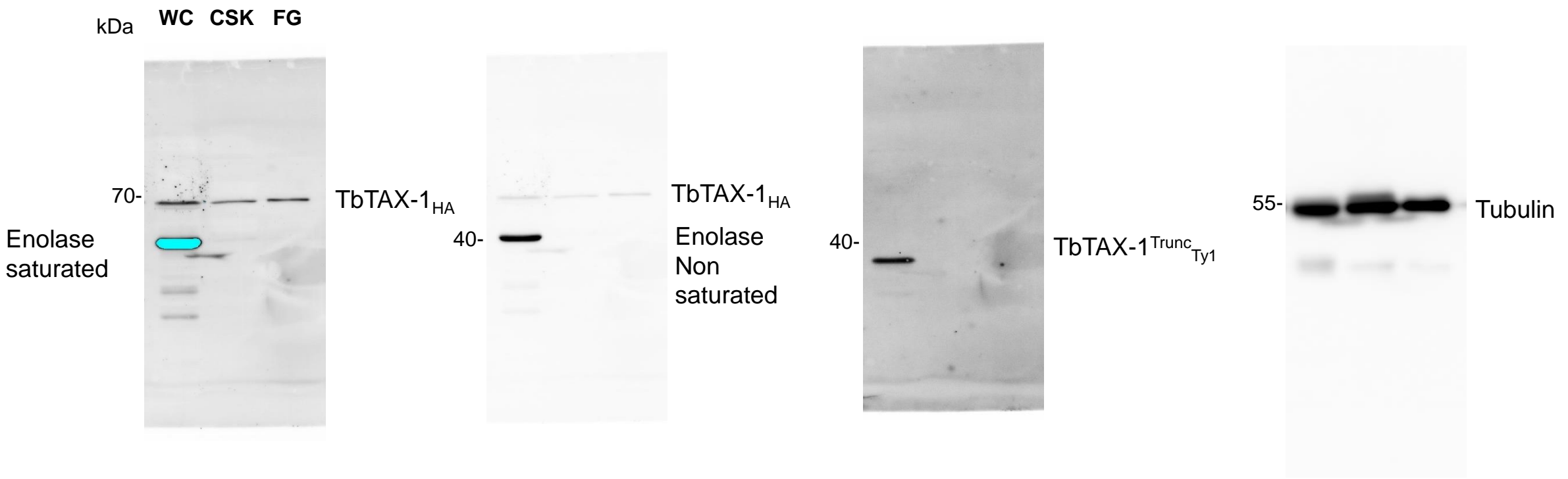

Supplement: Figure 4—source data 1. [file elife-87698-fig4-data1.zip › Figure 4-source data 1/Figure 4B-uncropped gel.pdf]

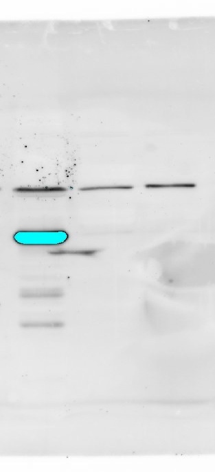

Supplement: Figure 4—source data 1. [file elife-87698-fig4-data1.zip › Figure 4-source data 1/Figure 4B-antiTAX1HA_enolase satured.tif]

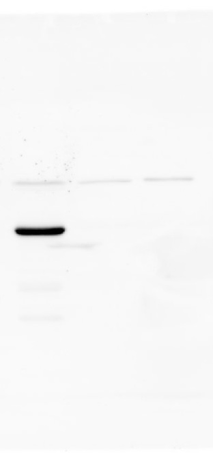

Supplement: Figure 4—source data 1. [file elife-87698-fig4-data1.zip › Figure 4-source data 1/Figure 4B-antiTAX1HA_enolase non satured.tif]

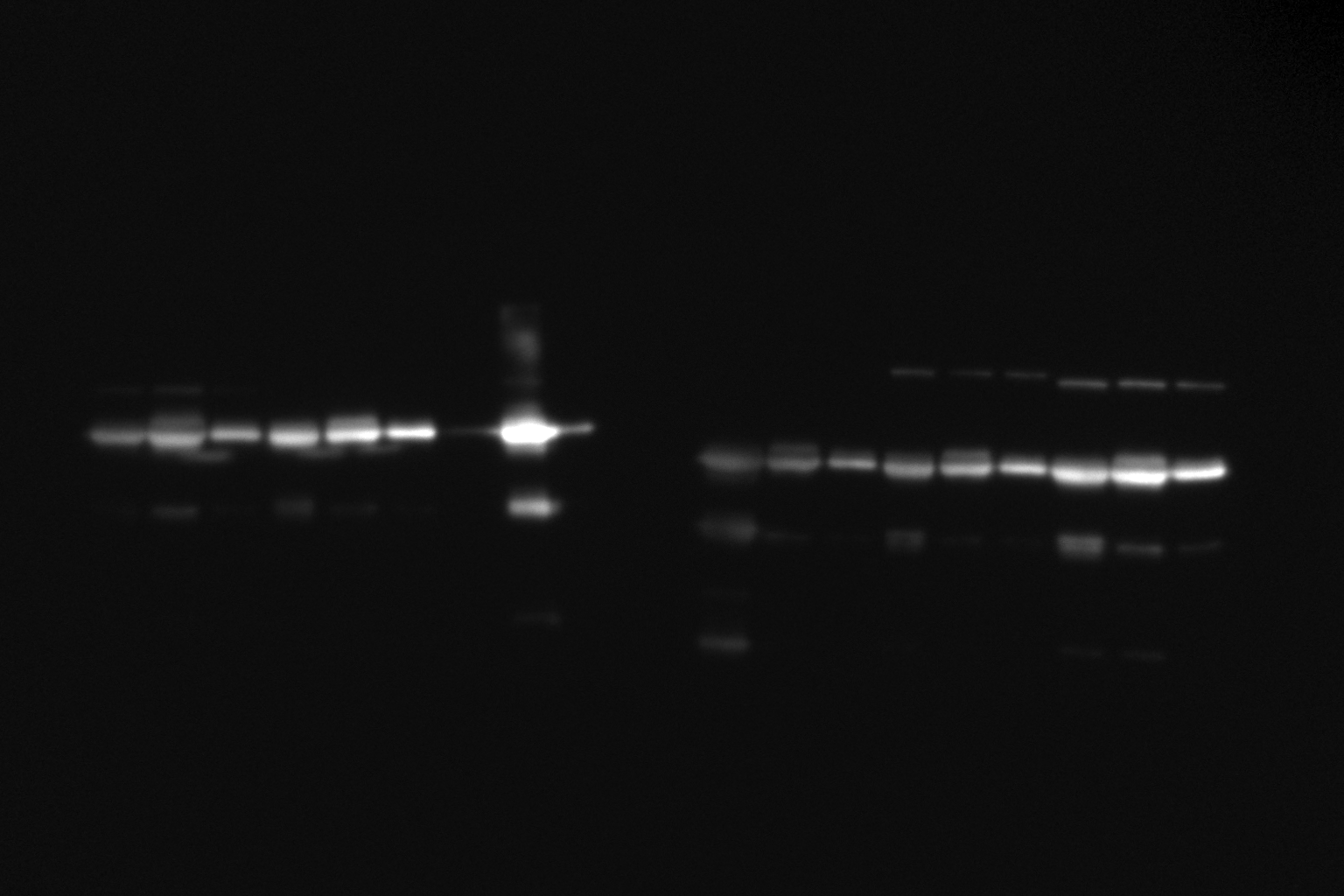

Supplement: Figure 4—source data 1. [file elife-87698-fig4-data1.zip › Figure 4-source data 1/Raw images/Figure 4B-Tubuline.tif]

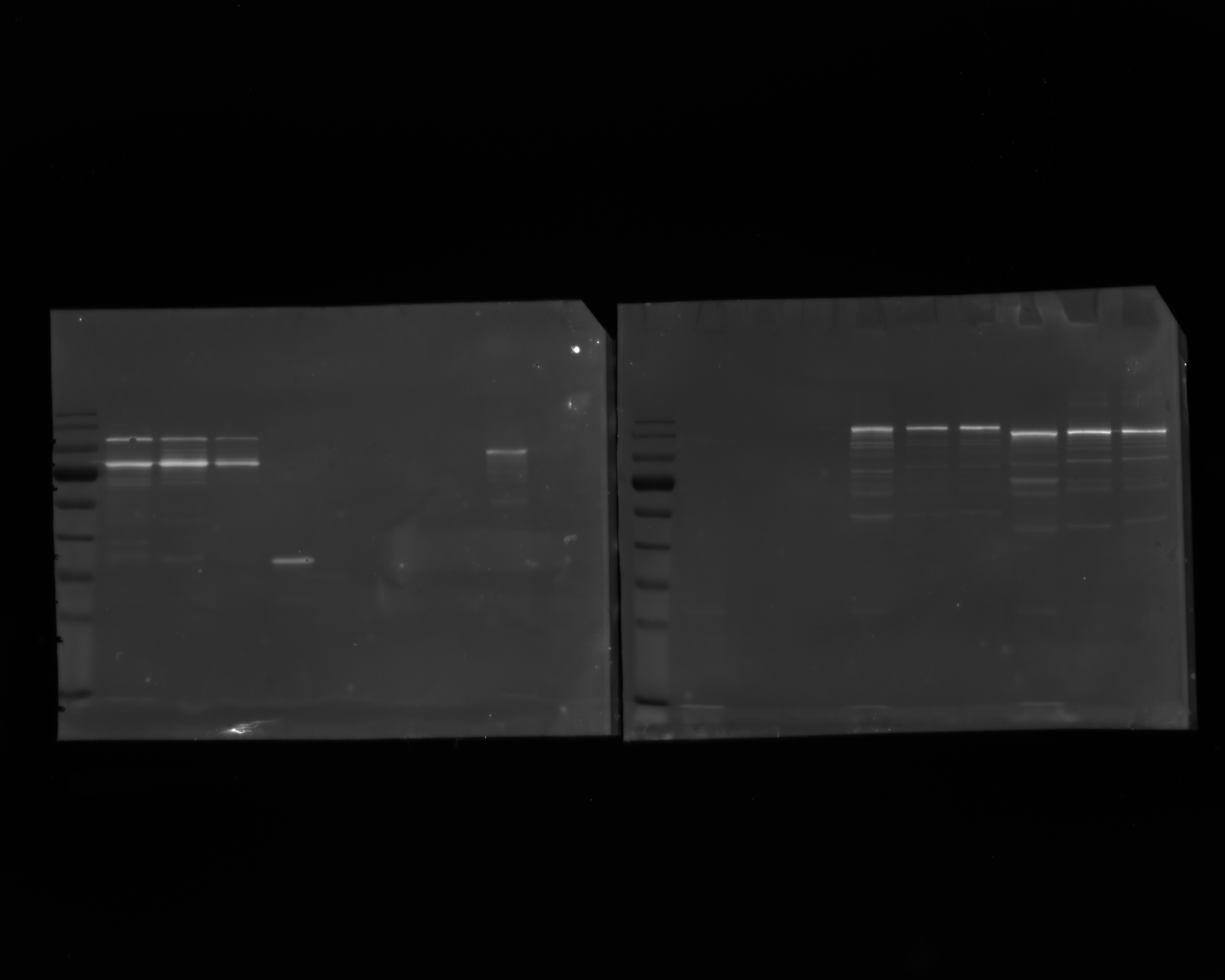

Supplement: Figure 4—source data 1. [file elife-87698-fig4-data1.zip › Figure 4-source data 1/Raw images/Figure 4B-TAX1-Trunc.tif]

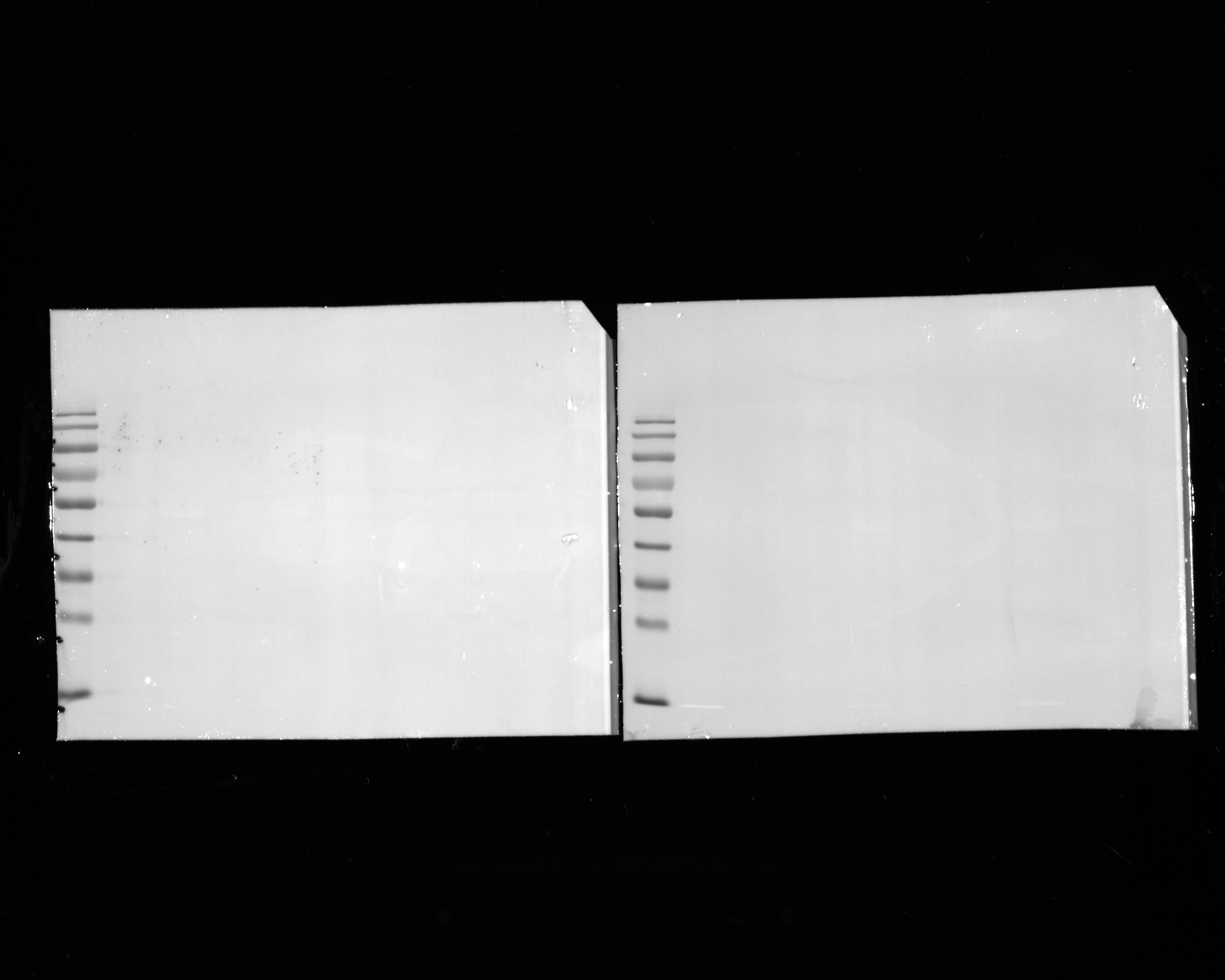

Supplement: Figure 4—source data 1. [file elife-87698-fig4-data1.zip › Figure 4-source data 1/Raw images/Figure 4B-MW.tif]

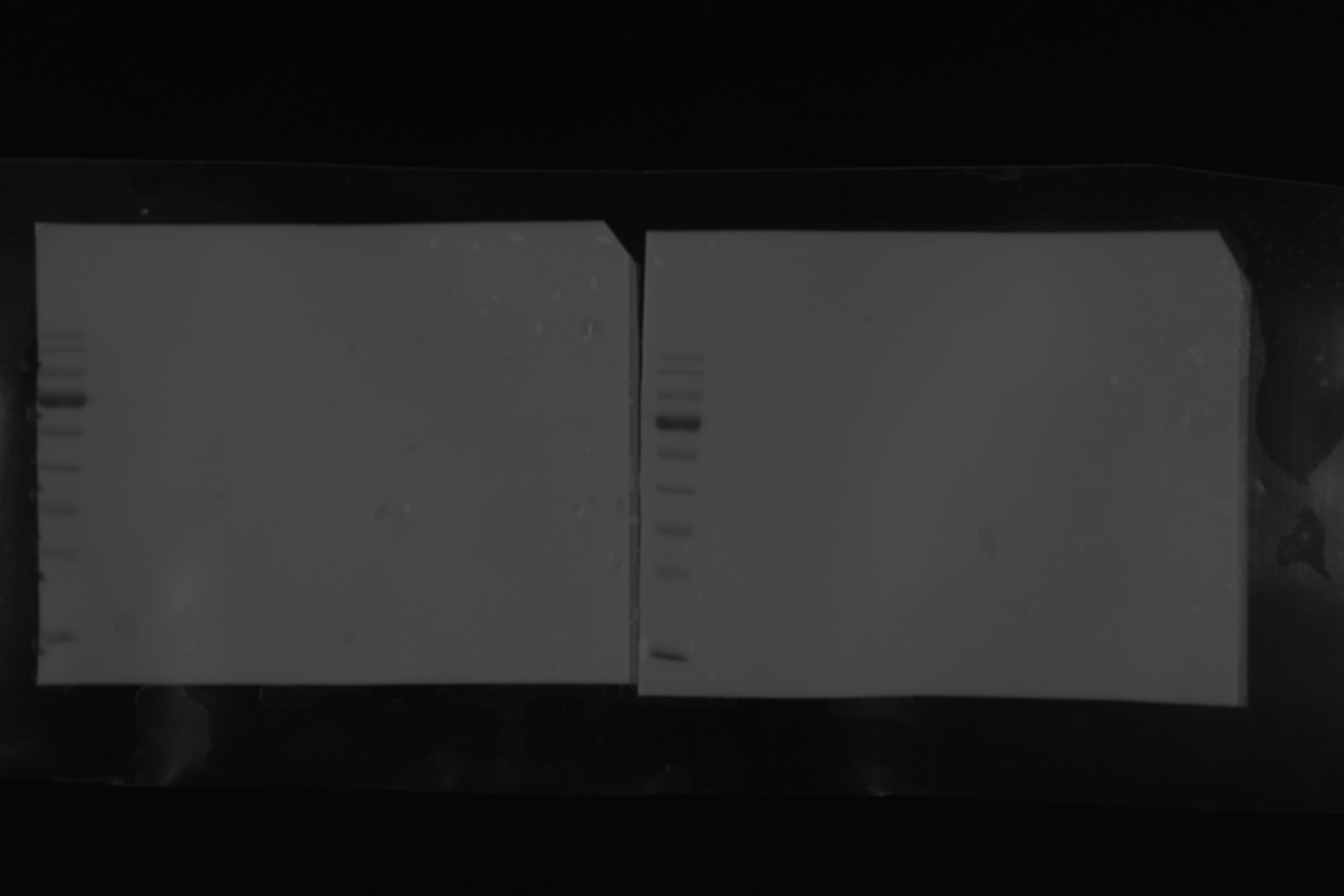

Supplement: Figure 4—source data 1. [file elife-87698-fig4-data1.zip › Figure 4-source data 1/Raw images/Figure 4B-MW Tubuline V.tif]

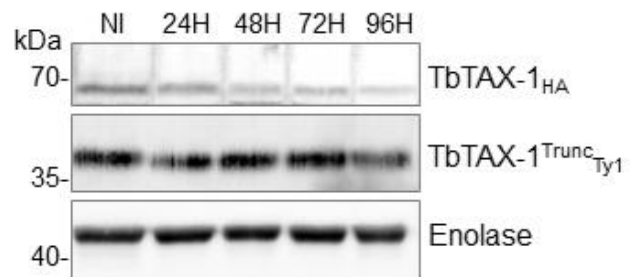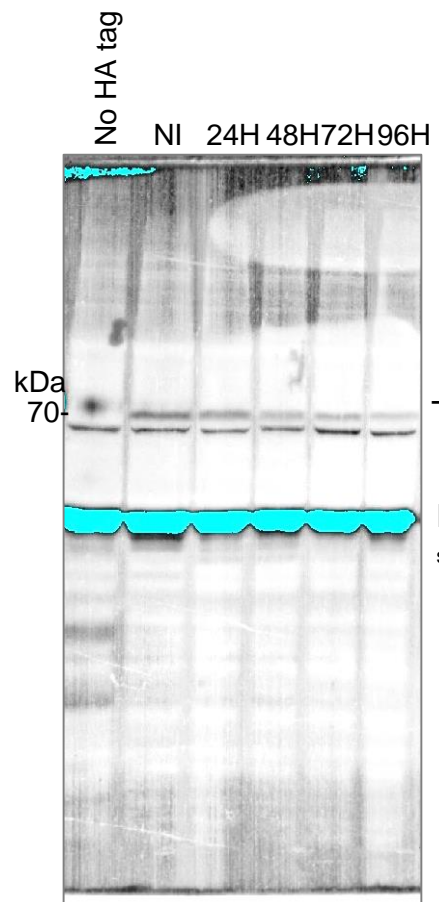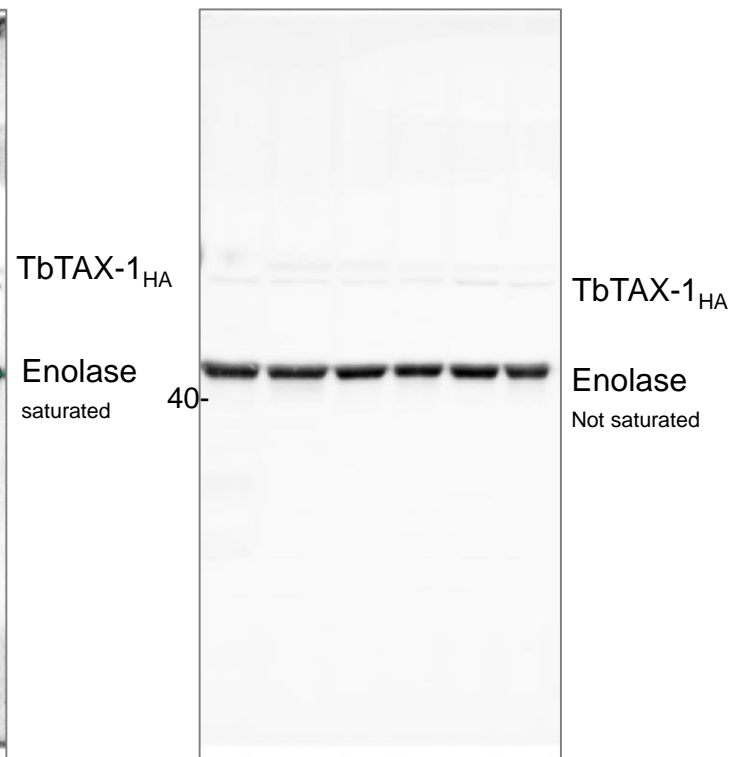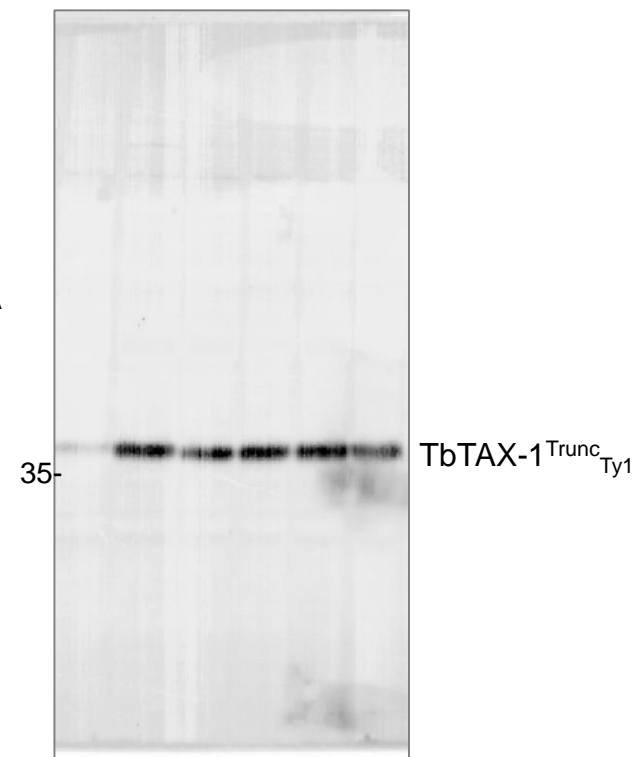

Supplement: Figure 4—source data 2. [file elife-87698-fig4-data2.zip › Figure 4-source data 2/Figure 4D-uncropped blots.pdf]

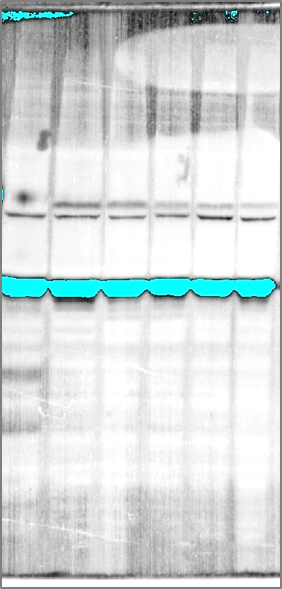

Supplement: Figure 4—source data 2. [file elife-87698-fig4-data2.zip › Figure 4-source data 2/Figure 4D-antiTAX1HA-enolase satured.tif]

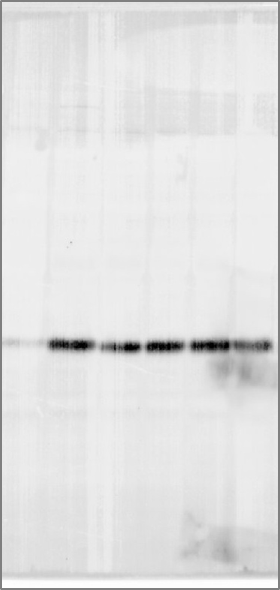

Supplement: Figure 4—source data 2. [file elife-87698-fig4-data2.zip › Figure 4-source data 2/Figure 4D-antiTAX1TruncTy1.tif]

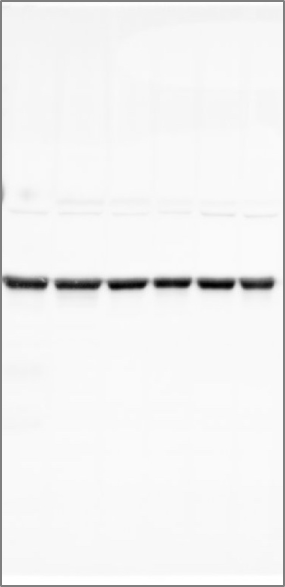

Supplement: Figure 4—source data 2. [file elife-87698-fig4-data2.zip › Figure 4-source data 2/Figure 4D-antiTAX1HA-enolase non satured.tif]

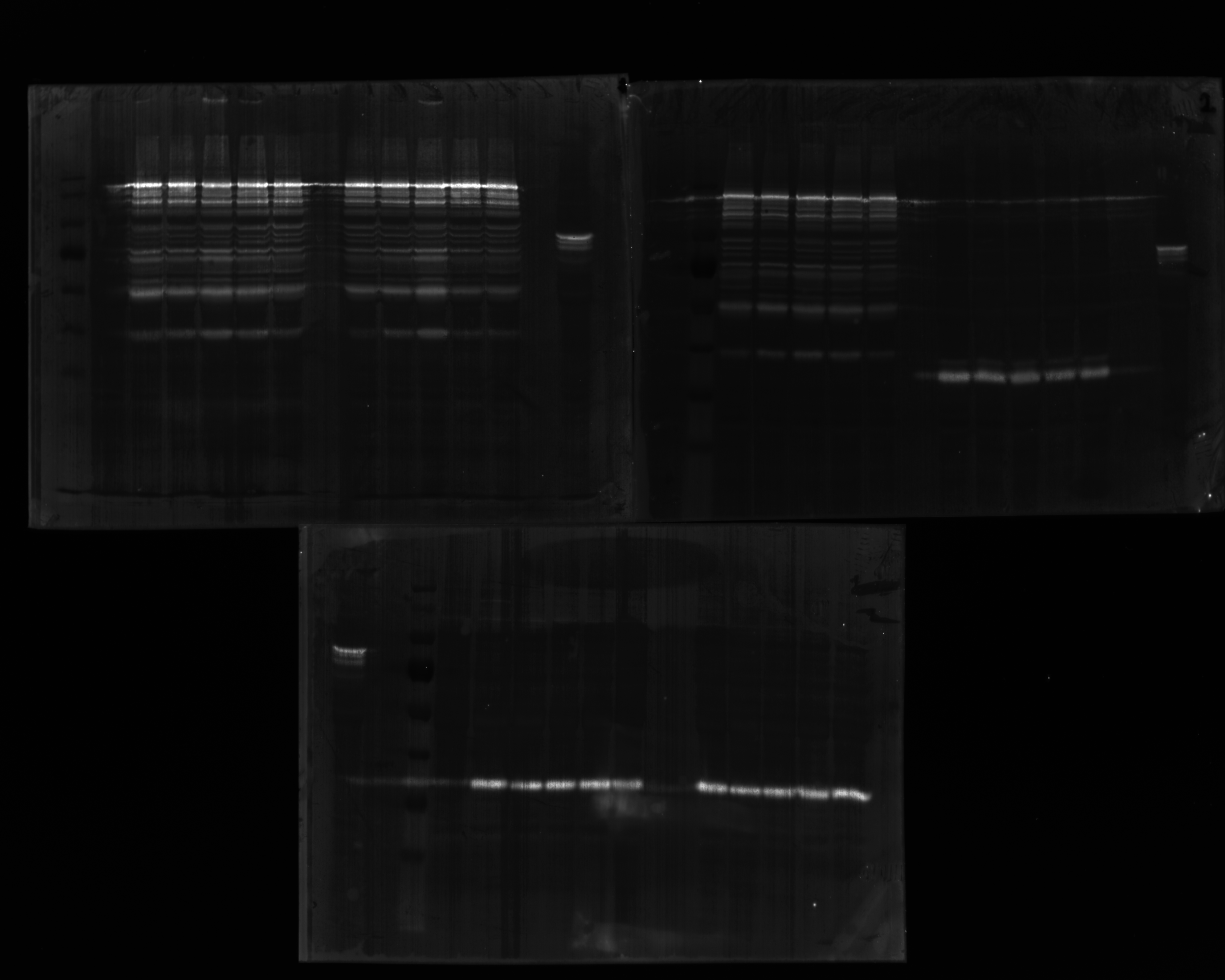

Supplement: Figure 4—source data 2. [file elife-87698-fig4-data2.zip › Figure 4-source data 2/Raw images/Figure 4D-TAX1-Trunc.tif]

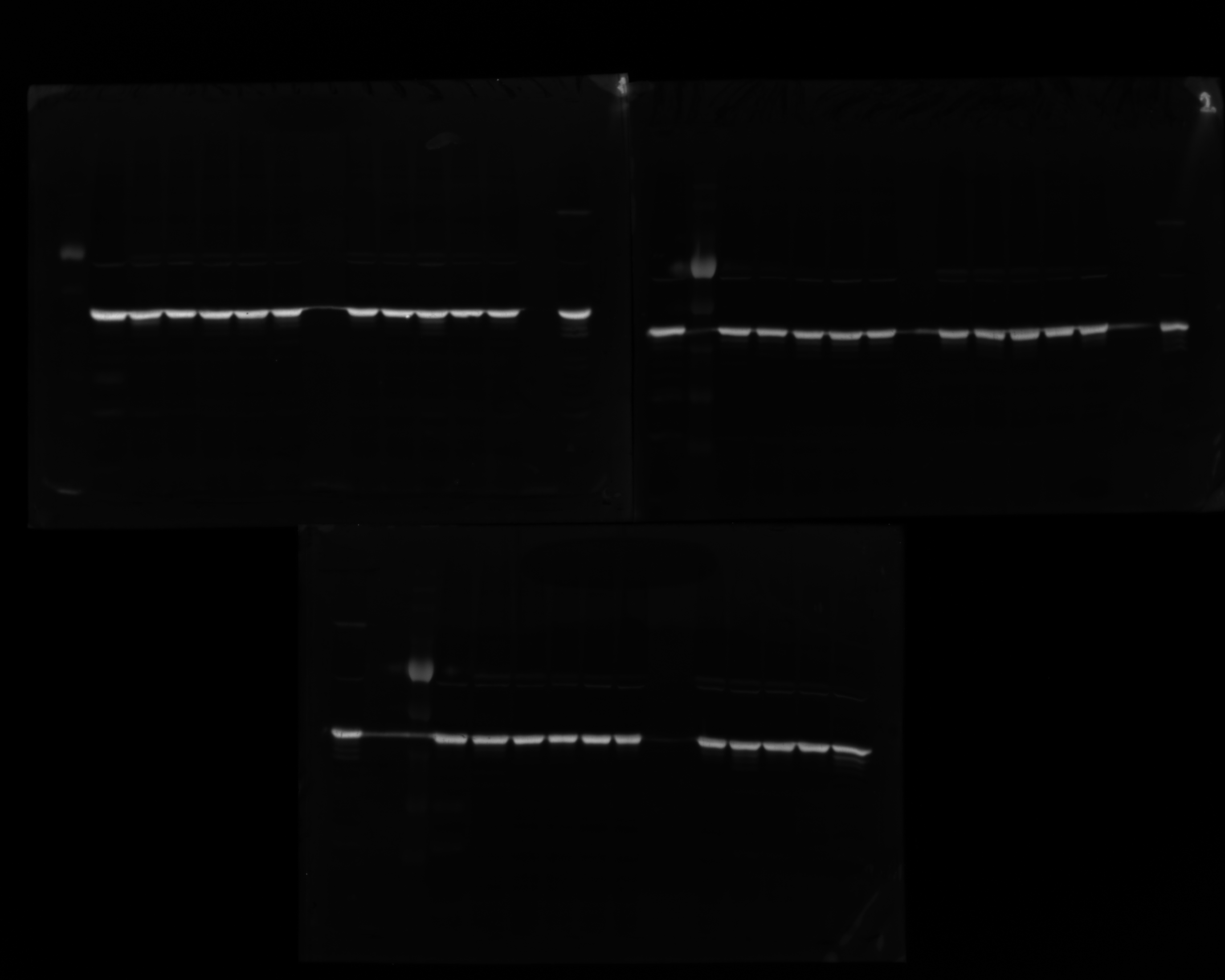

Supplement: Figure 4—source data 2. [file elife-87698-fig4-data2.zip › Figure 4-source data 2/Raw images/Figure 4D-Enolase.tif]

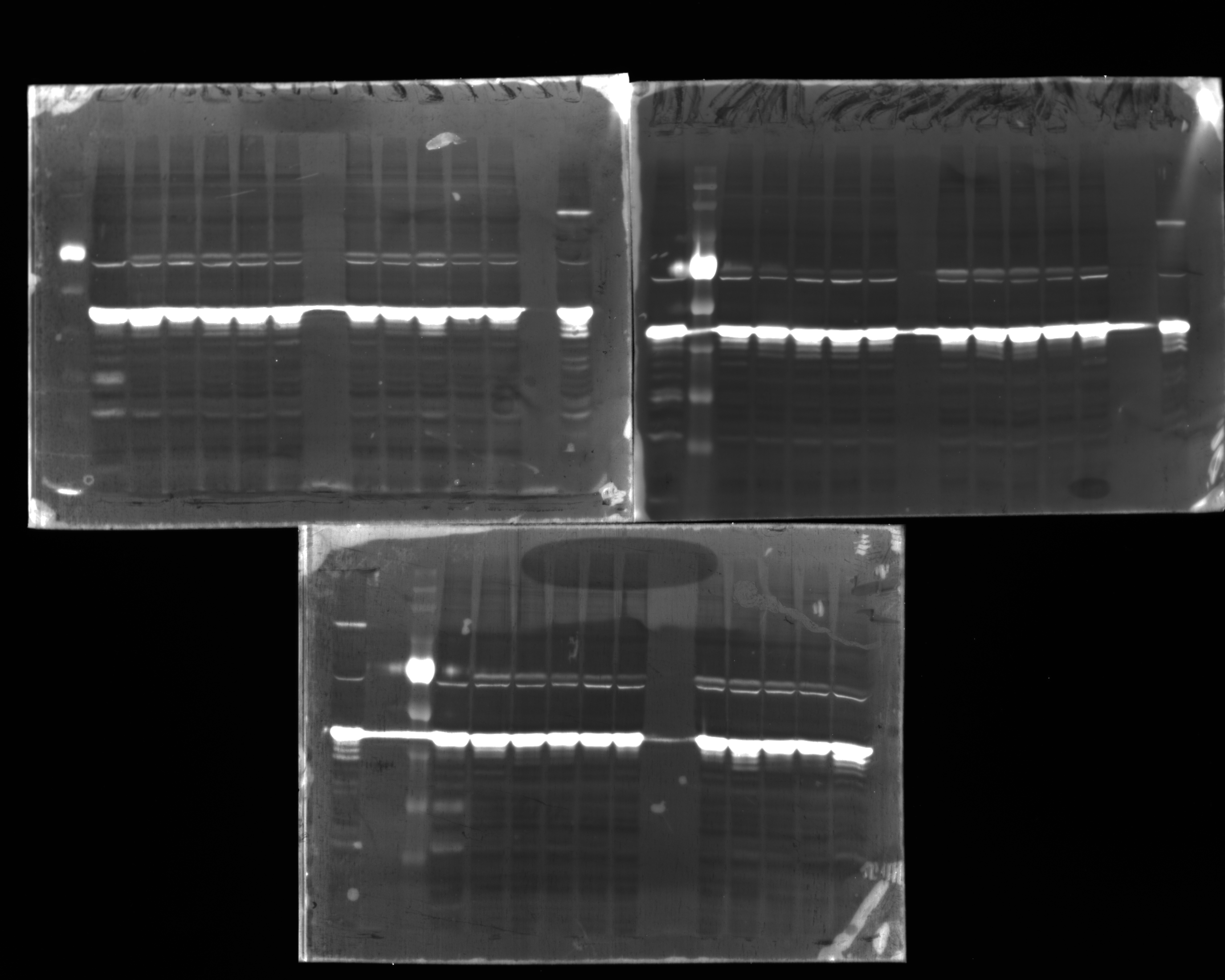

Supplement: Figure 4—source data 2. [file elife-87698-fig4-data2.zip › Figure 4-source data 2/Raw images/Figure 4D-TAX1.tif]

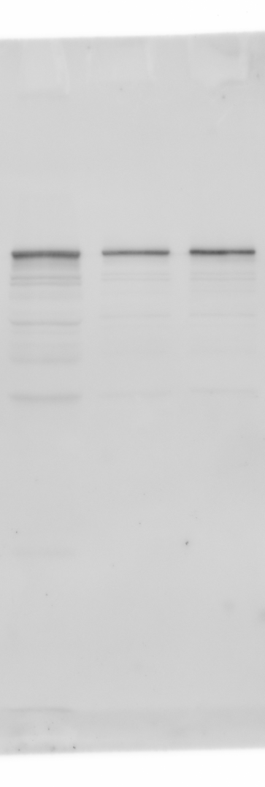

Supplement: Figure 4—figure supplement 1—source data 1. [file elife-87698-fig4-figsupp1-data1.zip › Figure 4-figure supplement 1-source data 1/Figure 4-supplement 1A-WDR66-antiTy1.tif]

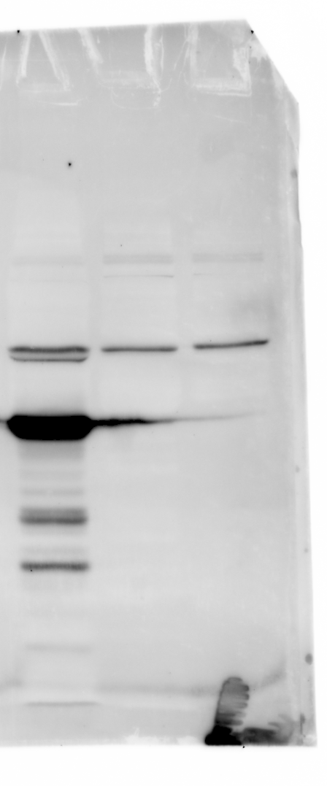

Supplement: Figure 4—figure supplement 1—source data 1. [file elife-87698-fig4-figsupp1-data1.zip › Figure 4-figure supplement 1-source data 1/Figure 4-supplement 1A-CFAP70-antiHA_antiEnolase saturated.tif]

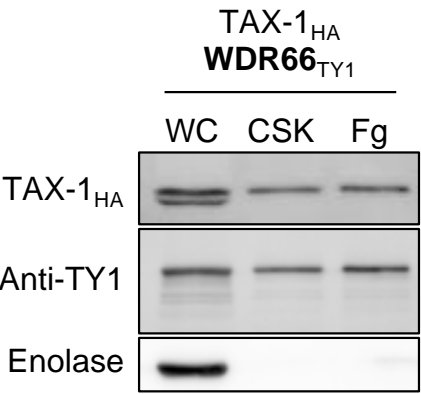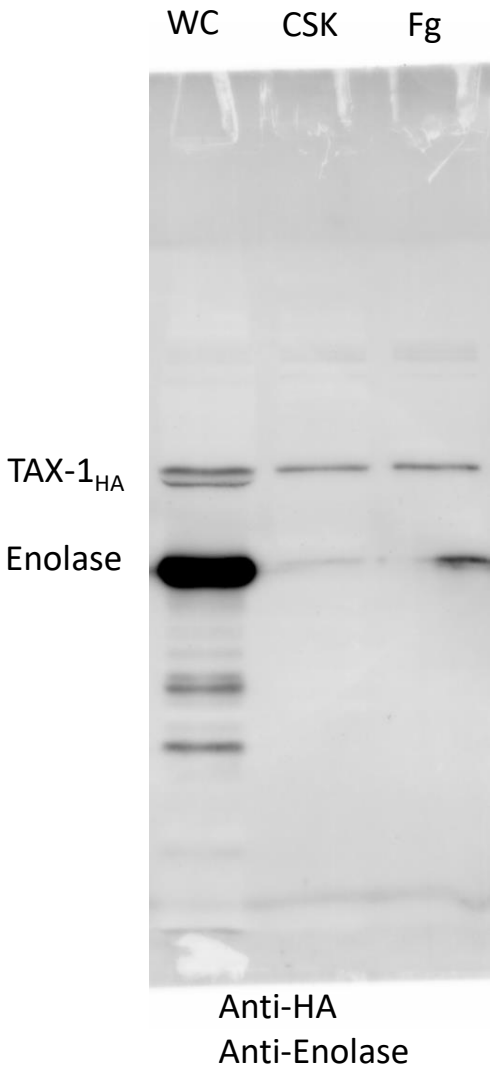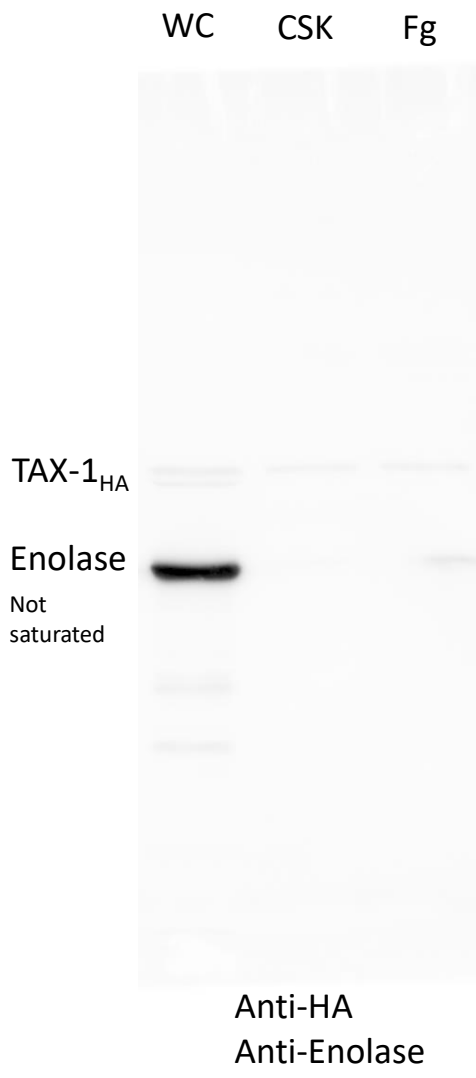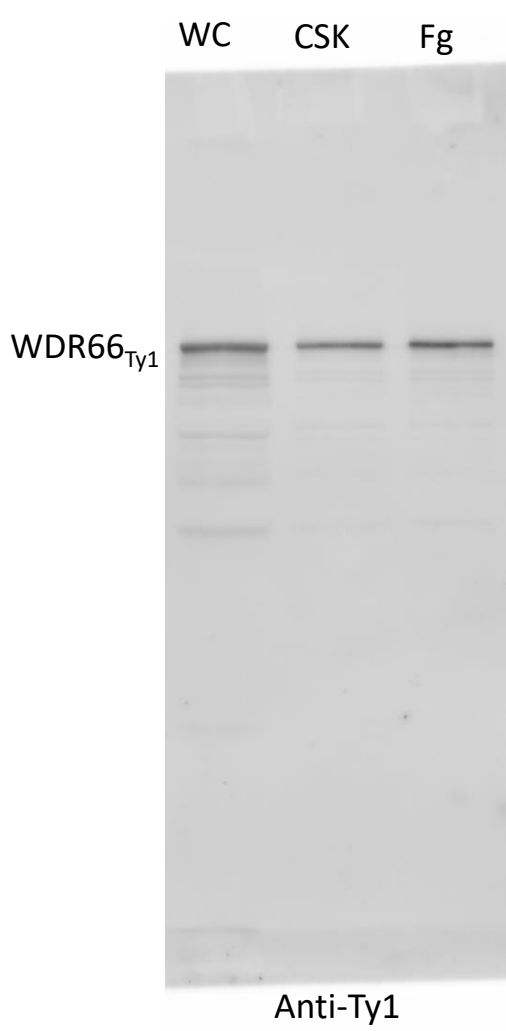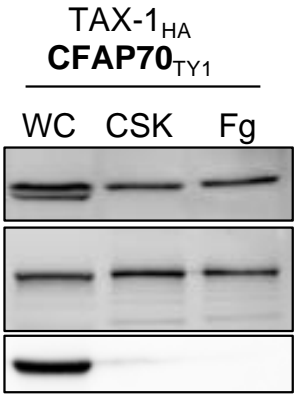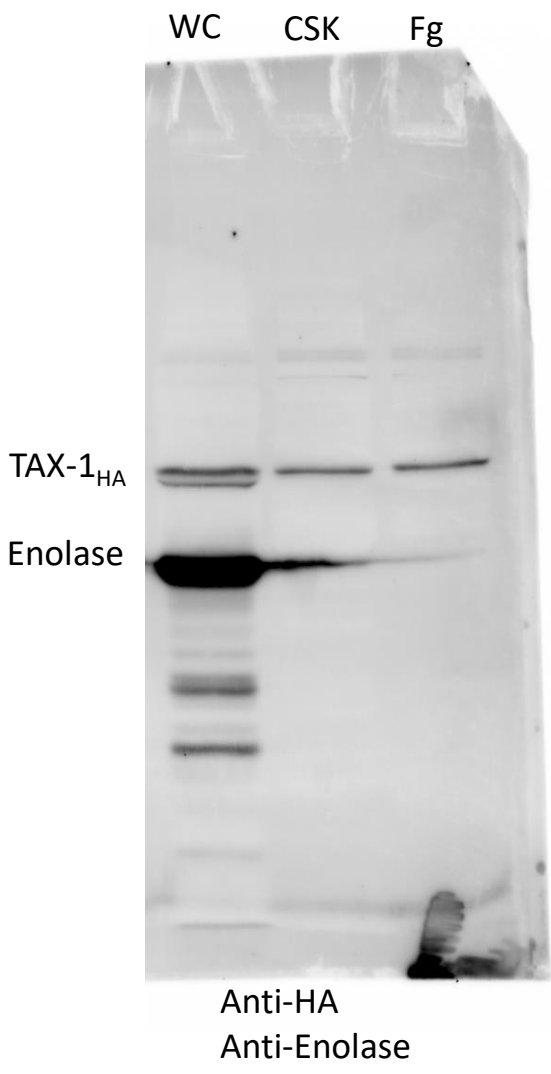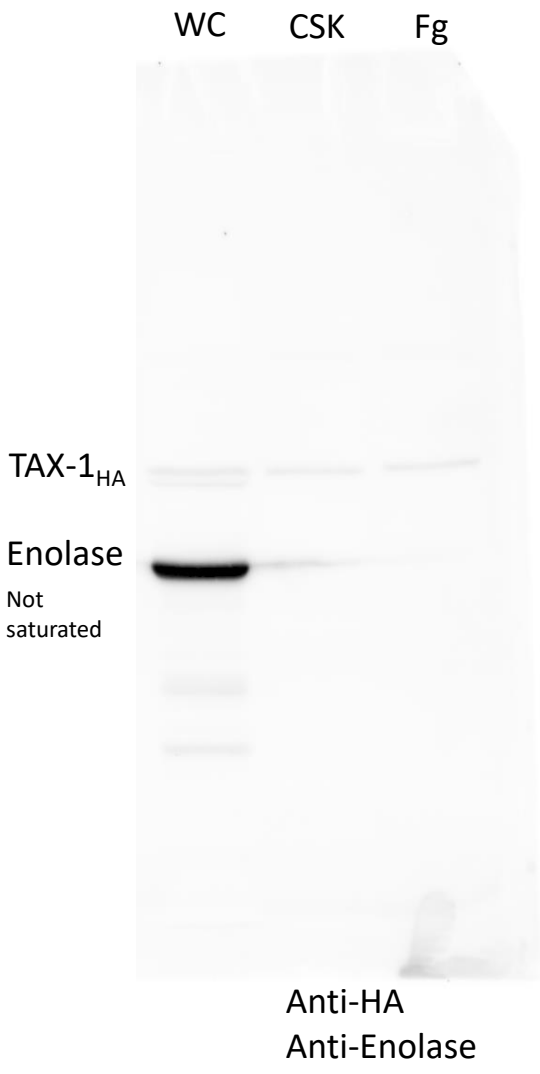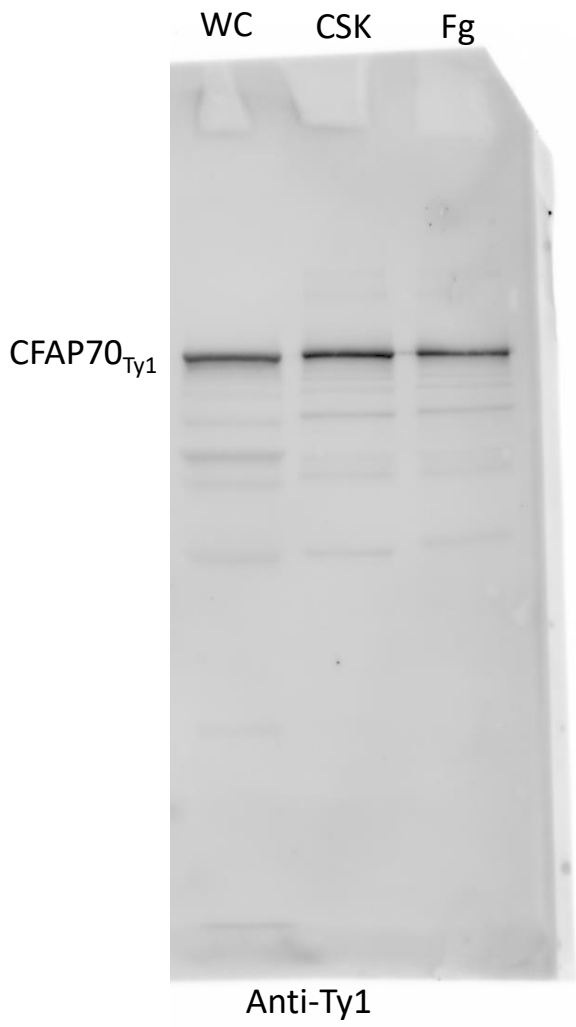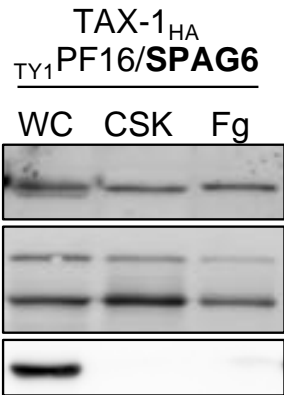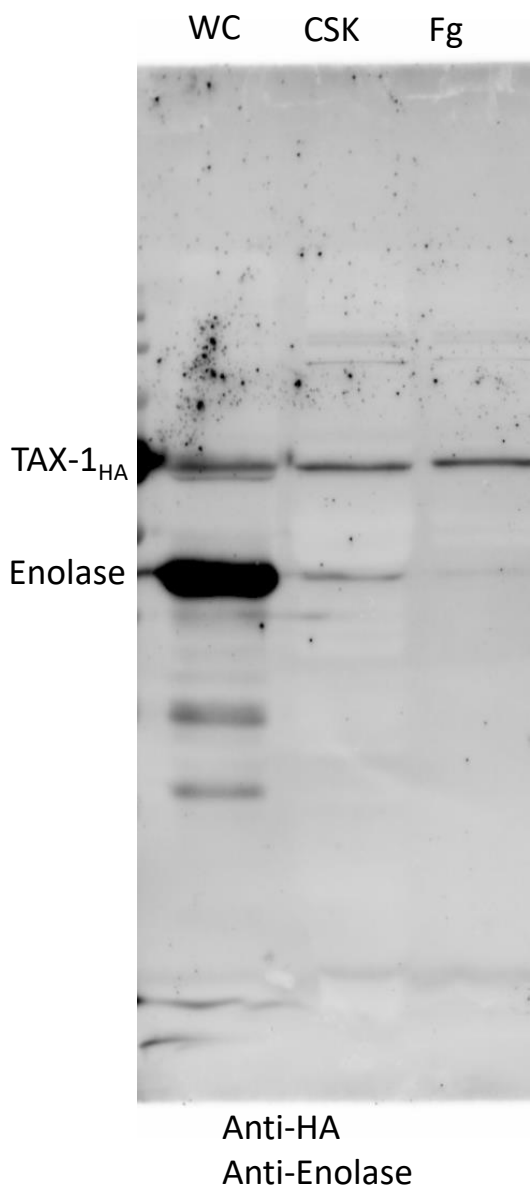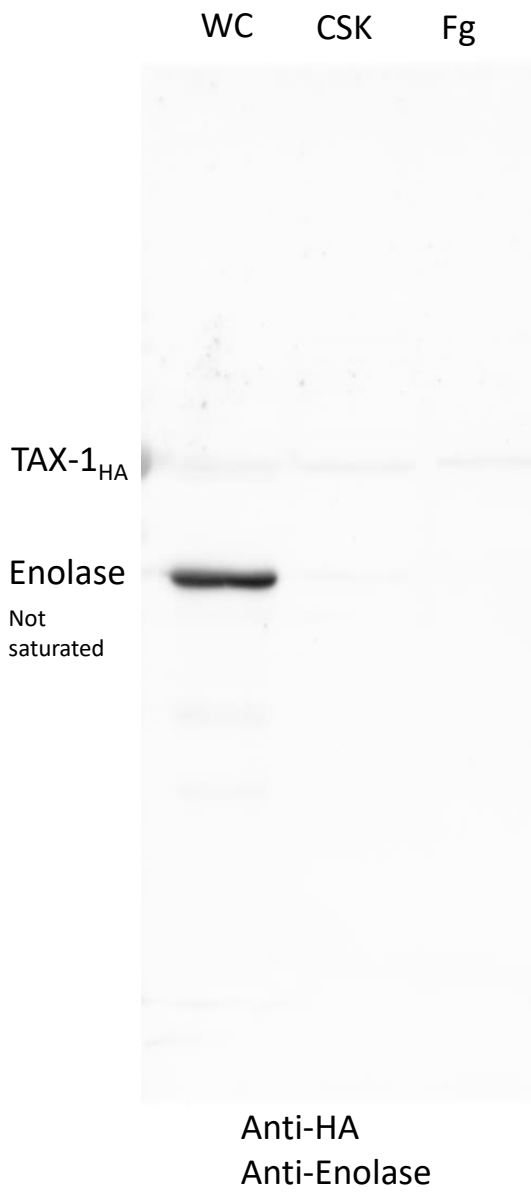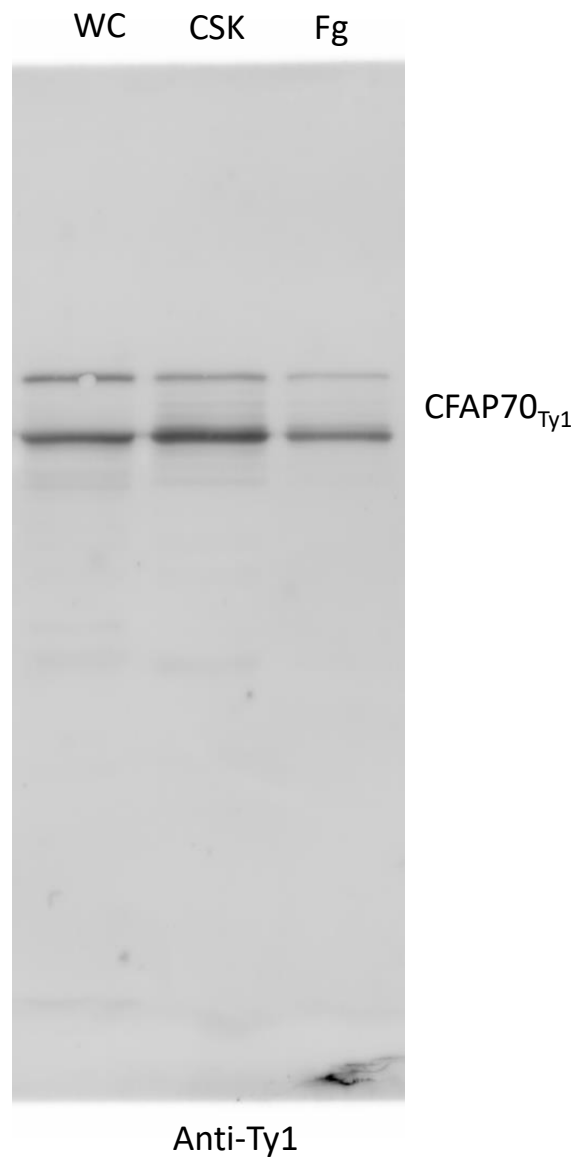

Supplement: Figure 4—figure supplement 1—source data 1. [file elife-87698-fig4-figsupp1-data1.zip › Figure 4-figure supplement 1-source data 1/Figure 4-supplement 1A-uncropped blots.pdf]

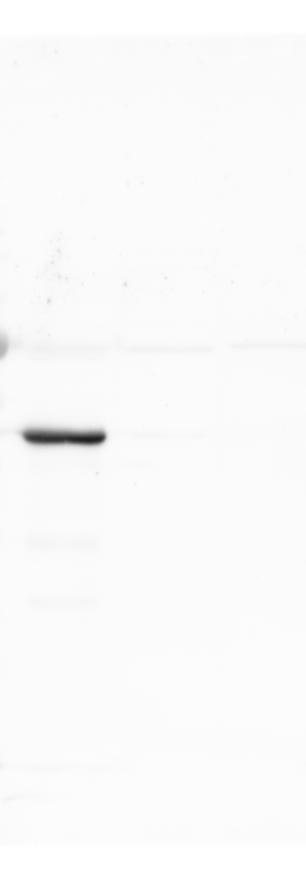

Supplement: Figure 4—figure supplement 1—source data 1. [file elife-87698-fig4-figsupp1-data1.zip › Figure 4-figure supplement 1-source data 1/Figure 4-supplement 1A-SPAG6-antiHA_antiEnolase not saturated.tif]

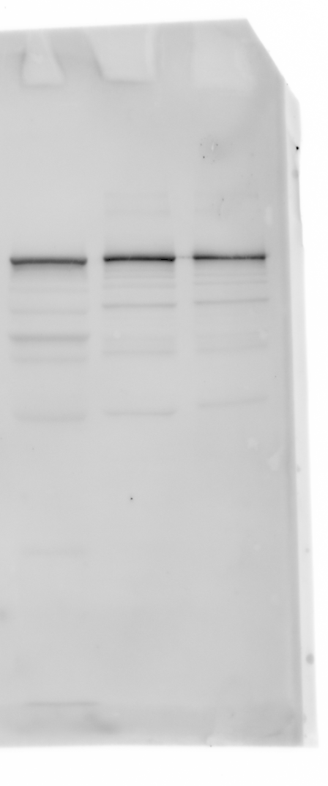

Supplement: Figure 4—figure supplement 1—source data 1. [file elife-87698-fig4-figsupp1-data1.zip › Figure 4-figure supplement 1-source data 1/Figure 4-supplement 1A-CFAP70-antiTy1.tif]

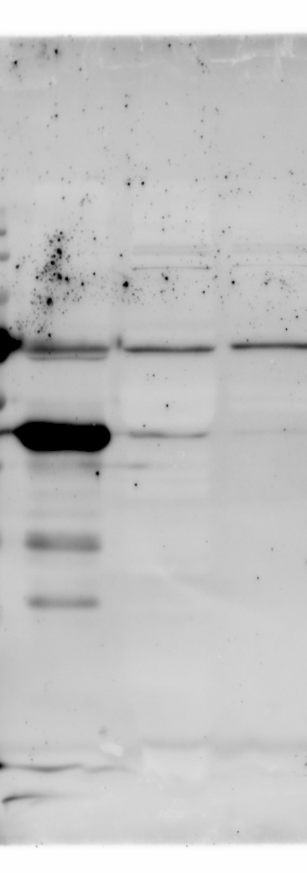

Supplement: Figure 4—figure supplement 1—source data 1. [file elife-87698-fig4-figsupp1-data1.zip › Figure 4-figure supplement 1-source data 1/Figure 4-supplement 1A-SPAG6-antiHA_antiEnolase saturated.tif]

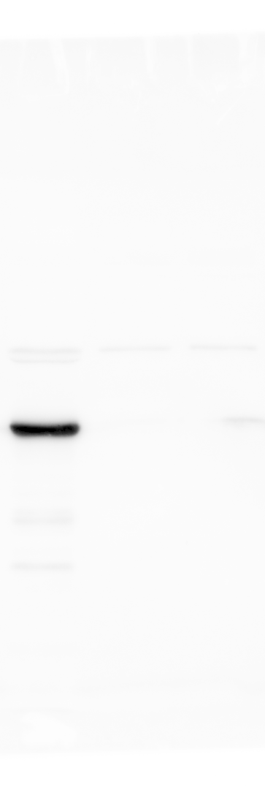

Supplement: Figure 4—figure supplement 1—source data 1. [file elife-87698-fig4-figsupp1-data1.zip › Figure 4-figure supplement 1-source data 1/Figure 4-supplement 1A-WDR66-antiHA_antiEnolase not saturated.tif]

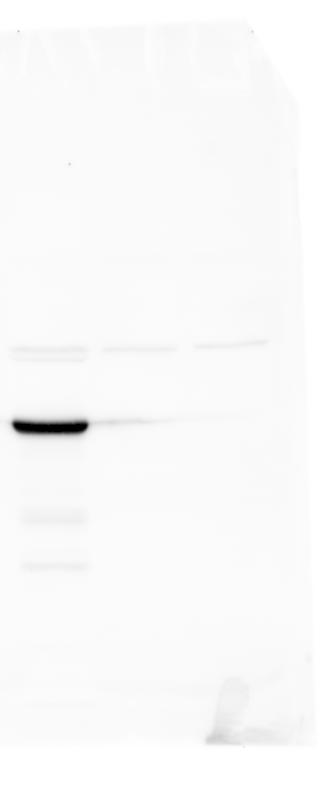

Supplement: Figure 4—figure supplement 1—source data 1. [file elife-87698-fig4-figsupp1-data1.zip › Figure 4-figure supplement 1-source data 1/Figure 4-supplement 1A-CFAP70-antiHA_antiEnolase not saturated.tif]

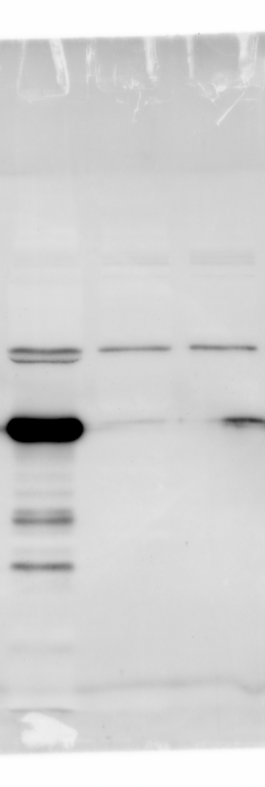

Supplement: Figure 4—figure supplement 1—source data 1. [file elife-87698-fig4-figsupp1-data1.zip › Figure 4-figure supplement 1-source data 1/Figure 4-supplement 1A-WDR66-antiHA_antiEnolase saturated.tif]

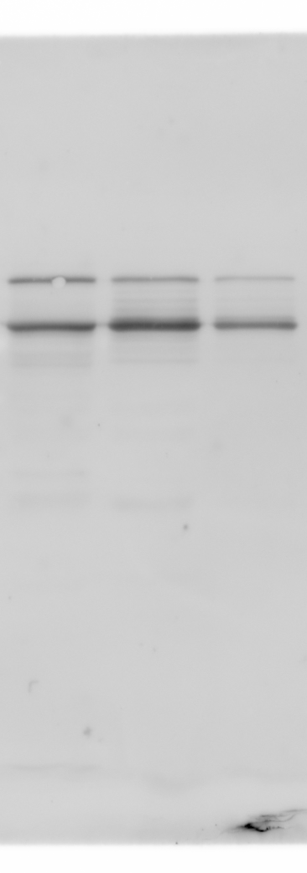

Supplement: Figure 4—figure supplement 1—source data 1. [file elife-87698-fig4-figsupp1-data1.zip › Figure 4-figure supplement 1-source data 1/Figure 4-supplement 1A-SPAG6-antiTy1.tif]

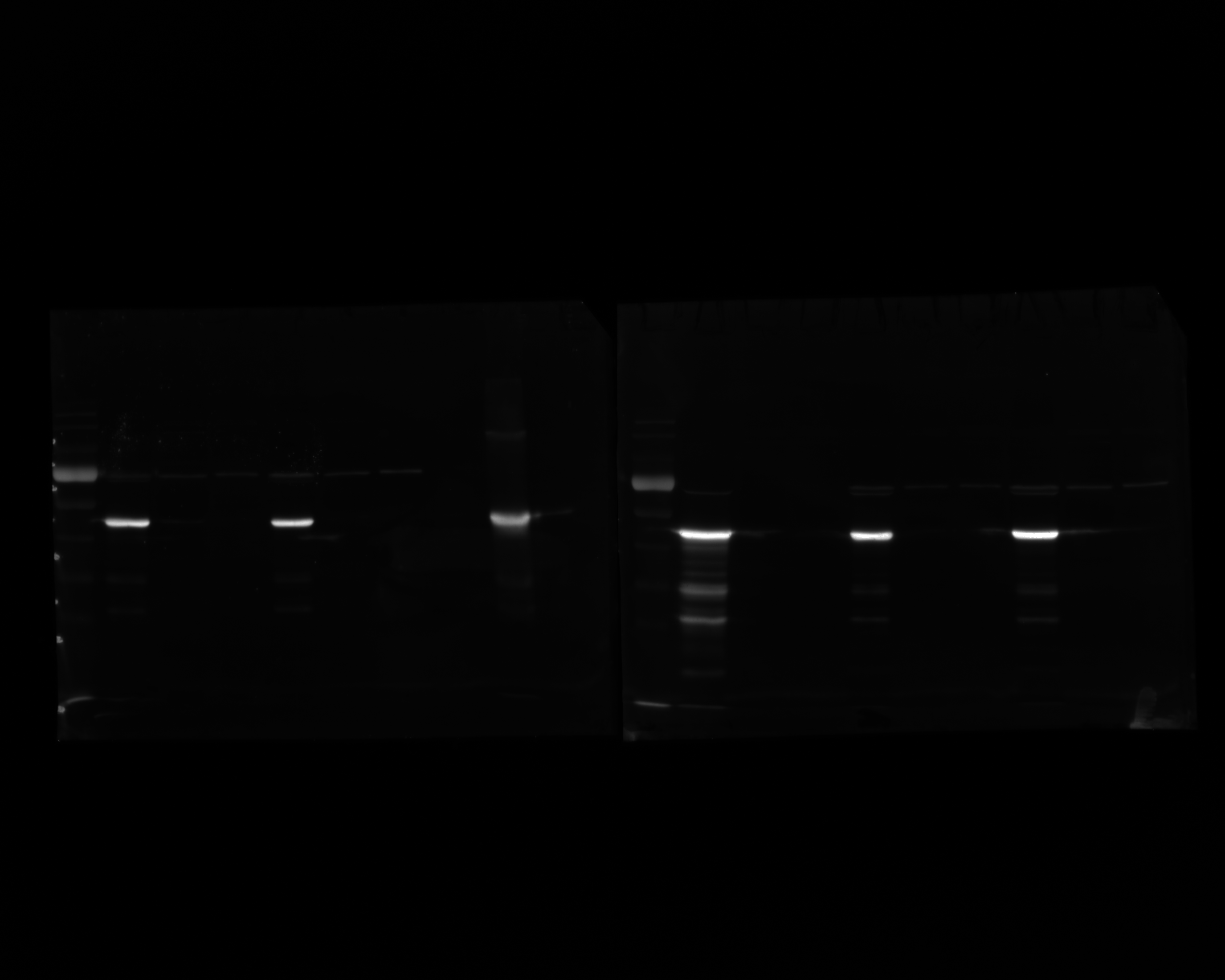

Supplement: Figure 4—figure supplement 1—source data 1. [file elife-87698-fig4-figsupp1-data1.zip › Figure 4-figure supplement 1-source data 1/Raw images/Figure 4-supplement 1A-Enolase.tif]

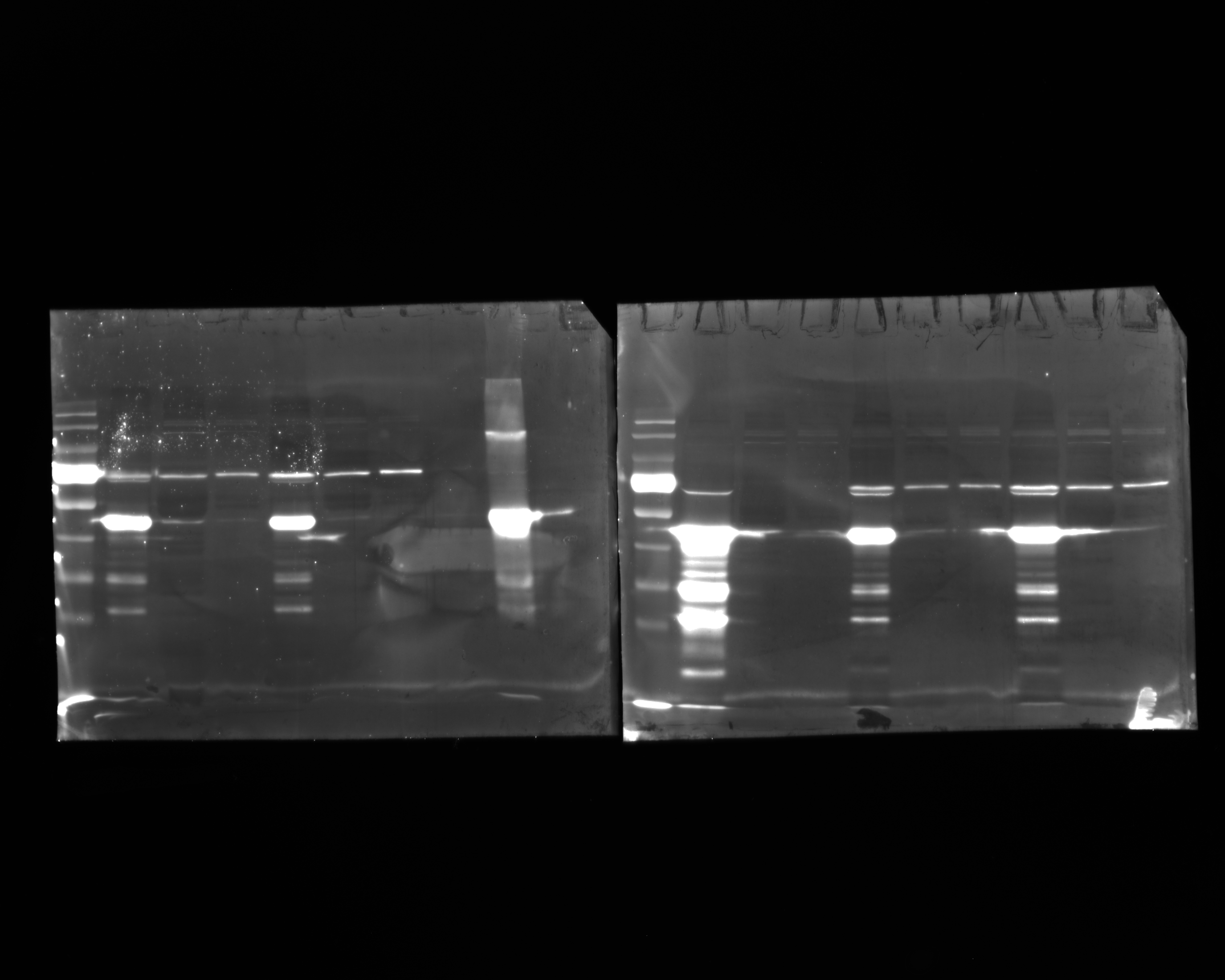

Supplement: Figure 4—figure supplement 1—source data 1. [file elife-87698-fig4-figsupp1-data1.zip › Figure 4-figure supplement 1-source data 1/Raw images/Figure 4-supplement 1A-TAX1.tif]

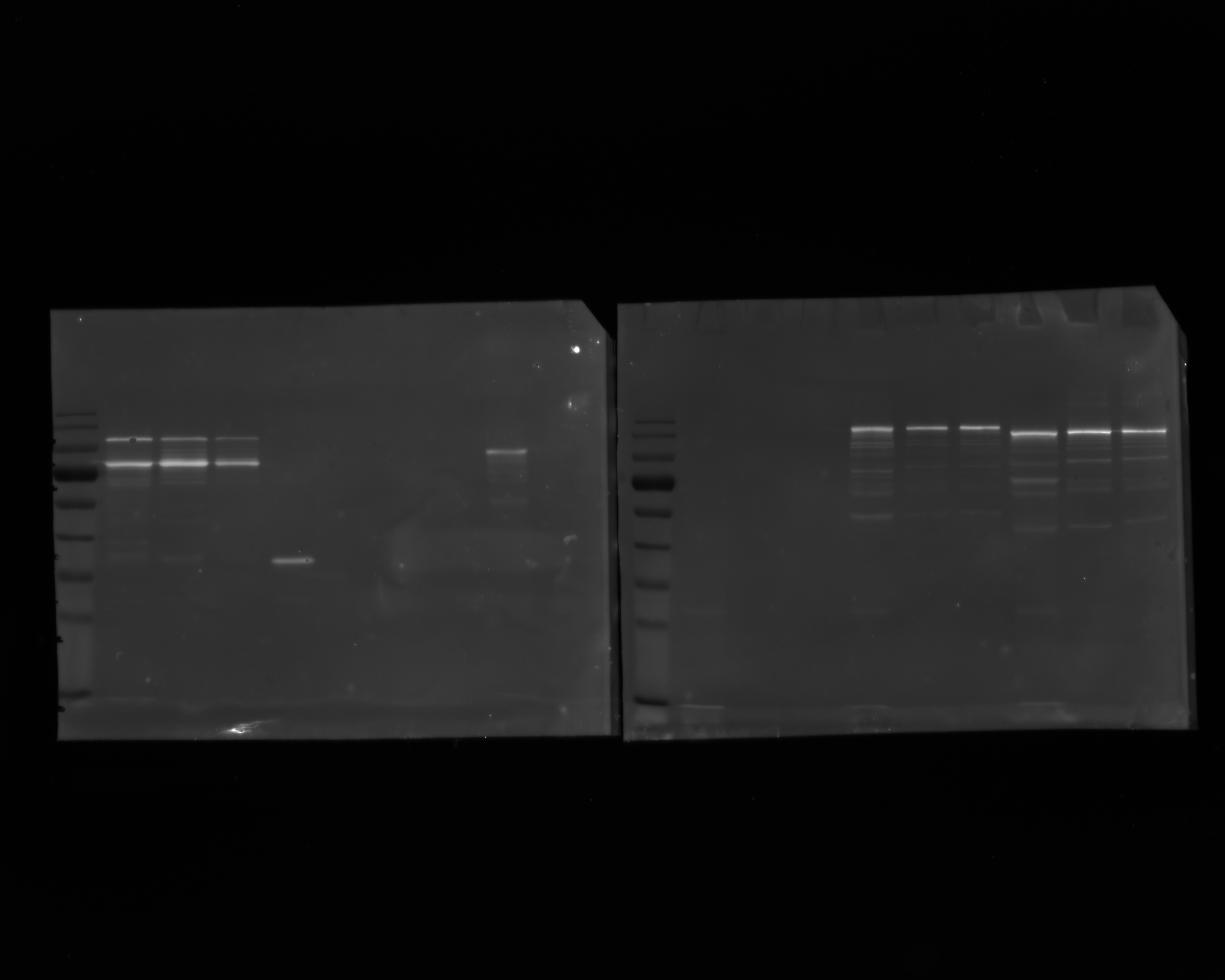

Supplement: Figure 4—figure supplement 1—source data 1. [file elife-87698-fig4-figsupp1-data1.zip › Figure 4-figure supplement 1-source data 1/Raw images/Figure 4-supplement 1A-WDR66_CFAP70_SPAG6.tif]

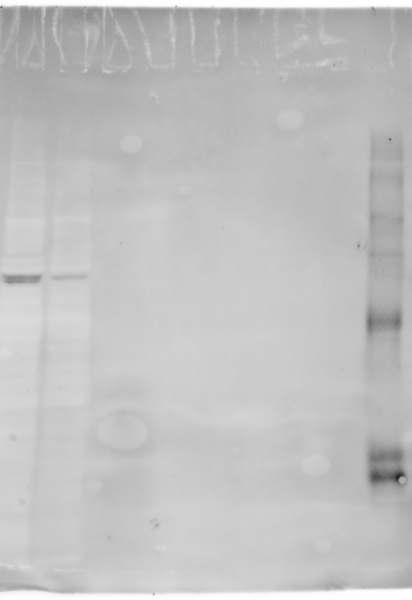

Supplement: Figure 4—figure supplement 1—source data 2. [file elife-87698-fig4-figsupp1-data2.zip › Figure 4-figure supplement1-source data 2/Figure 4-supplement 1B-SPAG6TY1_TAX1HA-antiHA.tif]

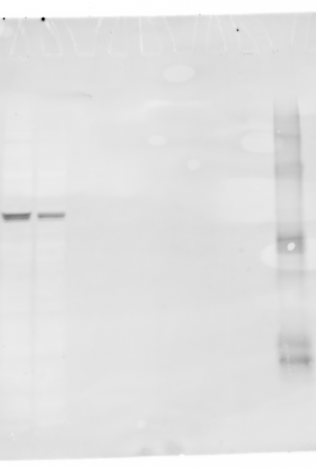

Supplement: Figure 4—figure supplement 1—source data 2. [file elife-87698-fig4-figsupp1-data2.zip › Figure 4-figure supplement1-source data 2/Figure 4-supplement 1B-WDR66TY1_TAX1HA-antiHA.tif]

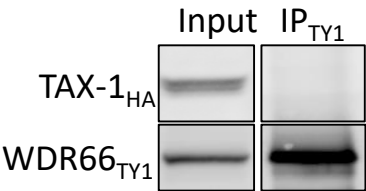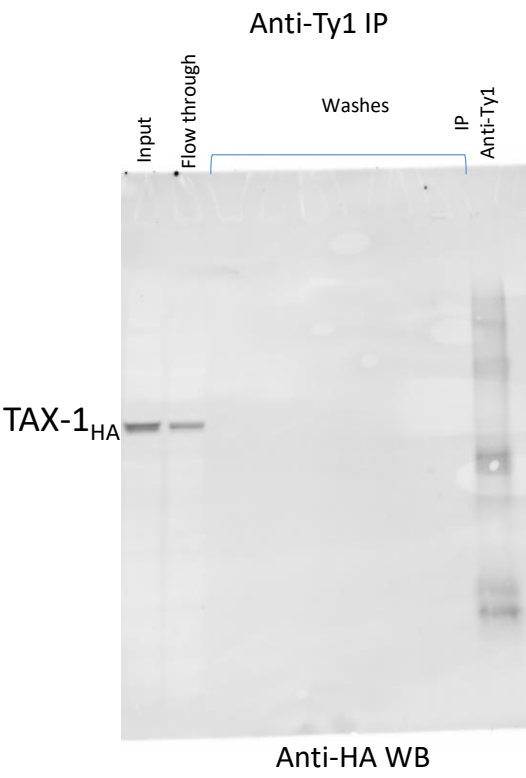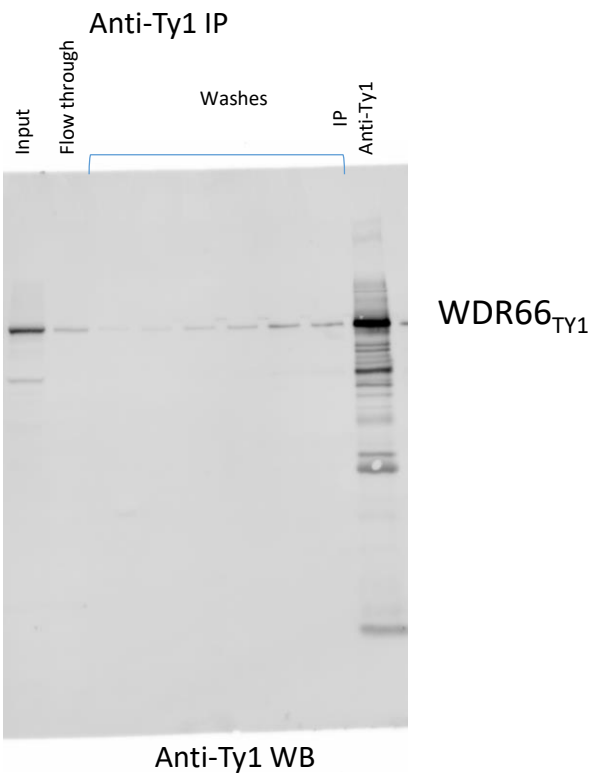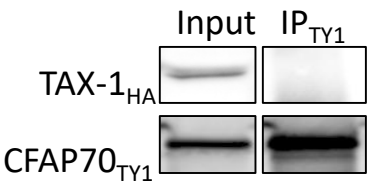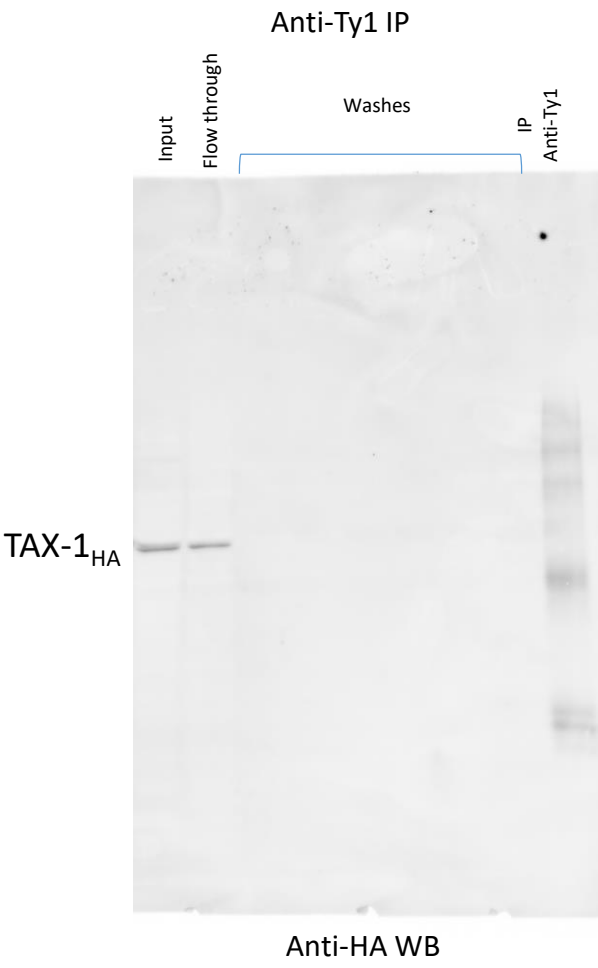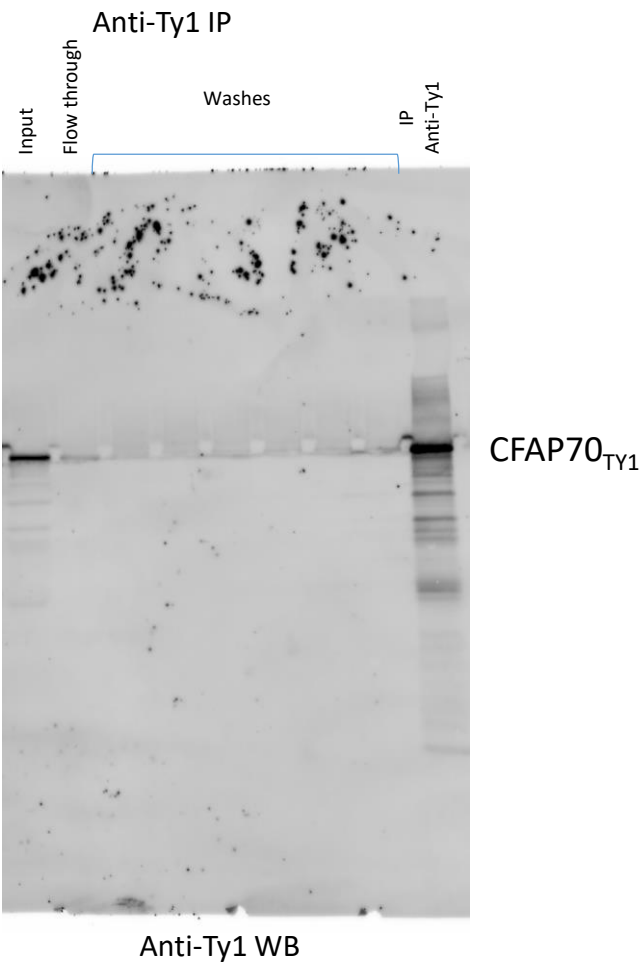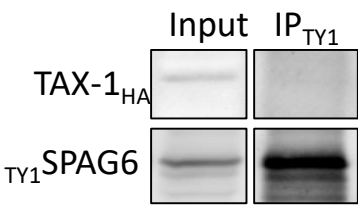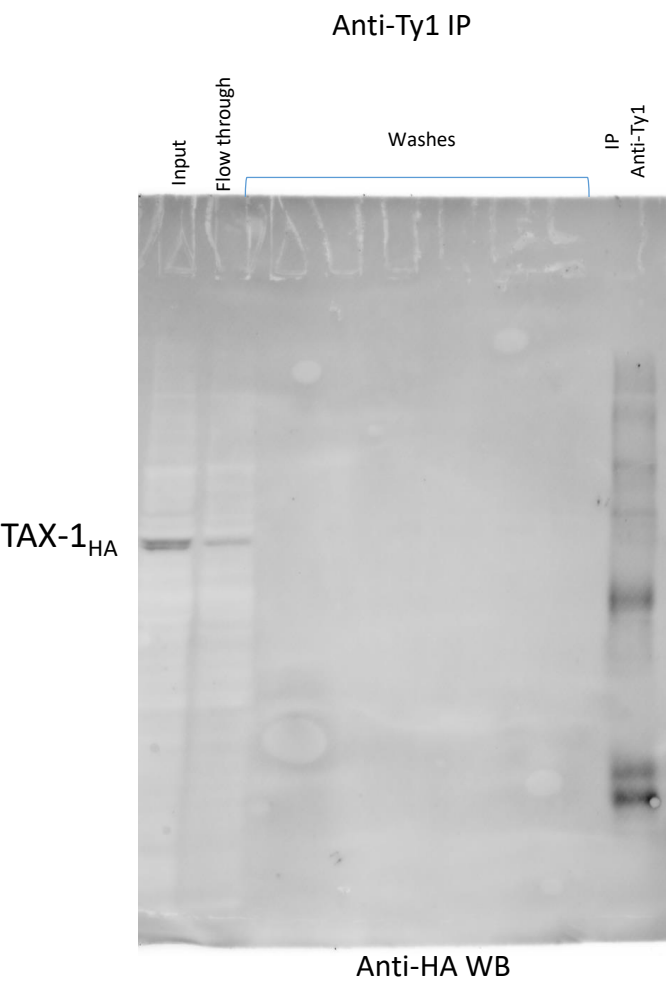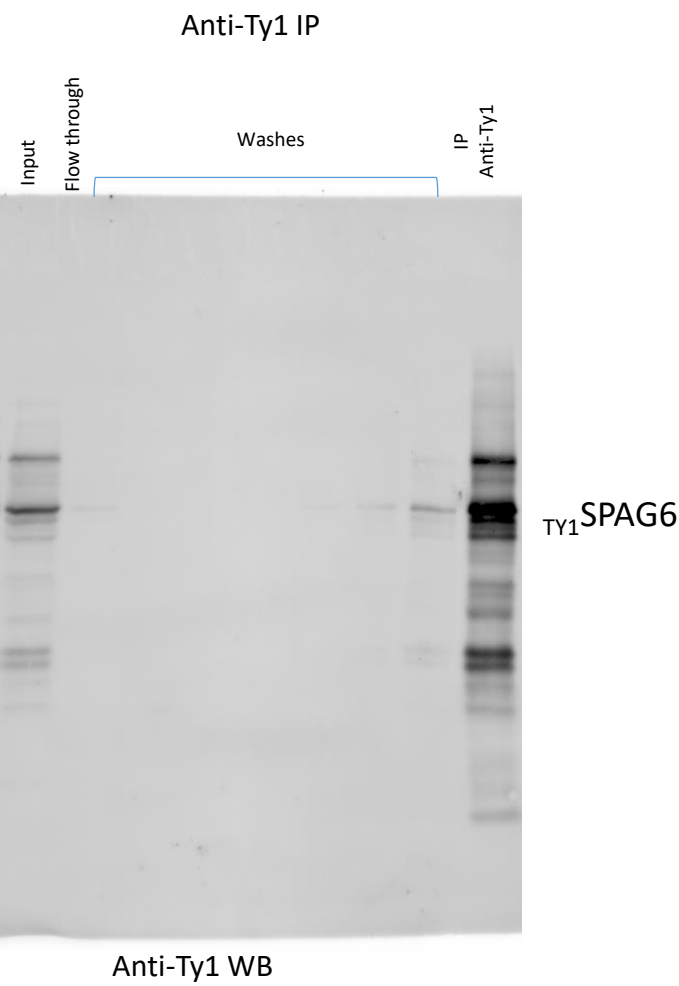

Supplement: Figure 4—figure supplement 1—source data 2. [file elife-87698-fig4-figsupp1-data2.zip › Figure 4-figure supplement1-source data 2/Figure 4-supplement 1B-uncropped blots.pdf]

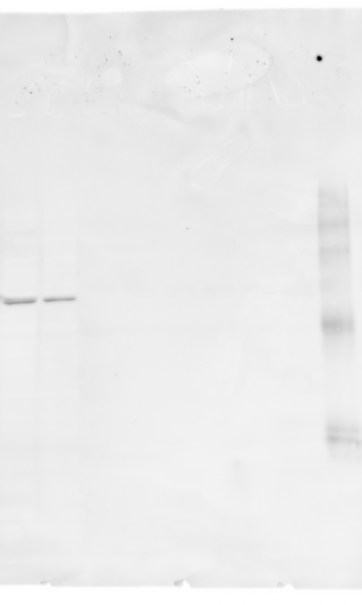

Supplement: Figure 4—figure supplement 1—source data 2. [file elife-87698-fig4-figsupp1-data2.zip › Figure 4-figure supplement1-source data 2/Figure 4-supplement 1B-CFAP70TY1_TAX1HA-antiHA.tif]

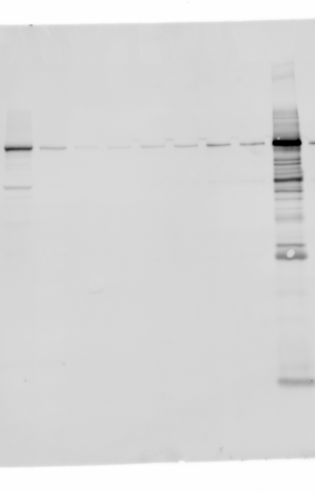

Supplement: Figure 4—figure supplement 1—source data 2. [file elife-87698-fig4-figsupp1-data2.zip › Figure 4-figure supplement1-source data 2/Figure 4-supplement 1B-WDR66TY1_TAX1HA-antiTy1.tif]

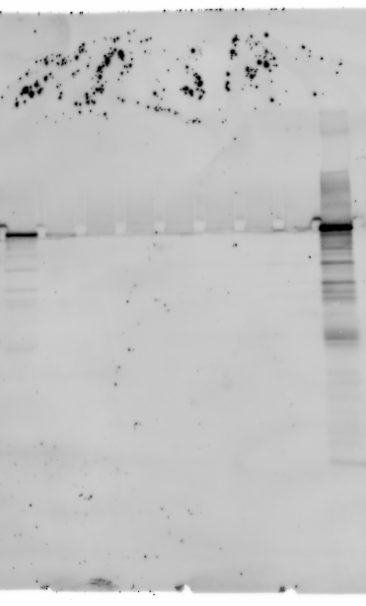

Supplement: Figure 4—figure supplement 1—source data 2. [file elife-87698-fig4-figsupp1-data2.zip › Figure 4-figure supplement1-source data 2/Figure 4-supplement 1B-CFAP70TY1_TAX1HA-antiTy1.tif]

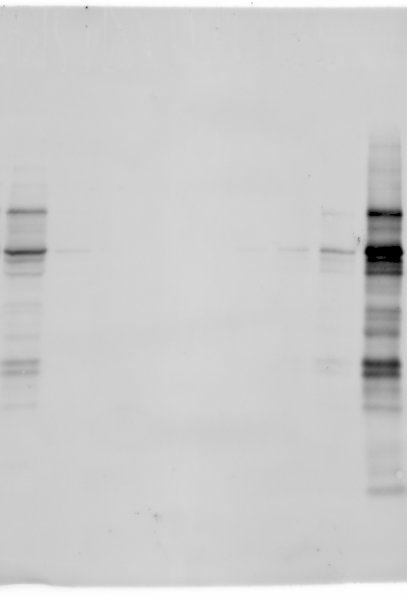

Supplement: Figure 4—figure supplement 1—source data 2. [file elife-87698-fig4-figsupp1-data2.zip › Figure 4-figure supplement1-source data 2/Figure 4-supplement 1B-SPAG6TY1_TAX1HA-antiTy1.tif]

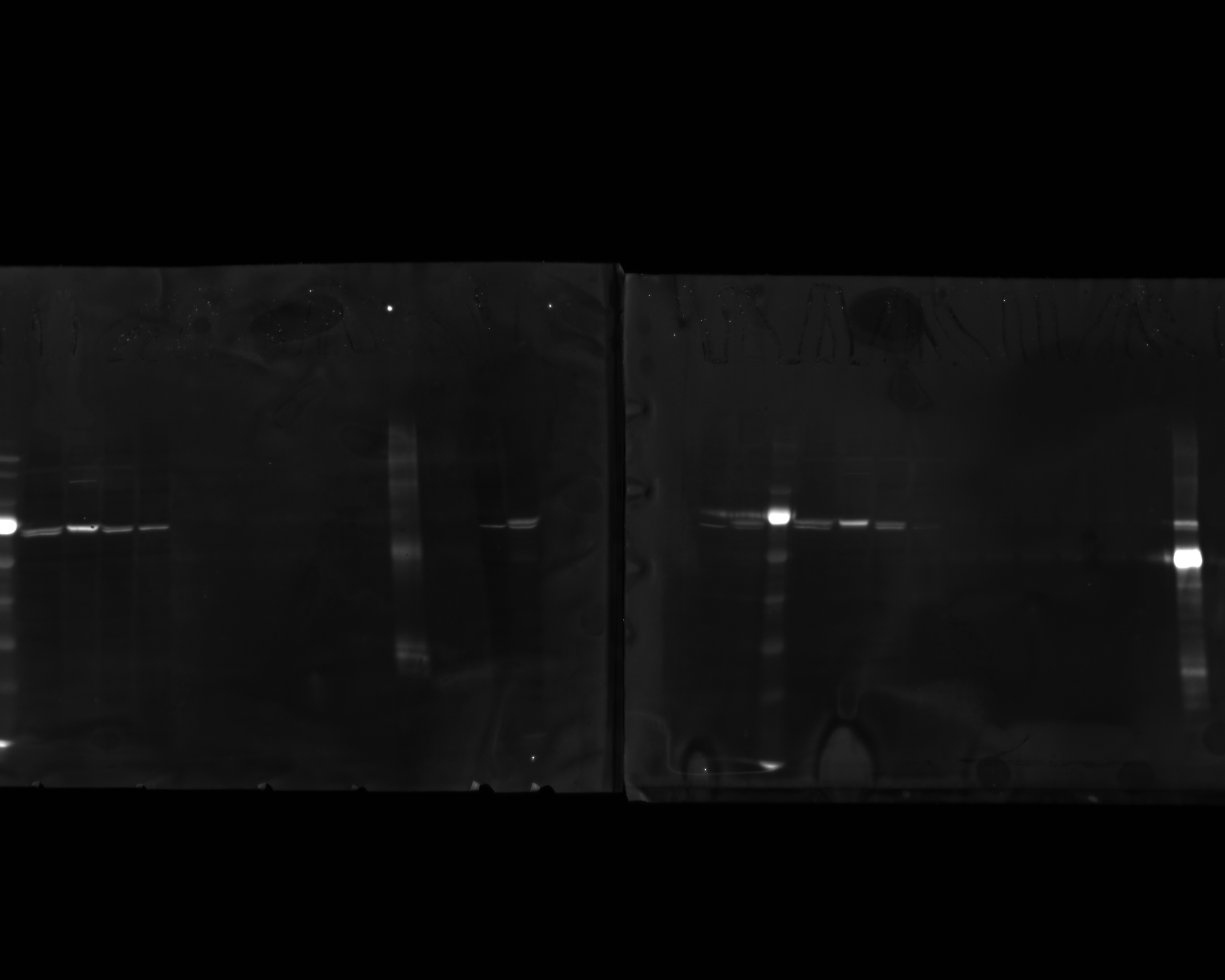

Supplement: Figure 4—figure supplement 1—source data 2. [file elife-87698-fig4-figsupp1-data2.zip › Figure 4-figure supplement1-source data 2/Raw images/co-IP TAX1_CFAP70/Figure 4-supplement 1B-TAX1 (coIP CFAP70).tif]

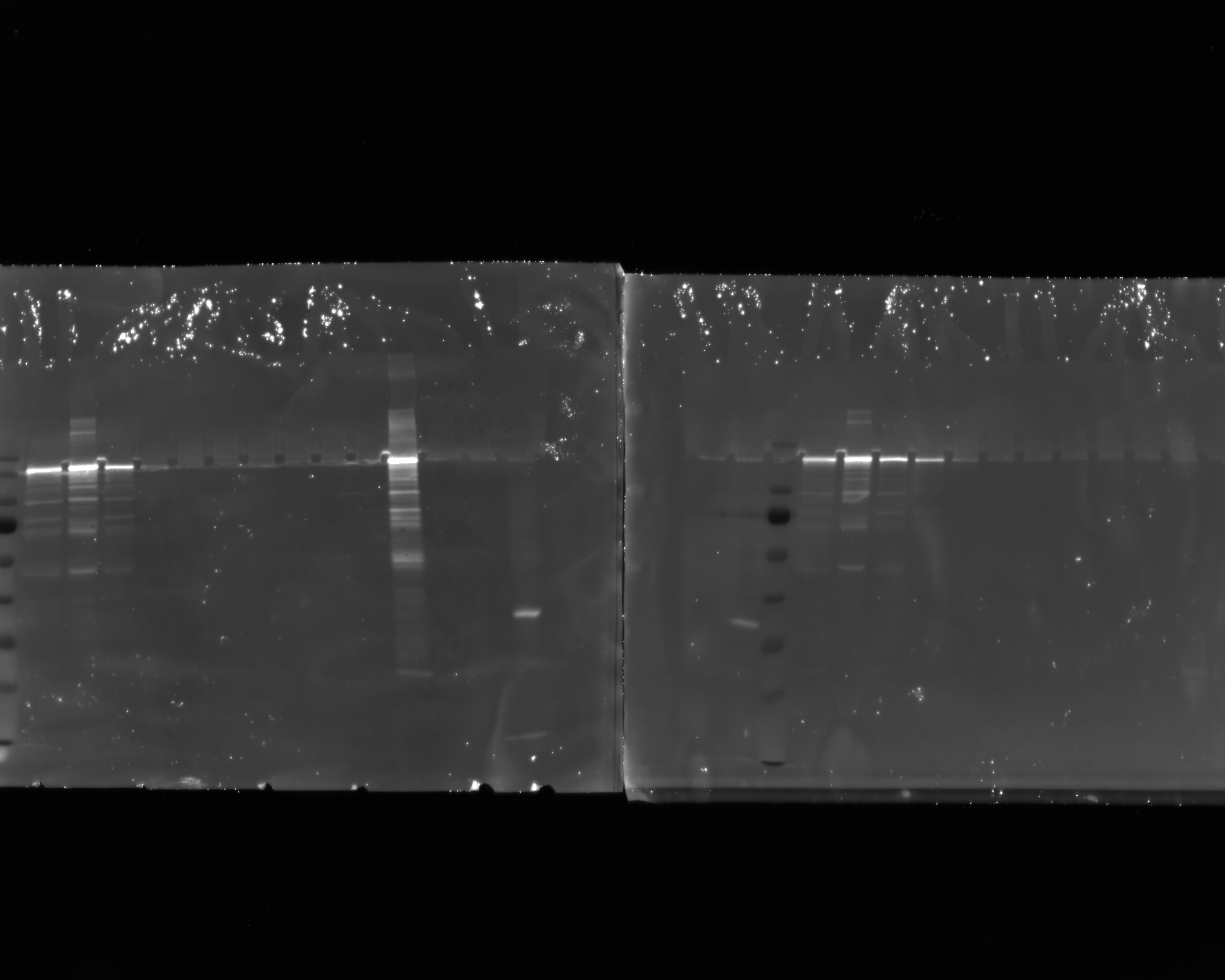

Supplement: Figure 4—figure supplement 1—source data 2. [file elife-87698-fig4-figsupp1-data2.zip › Figure 4-figure supplement1-source data 2/Raw images/co-IP TAX1_CFAP70/Figure 4-supplement 1B-CFAP70.tif]

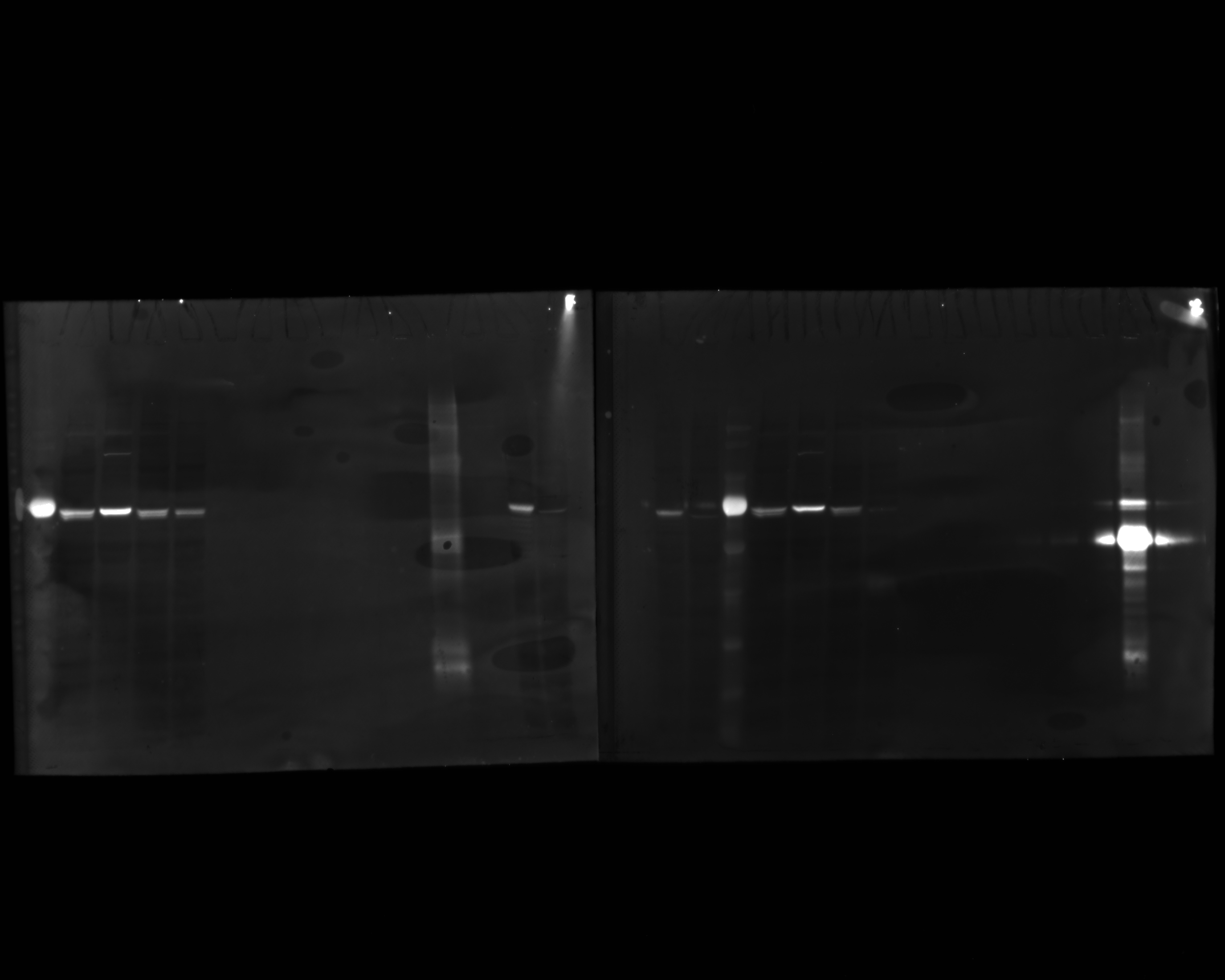

Supplement: Figure 4—figure supplement 1—source data 2. [file elife-87698-fig4-figsupp1-data2.zip › Figure 4-figure supplement1-source data 2/Raw images/co-IP TAX1_WDR66/Figure 4-supplerment 1B-TAX1 (coIP WDR66).tif]

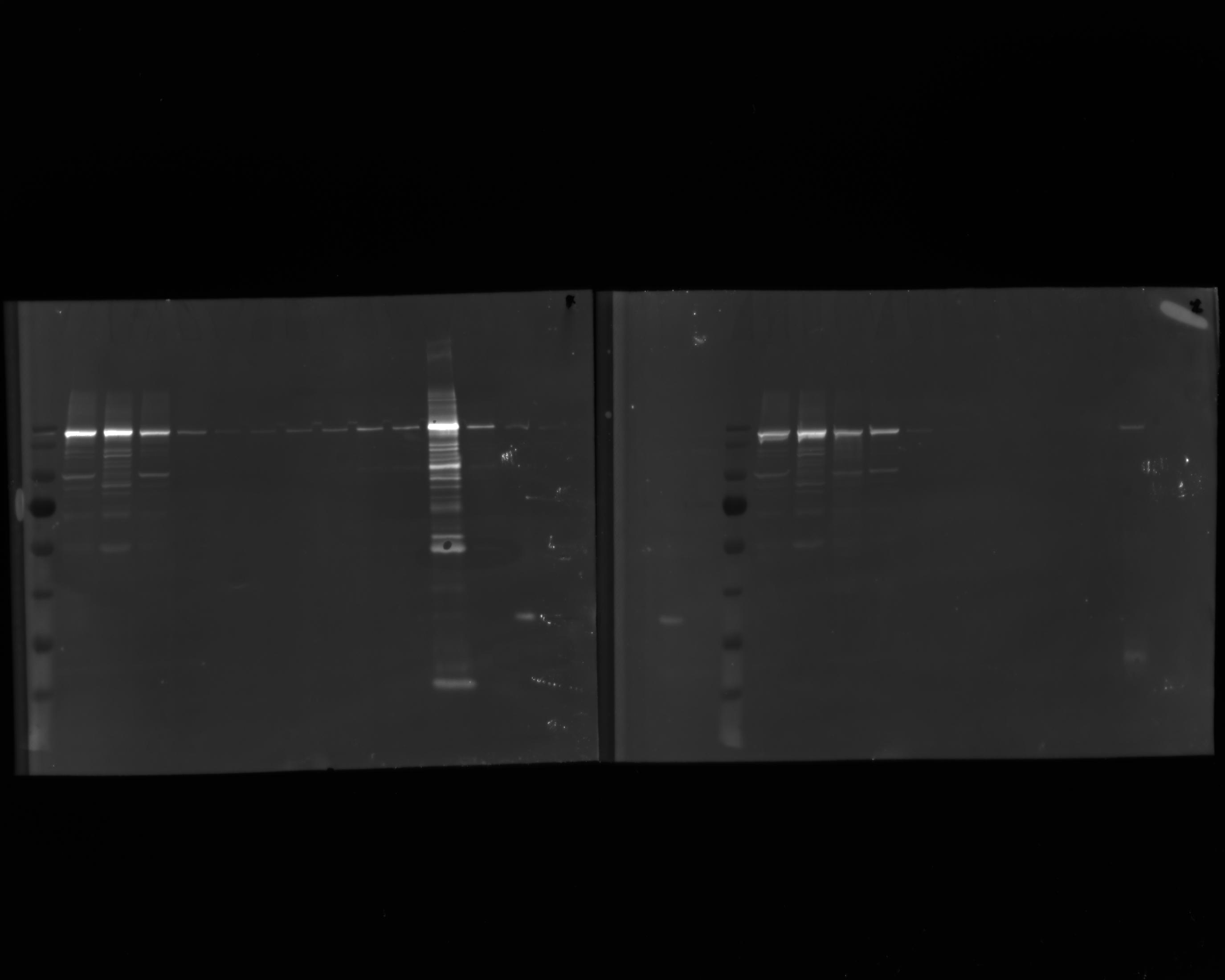

Supplement: Figure 4—figure supplement 1—source data 2. [file elife-87698-fig4-figsupp1-data2.zip › Figure 4-figure supplement1-source data 2/Raw images/co-IP TAX1_WDR66/Figure 4-supplement 1B-WDR66.tif]

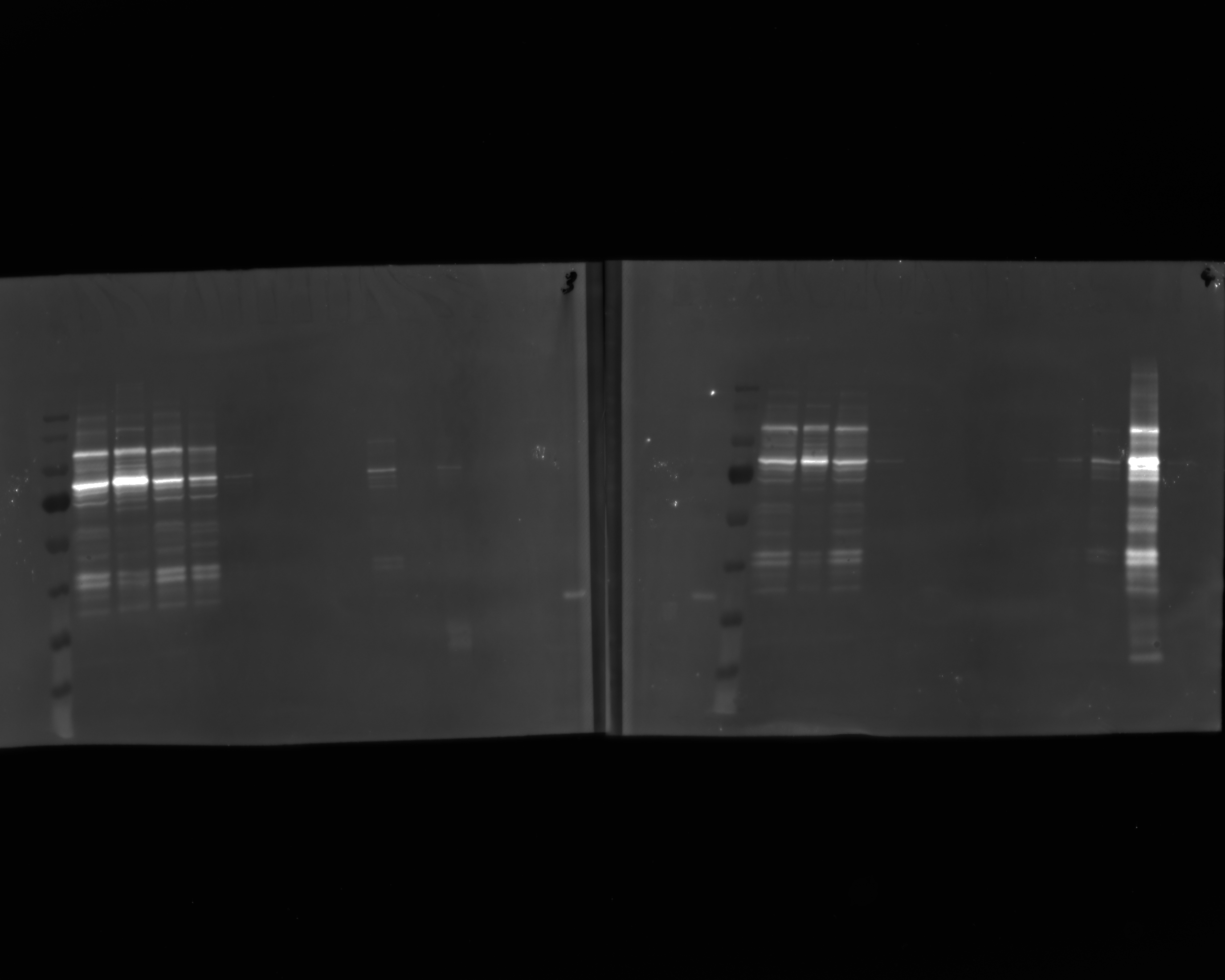

Supplement: Figure 4—figure supplement 1—source data 2. [file elife-87698-fig4-figsupp1-data2.zip › Figure 4-figure supplement1-source data 2/Raw images/co-IP TAX1_SPAG6/Figure 4-supplement 1B-SPAG6.tif]

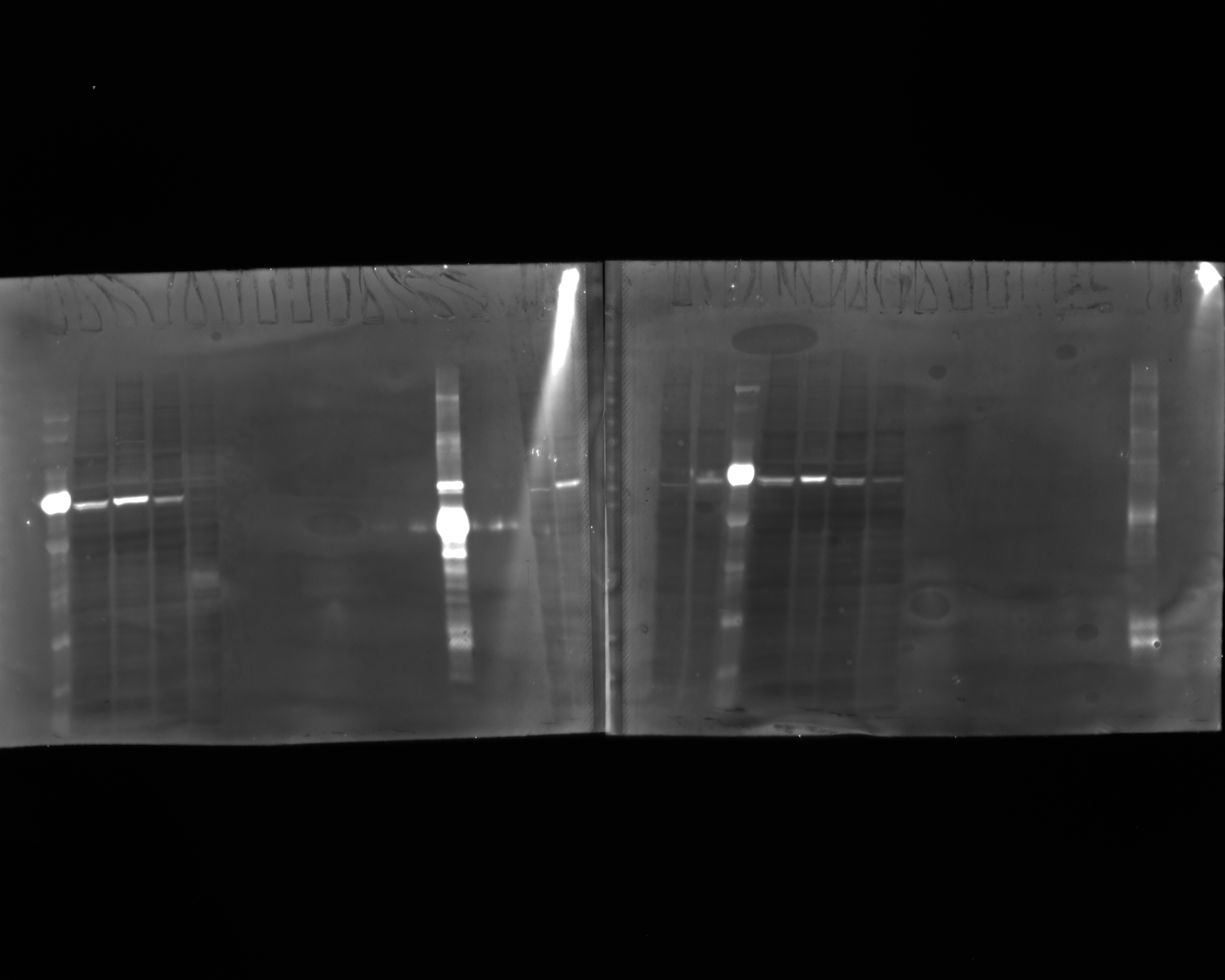

Supplement: Figure 4—figure supplement 1—source data 2. [file elife-87698-fig4-figsupp1-data2.zip › Figure 4-figure supplement1-source data 2/Raw images/co-IP TAX1_SPAG6/Figure 4-supplement 1B-TAX1 (coIP SPAG6).tif]

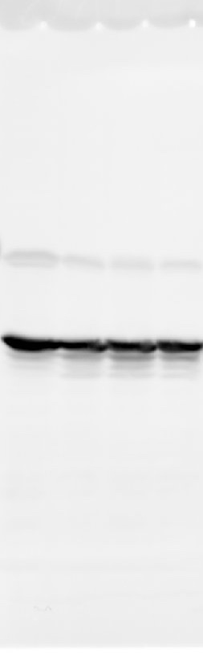

Supplement: Figure 4—figure supplement 1—source data 3. [file elife-87698-fig4-figsupp1-data3.zip › Figure 4-figure supplement 1-source data 3/Figure 4-supplement 1C-WDR66TY1_TAX1HA-antiHA-antiEnolase not saturated.tif]

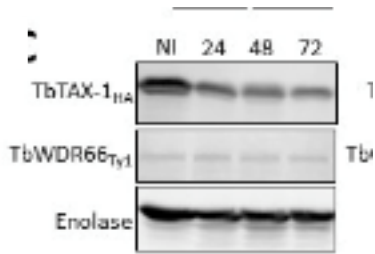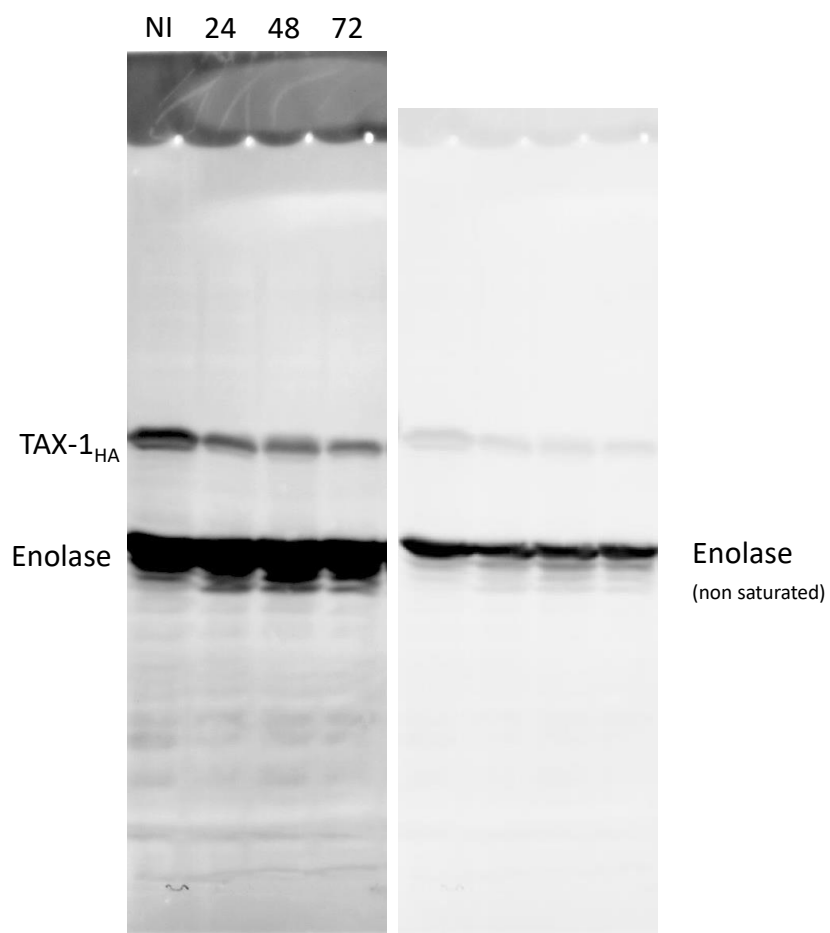

Anti-HA + anti-Enolase

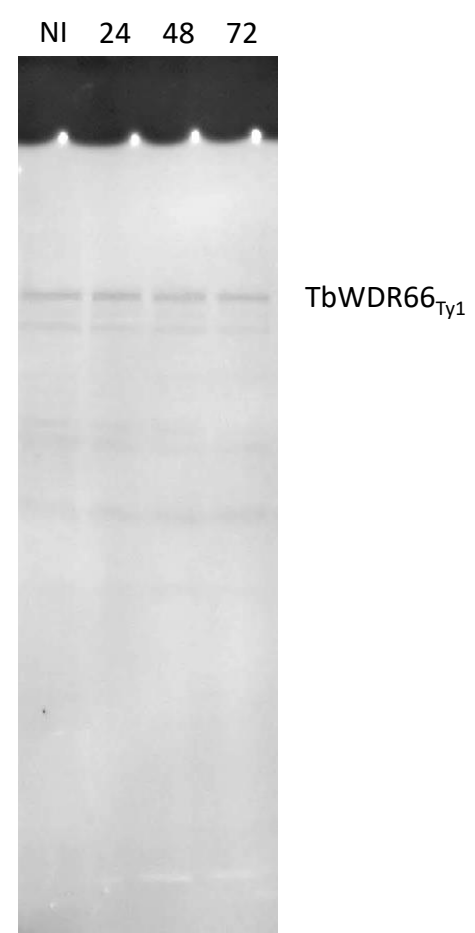

Anti-Ty1

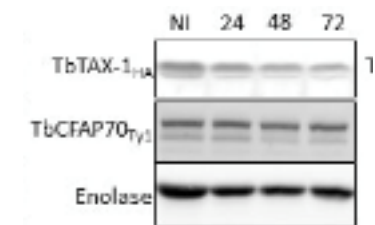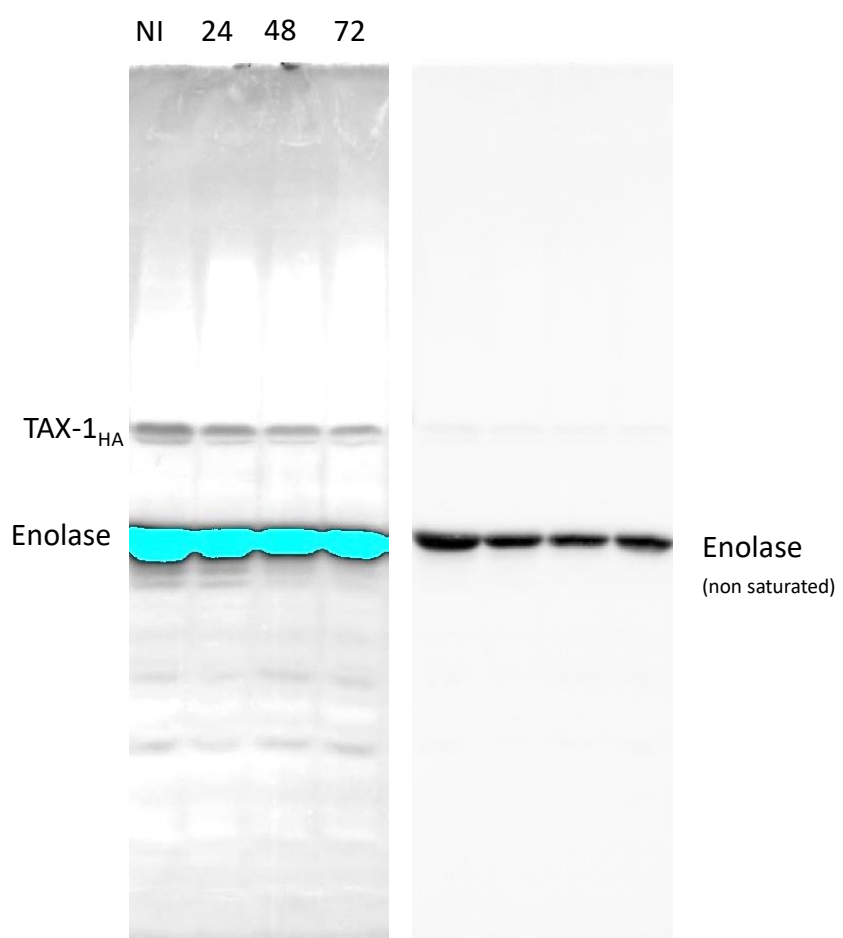

Anti-HA + anti-Enolase

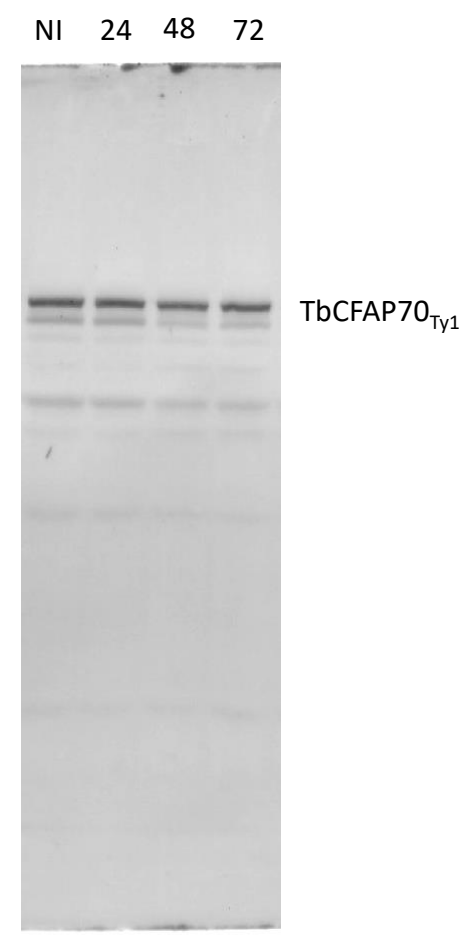

Anti-Ty1

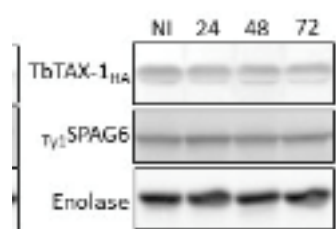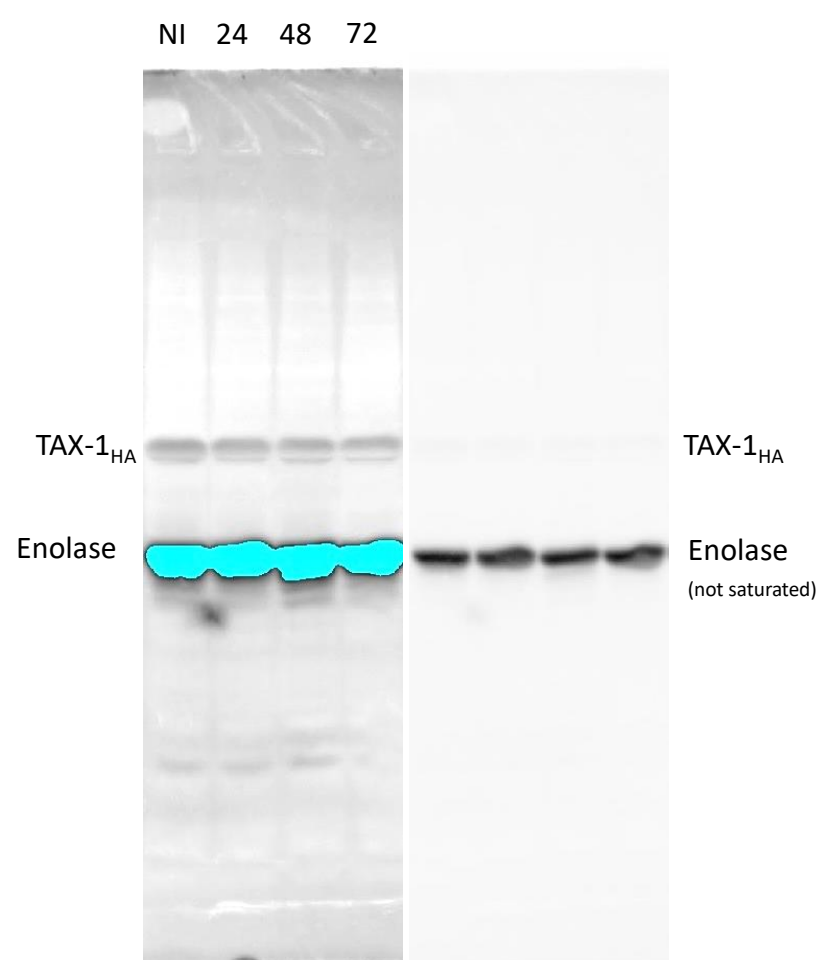

Anti-HA + anti-Enolase

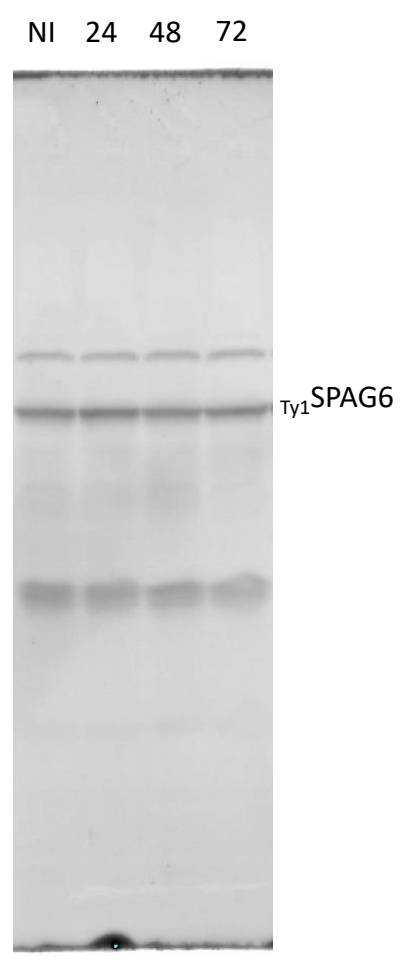

Anti-Ty1

Supplement: Figure 4—figure supplement 1—source data 3. [file elife-87698-fig4-figsupp1-data3.zip › Figure 4-figure supplement 1-source data 3/Figure 4-supplement 1C-uncropped blots.pdf]

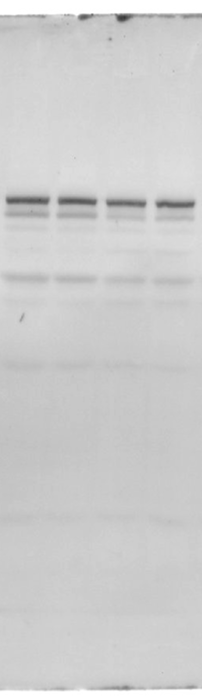

Supplement: Figure 4—figure supplement 1—source data 3. [file elife-87698-fig4-figsupp1-data3.zip › Figure 4-figure supplement 1-source data 3/Figure 4-supplement 1C-CFAP70TY1_TAX1HA-antiTY1.tif]

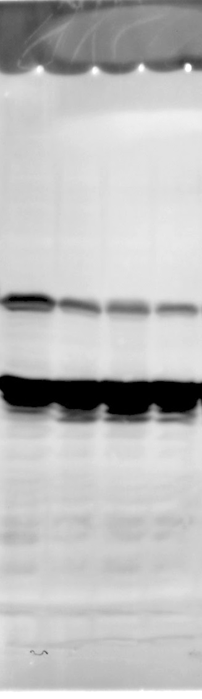

Supplement: Figure 4—figure supplement 1—source data 3. [file elife-87698-fig4-figsupp1-data3.zip › Figure 4-figure supplement 1-source data 3/Figure 4-supplement 1C-WDR66TY1_TAX1HA-antiHA-antiEnolase saturated.tif]

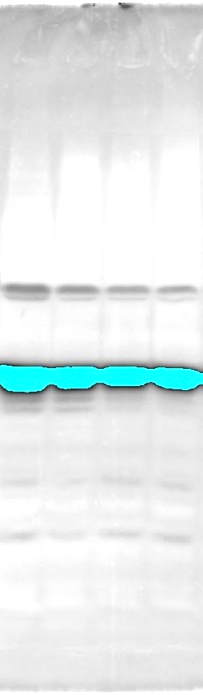

Supplement: Figure 4—figure supplement 1—source data 3. [file elife-87698-fig4-figsupp1-data3.zip › Figure 4-figure supplement 1-source data 3/Figure 4-supplement 1C-CFAP70TY1_TAX1HA-antiHA-antiEnolase saturated.tif]

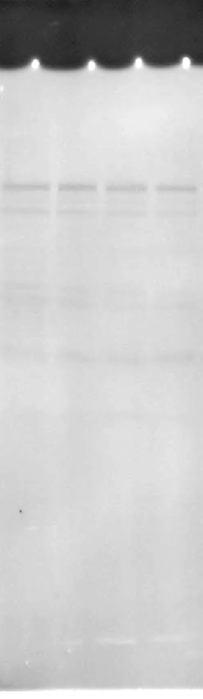

Supplement: Figure 4—figure supplement 1—source data 3. [file elife-87698-fig4-figsupp1-data3.zip › Figure 4-figure supplement 1-source data 3/Figure 4-supplement 1C-WDR66TY1_TAX1HA-antiTY1.tif]

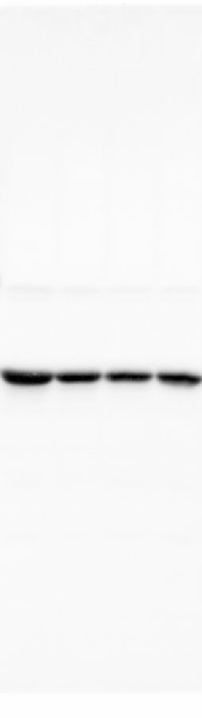

Supplement: Figure 4—figure supplement 1—source data 3. [file elife-87698-fig4-figsupp1-data3.zip › Figure 4-figure supplement 1-source data 3/Figure 4-supplement 1C-CFAP70TY1_TAX1HA-antiHA-antiEnolase not saturated.tif]

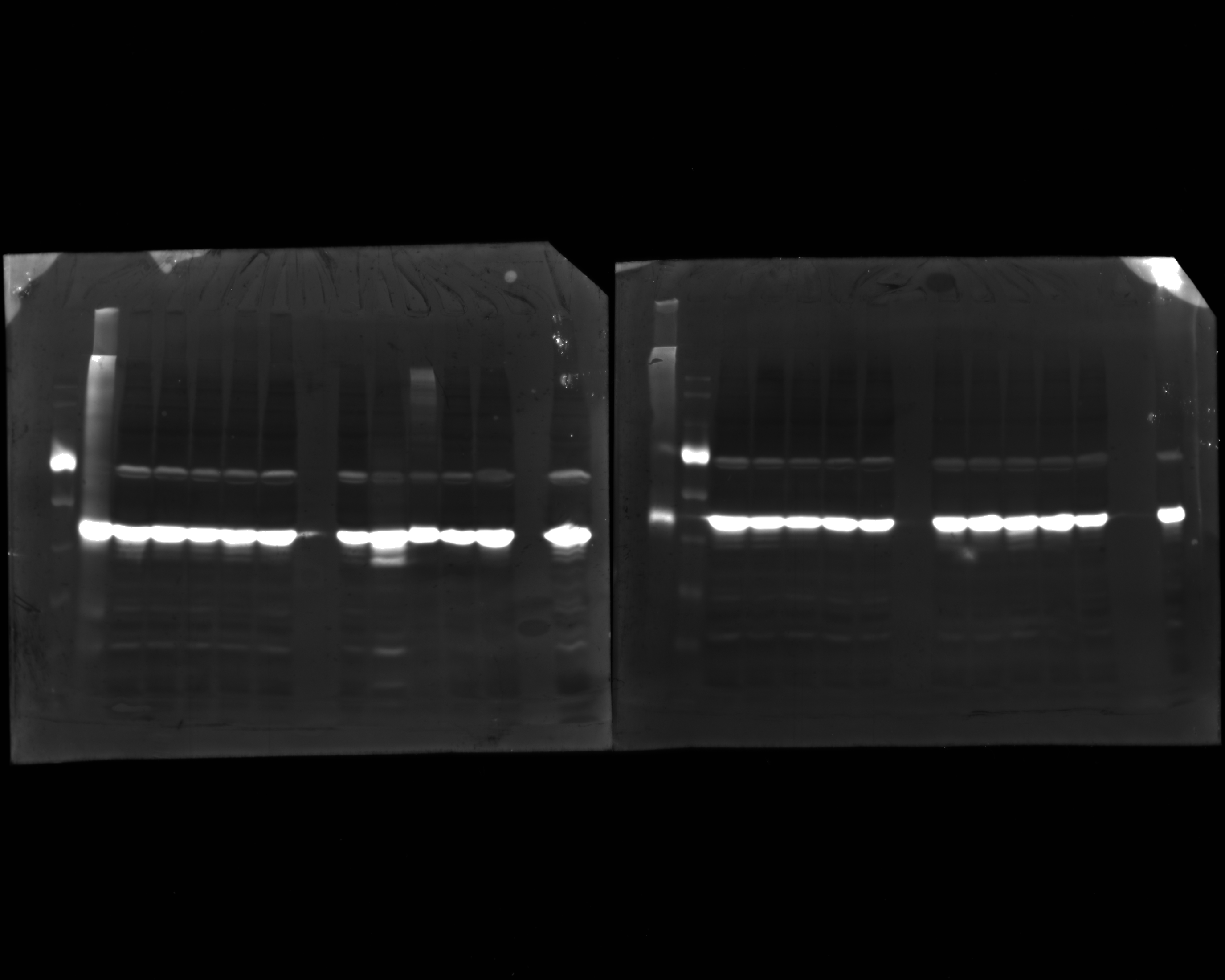

Supplement: Figure 4—figure supplement 1—source data 3. [file elife-87698-fig4-figsupp1-data3.zip › Figure 4-figure supplement 1-source data 3/Raw images/Figure 4-supplement 1C-TAX-1_CFAP70.tif]

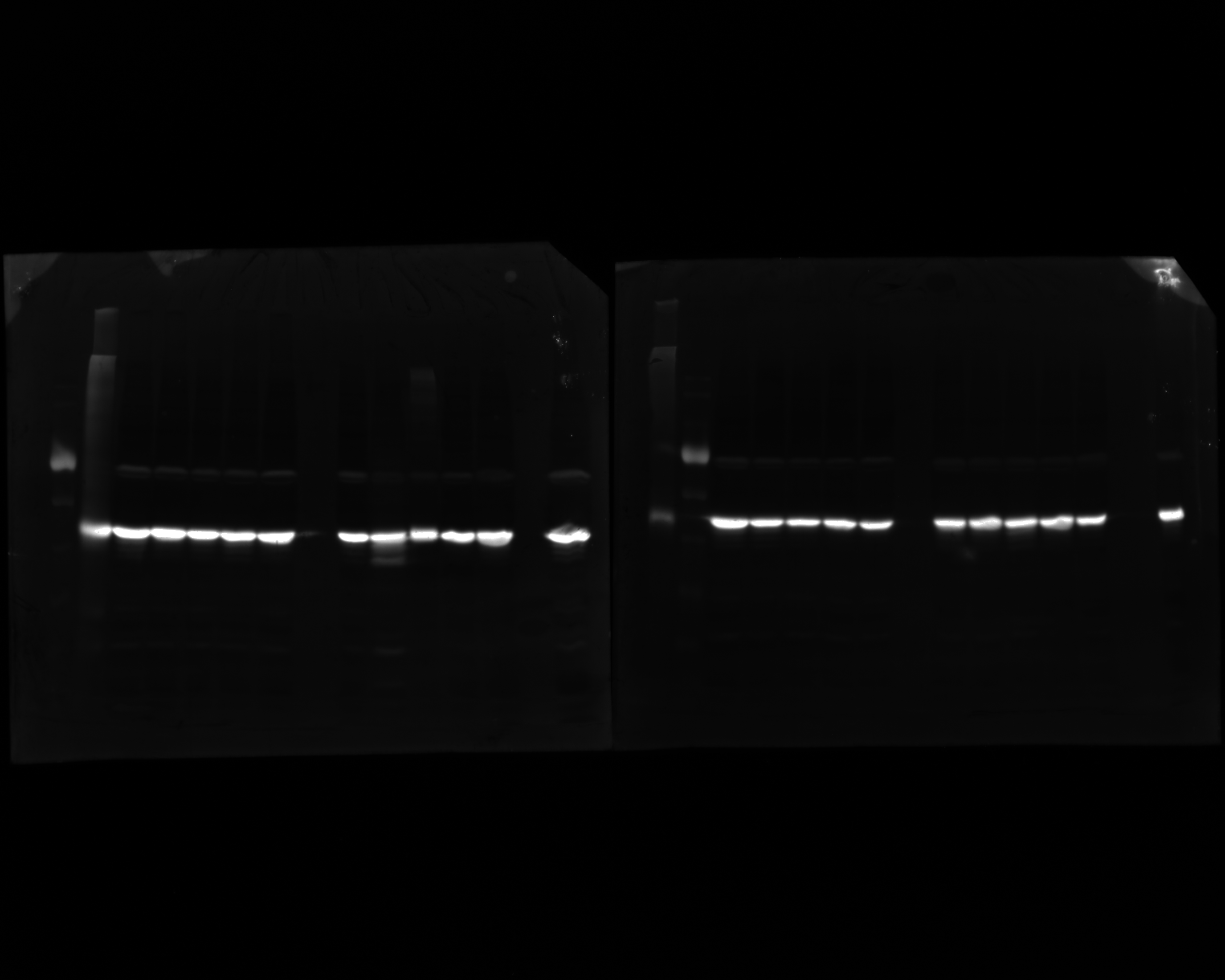

Supplement: Figure 4—figure supplement 1—source data 3. [file elife-87698-fig4-figsupp1-data3.zip › Figure 4-figure supplement 1-source data 3/Raw images/Figure 4-supplement 1C-ENOLASE_CFAP70.tif]

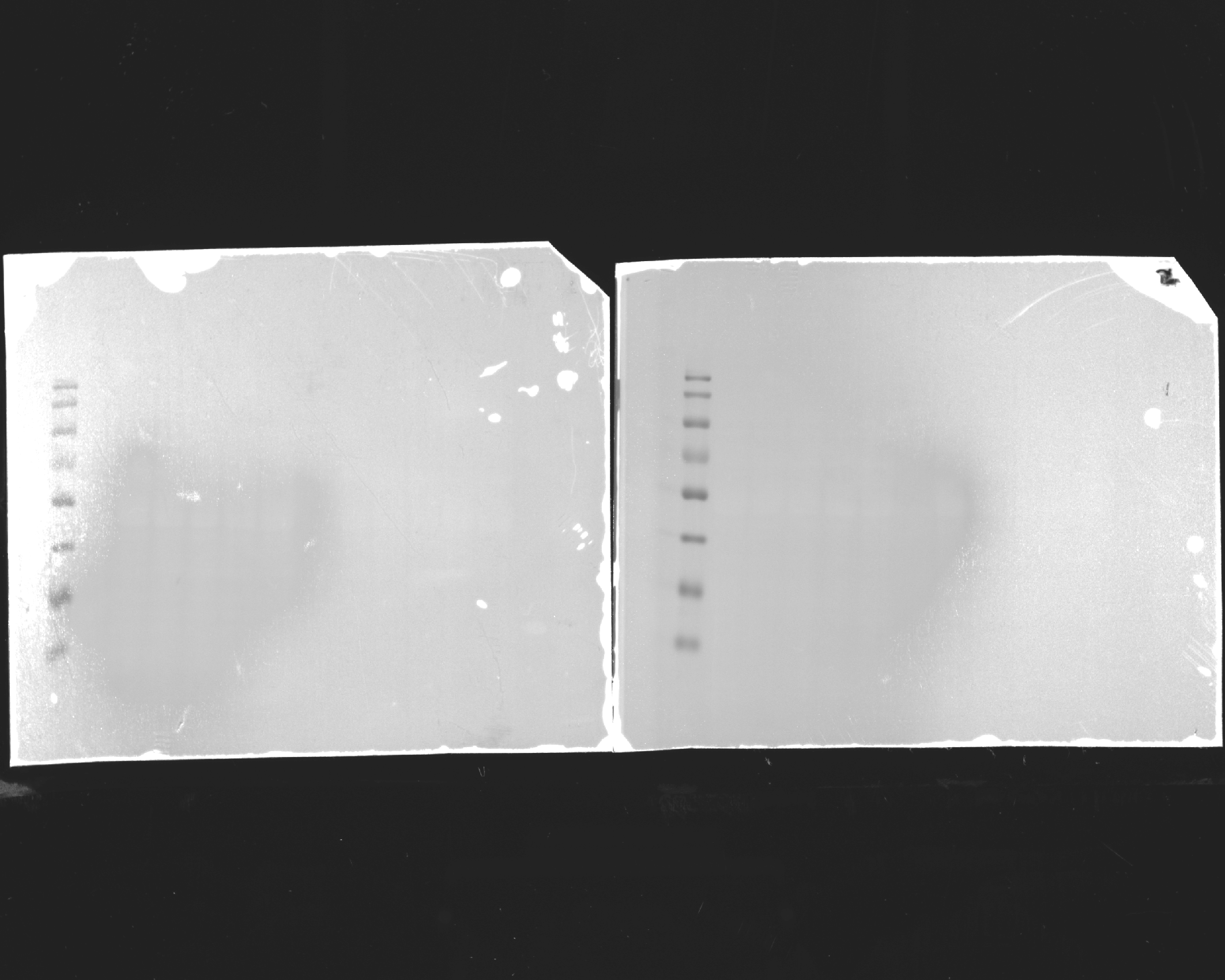

Supplement: Figure 4—figure supplement 1—source data 3. [file elife-87698-fig4-figsupp1-data3.zip › Figure 4-figure supplement 1-source data 3/Raw images/Figure 4-supplement 1C-RMW_CFAP70.tif]

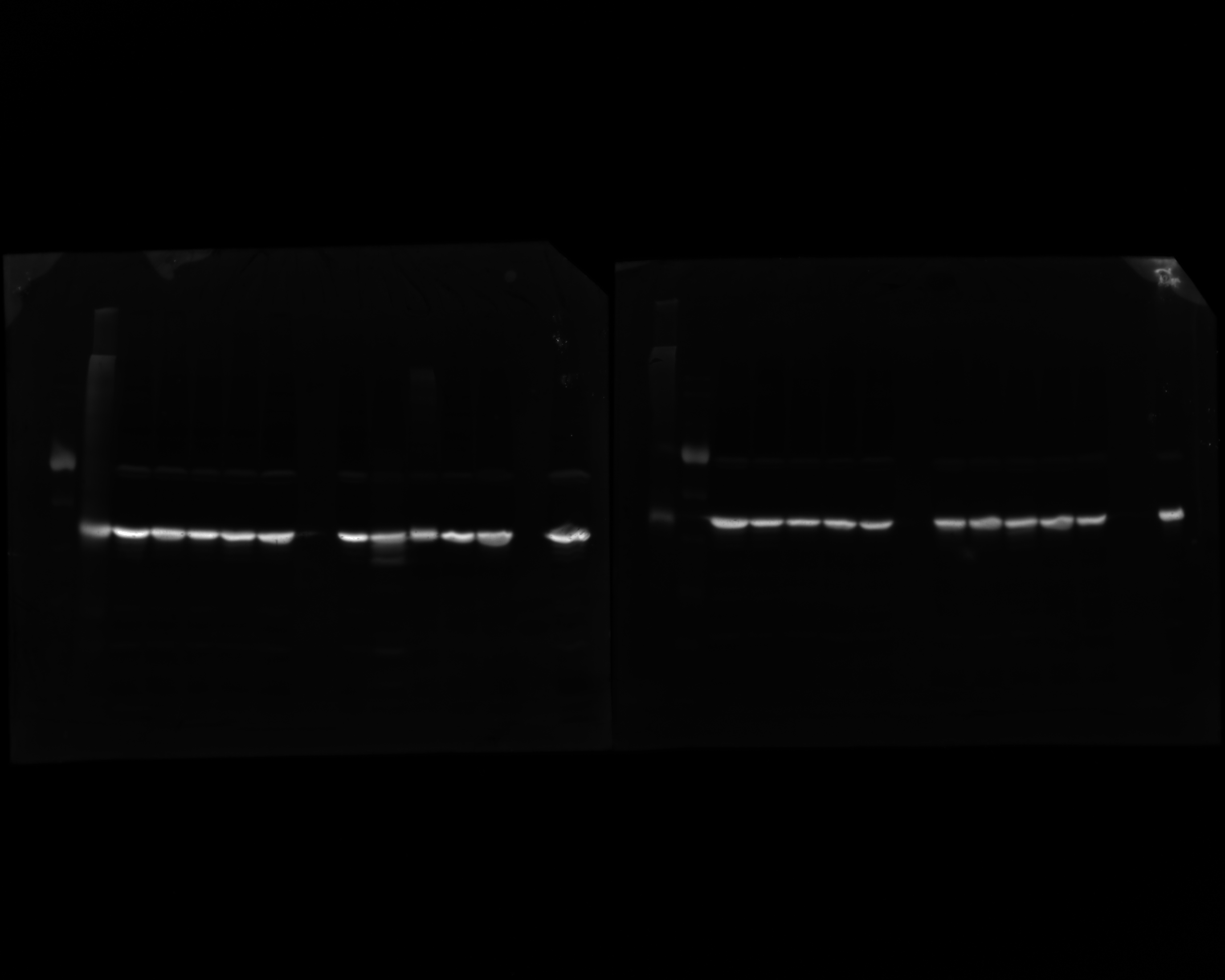

Supplement: Figure 4—figure supplement 1—source data 3. [file elife-87698-fig4-figsupp1-data3.zip › Figure 4-figure supplement 1-source data 3/Raw images/Figure 4-supplement 1C-ENOLASE_SPAG6.tif]

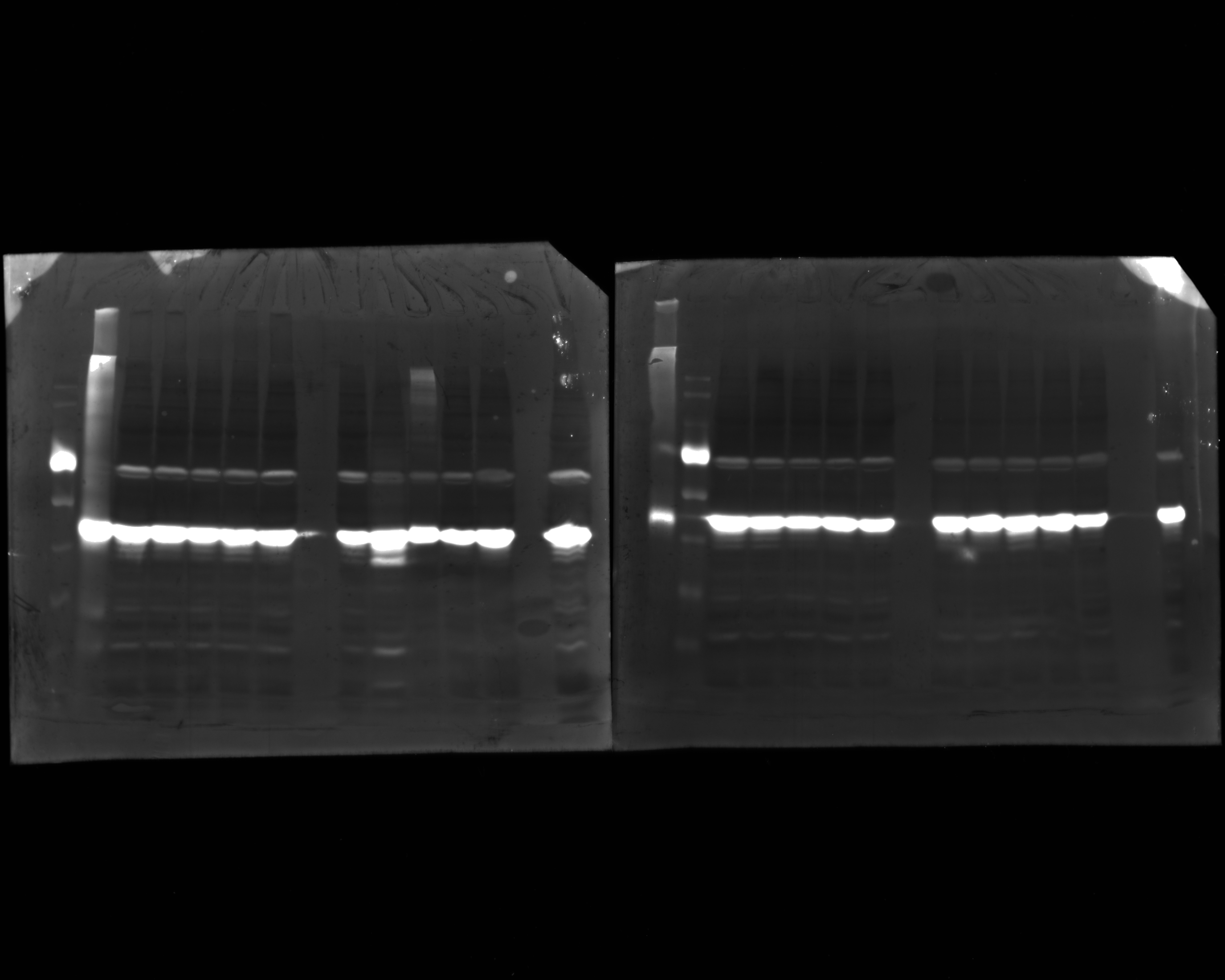

Supplement: Figure 4—figure supplement 1—source data 3. [file elife-87698-fig4-figsupp1-data3.zip › Figure 4-figure supplement 1-source data 3/Raw images/Figure 4-supplement 1C_TAX-1_SPAG6.tif]

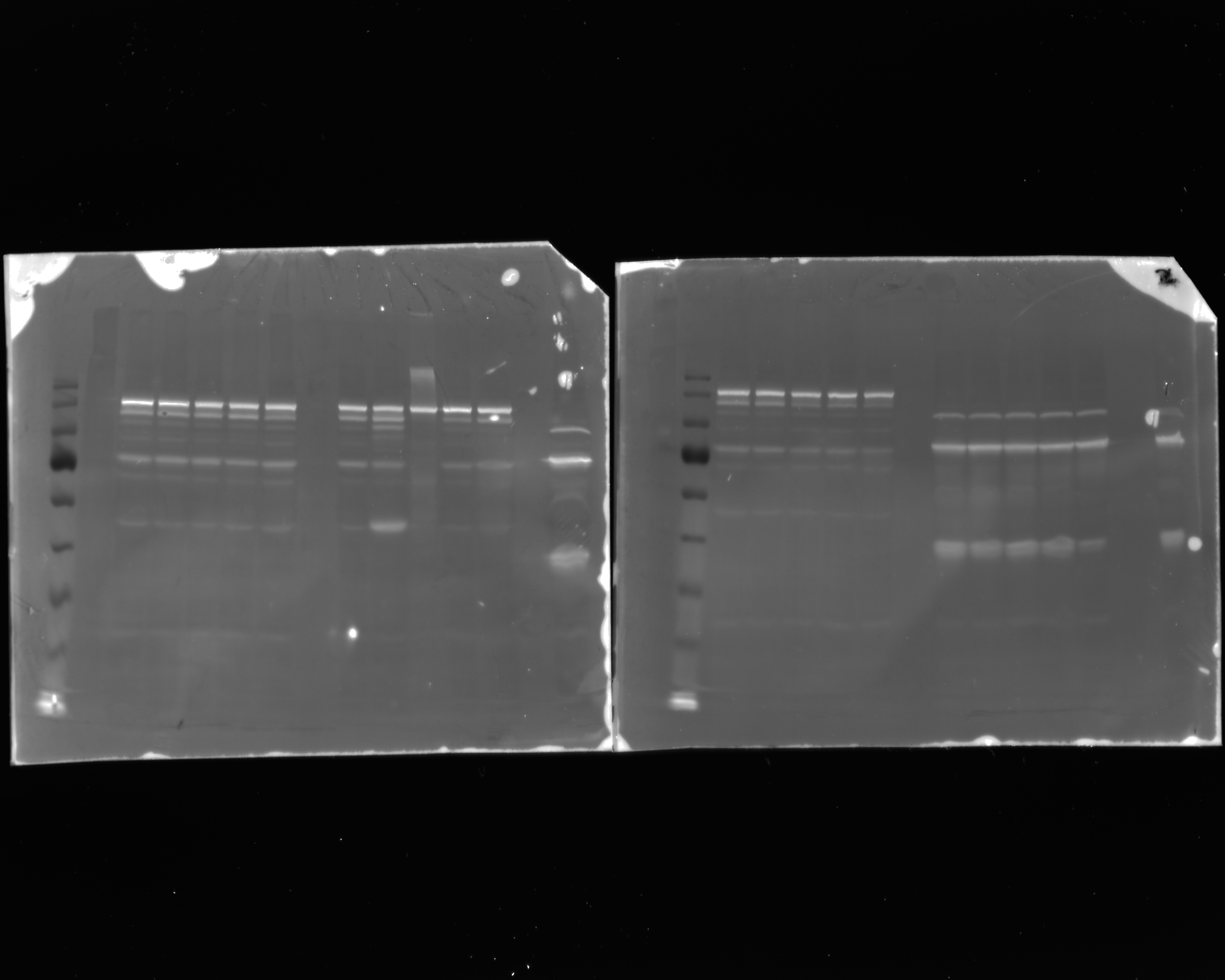

Supplement: Figure 4—figure supplement 1—source data 3. [file elife-87698-fig4-figsupp1-data3.zip › Figure 4-figure supplement 1-source data 3/Raw images/Figure 4-supplement 1C-TbCFAP70.tif]

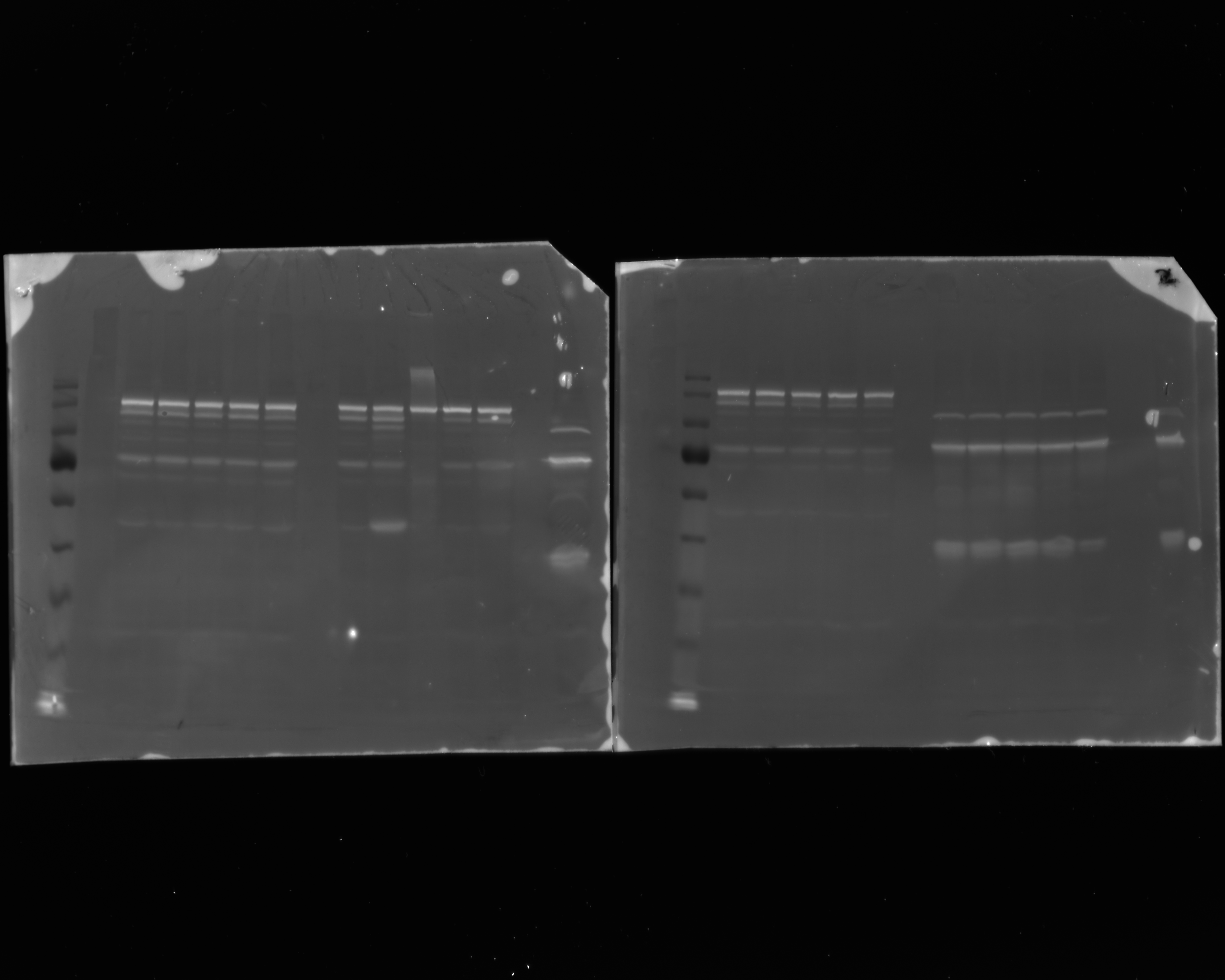

Supplement: Figure 4—figure supplement 1—source data 3. [file elife-87698-fig4-figsupp1-data3.zip › Figure 4-figure supplement 1-source data 3/Raw images/Figure 4-supplement 1C-SPAG6.tif]

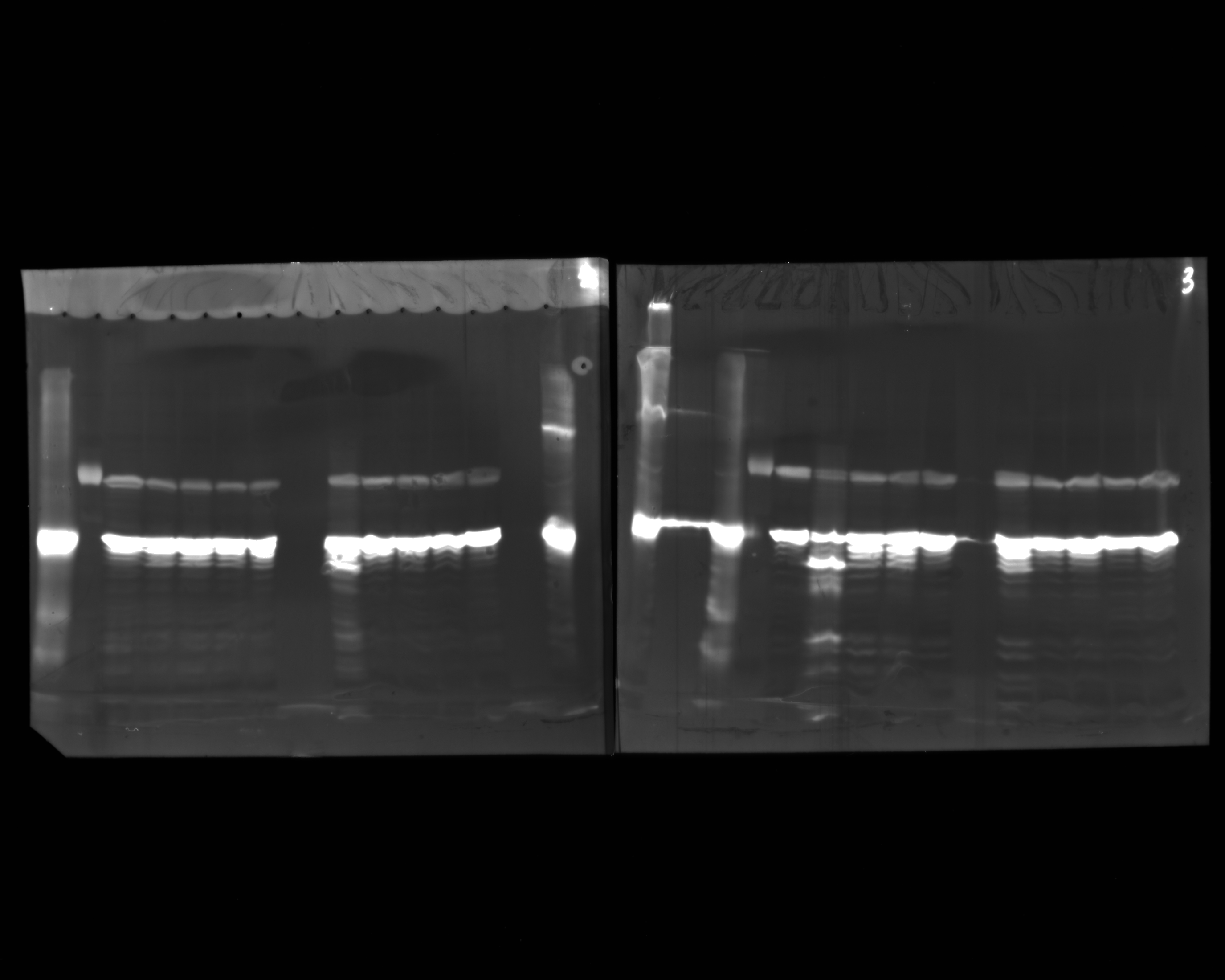

Supplement: Figure 4—figure supplement 1—source data 3. [file elife-87698-fig4-figsupp1-data3.zip › Figure 4-figure supplement 1-source data 3/Raw images/Figure 4-supplement 1C-TAX-1_WDR66.tif]

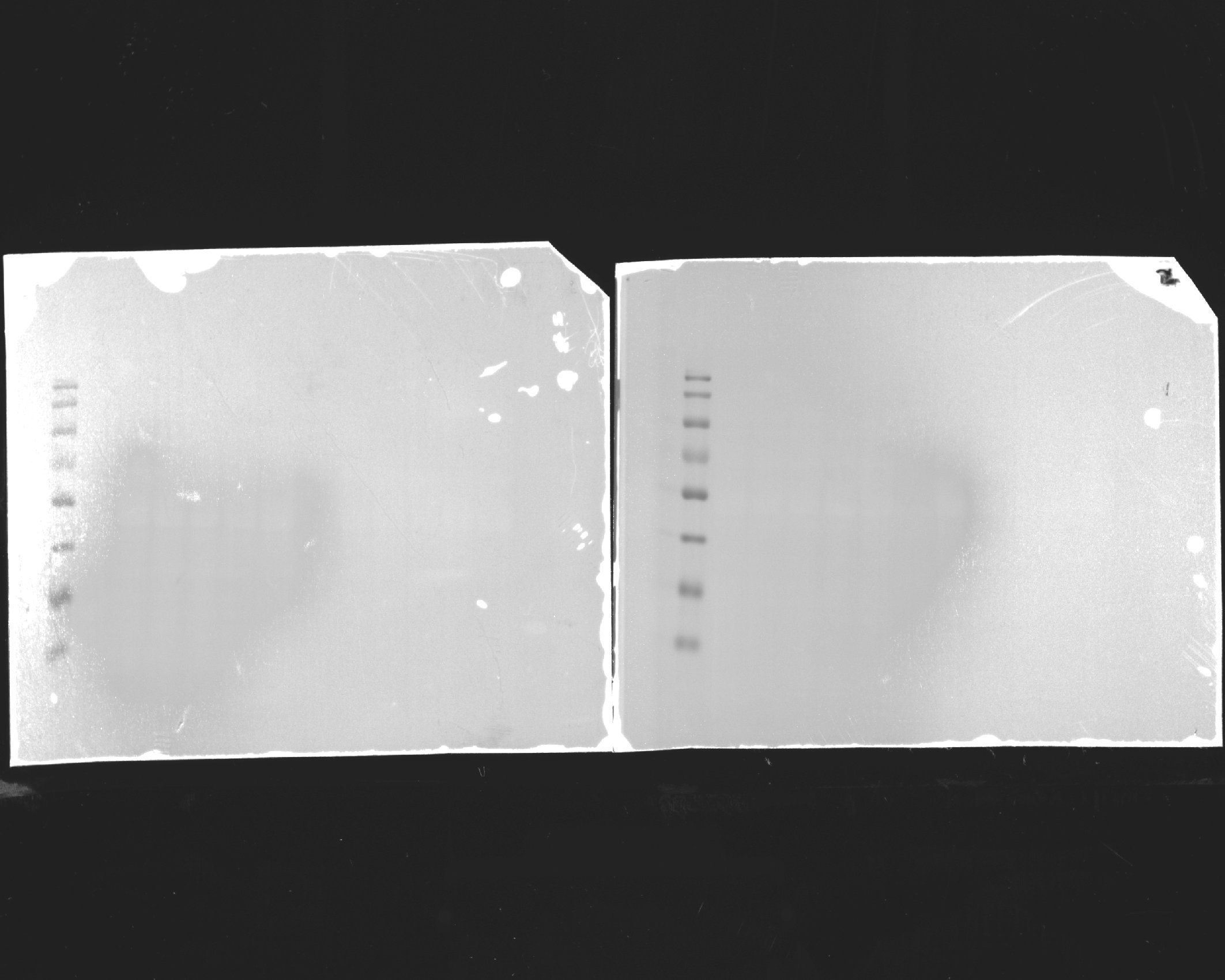

Supplement: Figure 4—figure supplement 1—source data 3. [file elife-87698-fig4-figsupp1-data3.zip › Figure 4-figure supplement 1-source data 3/Raw images/Figure 4-supplement 1C-MW-SPAG6.tif]

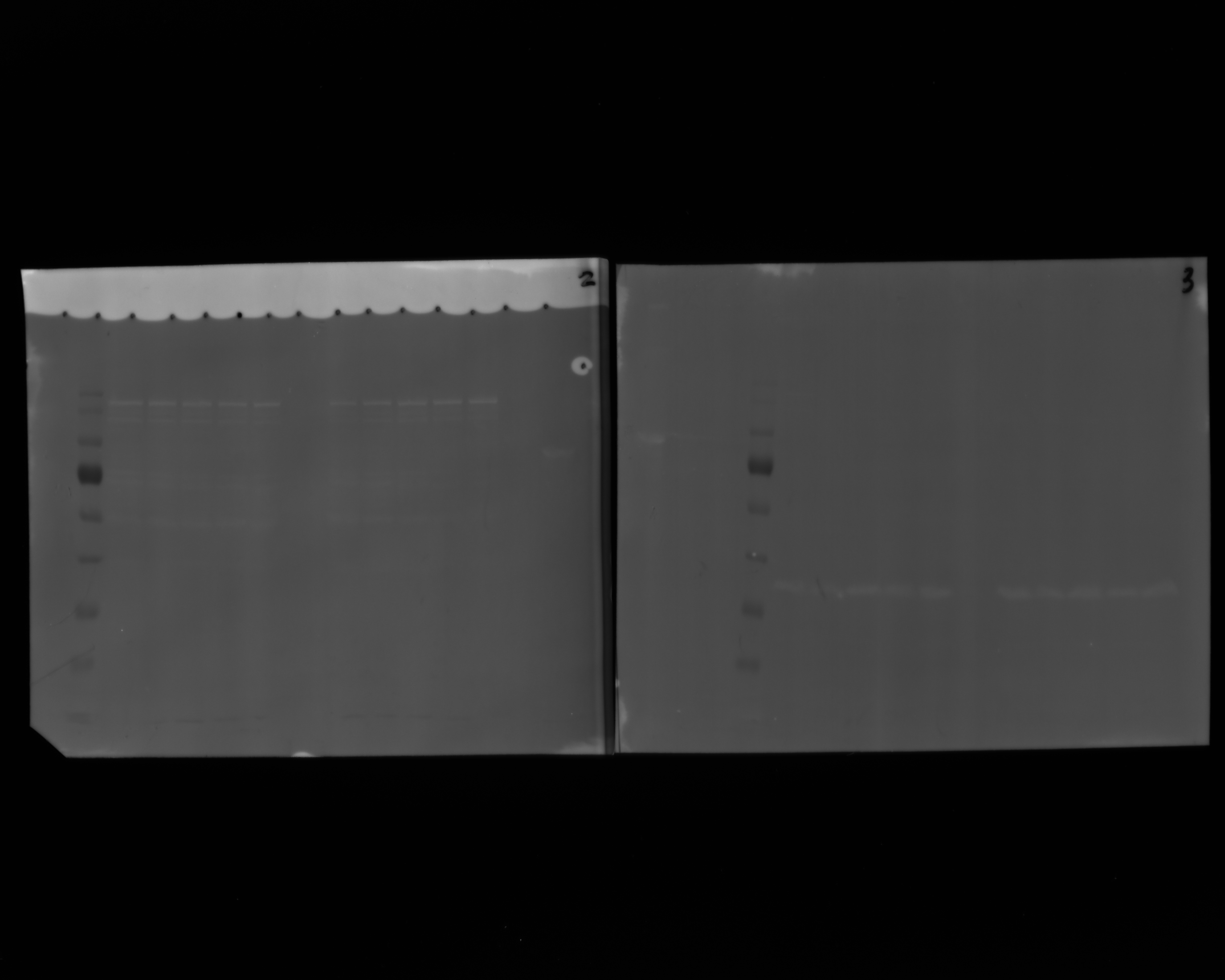

Supplement: Figure 4—figure supplement 1—source data 3. [file elife-87698-fig4-figsupp1-data3.zip › Figure 4-figure supplement 1-source data 3/Raw images/Figure 4-supplement 1C-WDR66.tif]

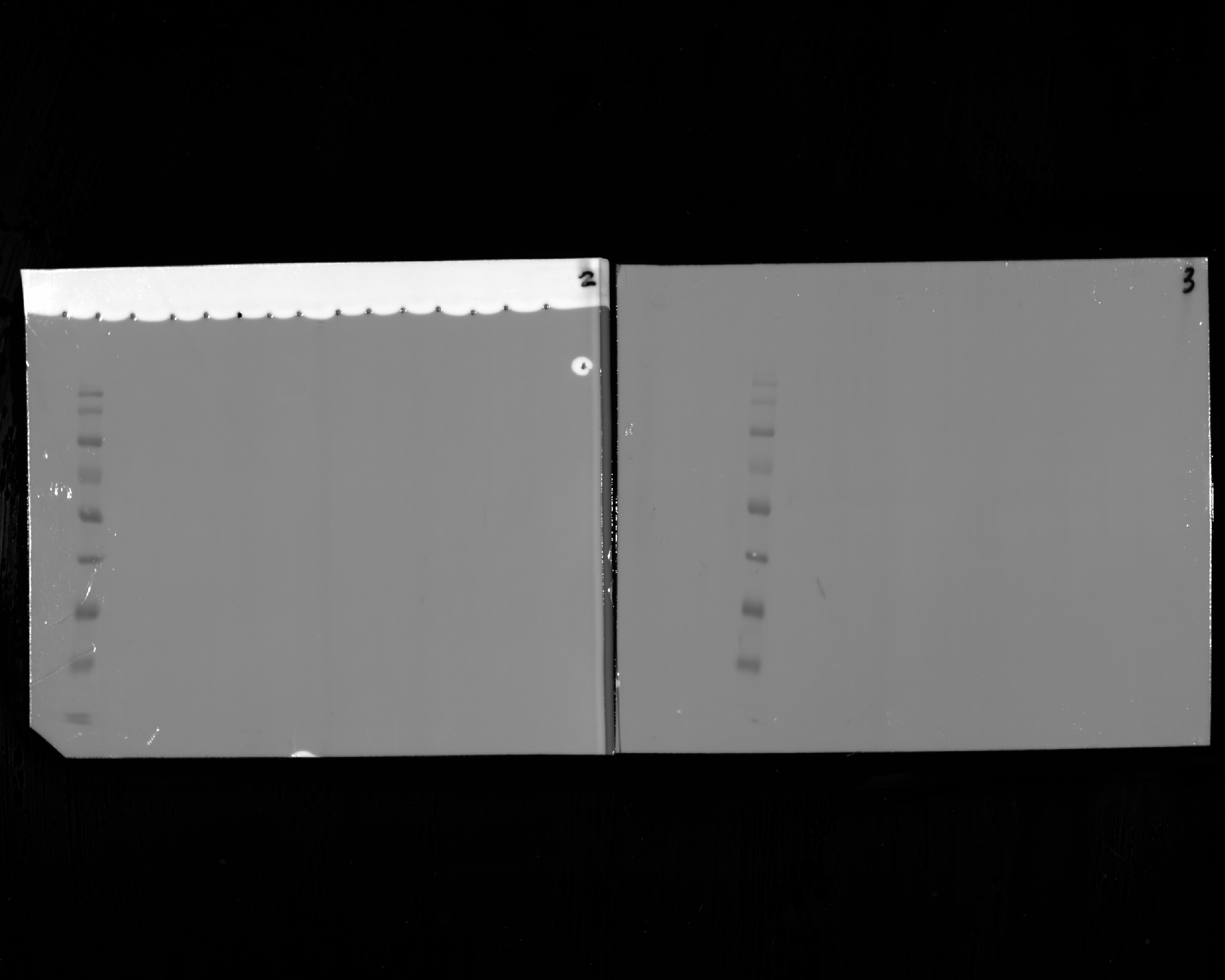

Supplement: Figure 4—figure supplement 1—source data 3. [file elife-87698-fig4-figsupp1-data3.zip › Figure 4-figure supplement 1-source data 3/Raw images/Figure 4-supplement 1C-MW_WDR66.tif]

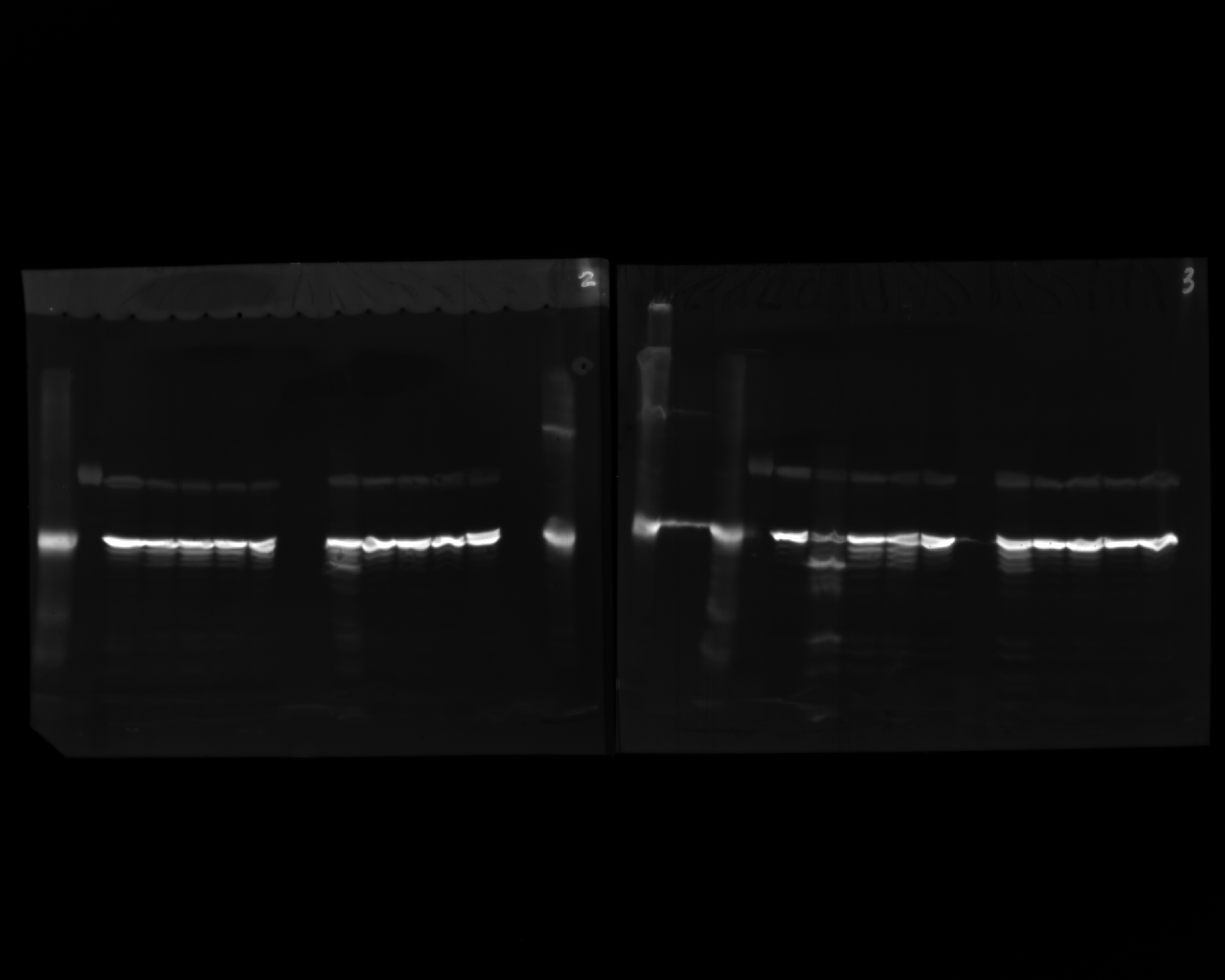

Supplement: Figure 4—figure supplement 1—source data 3. [file elife-87698-fig4-figsupp1-data3.zip › Figure 4-figure supplement 1-source data 3/Raw images/Figure 4-supplement 1C_ENOLASE_WDR66.tif]

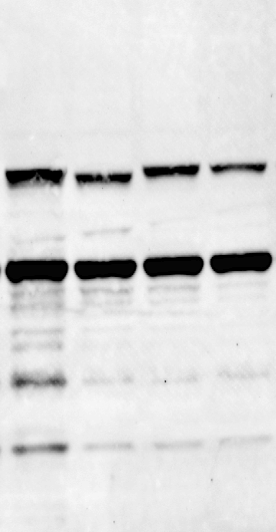

Supplement: Figure 5—source data 1. [file elife-87698-fig5-data1.zip › Figure 5-source data 1/Figure 5A-TTC29Ty1-TAX1HA-RNAiTAx1-antiHA antiEnolase.tif]

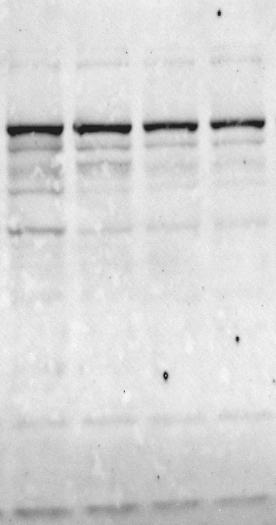

Supplement: Figure 5—source data 1. [file elife-87698-fig5-data1.zip › Figure 5-source data 1/Figure 5A-TTC29Ty1-TAX1HA-RNAiTAx1-antiTY1 antiEnolase.tif]

**A**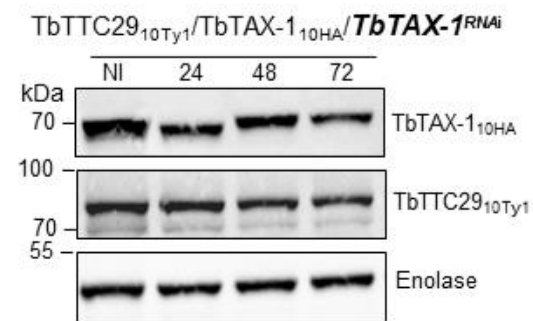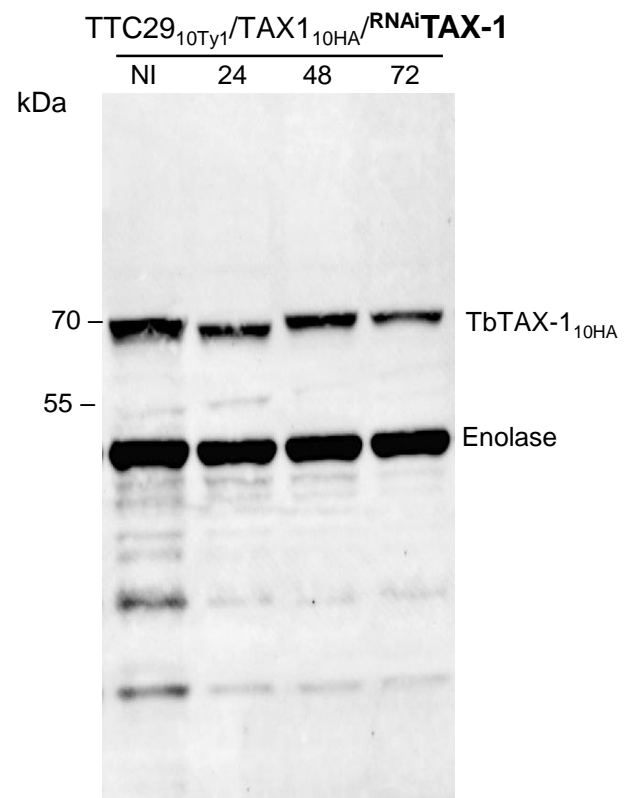

Anti-HA + anti-Enolase

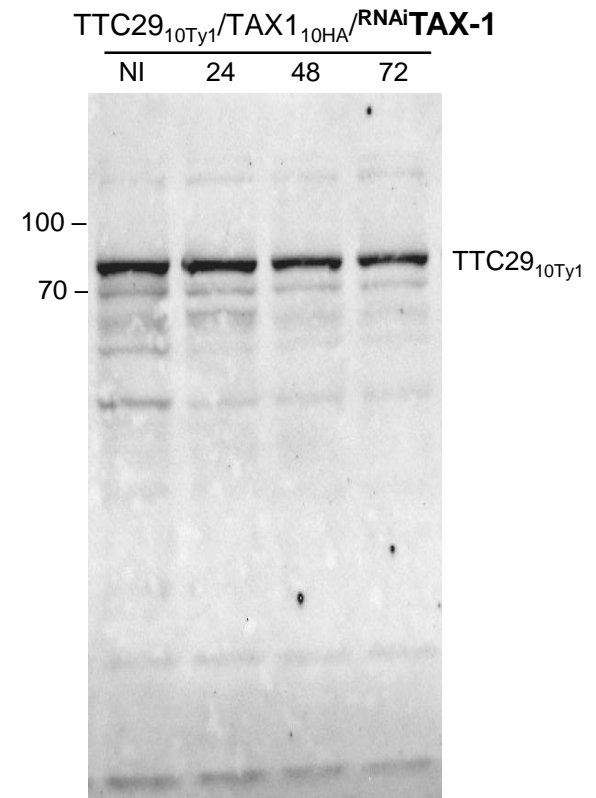

Anti-Ty1 + anti-Enolase

Supplement: Figure 5—source data 1. [file elife-87698-fig5-data1.zip › Figure 5-source data 1/Figure 5A-uncropped blots.pdf]

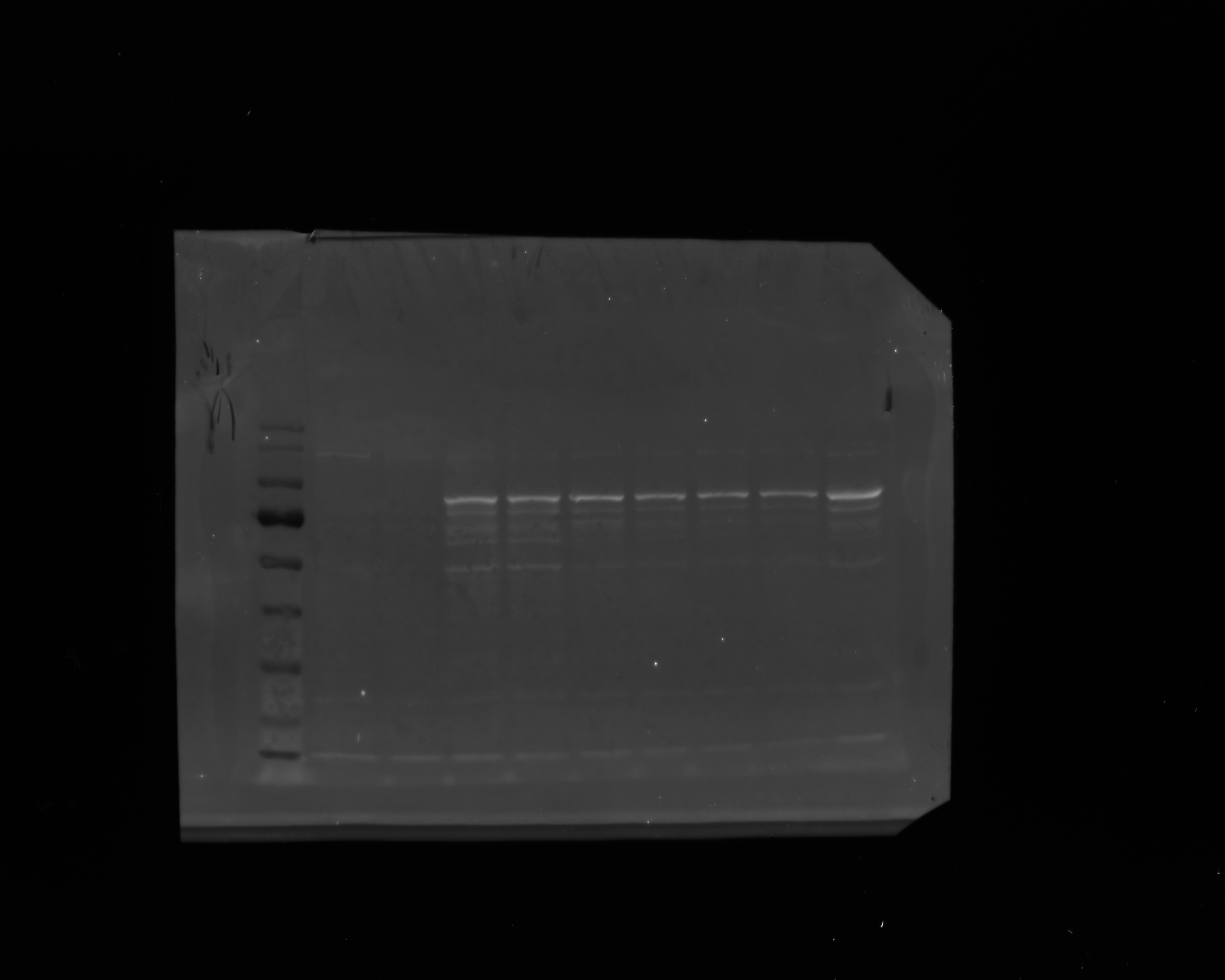

Supplement: Figure 5—source data 1. [file elife-87698-fig5-data1.zip › Figure 5-source data 1/Raw images/Figure 5A-TTC29.tif]

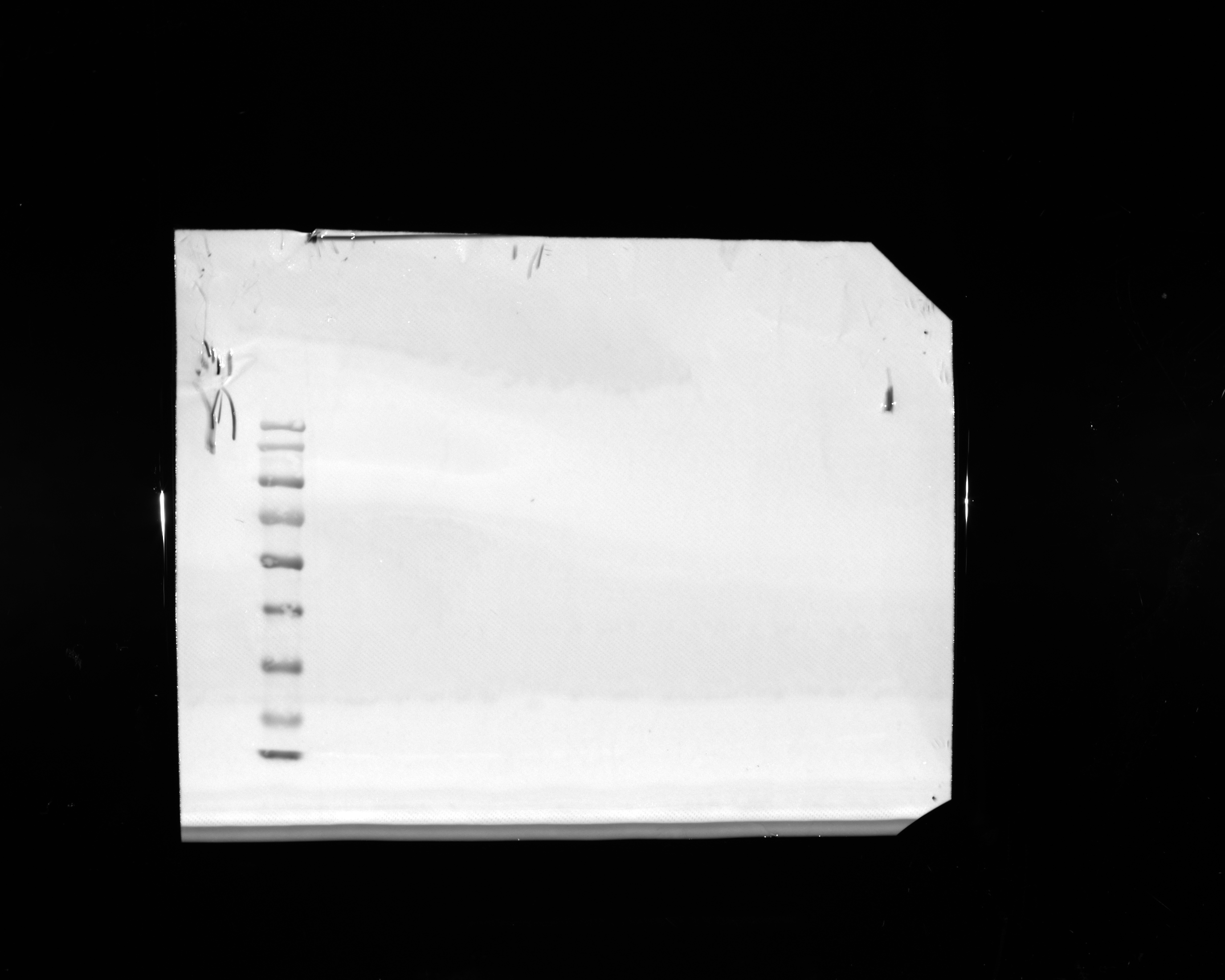

Supplement: Figure 5—source data 1. [file elife-87698-fig5-data1.zip › Figure 5-source data 1/Raw images/Figure 5A-MW.tif]

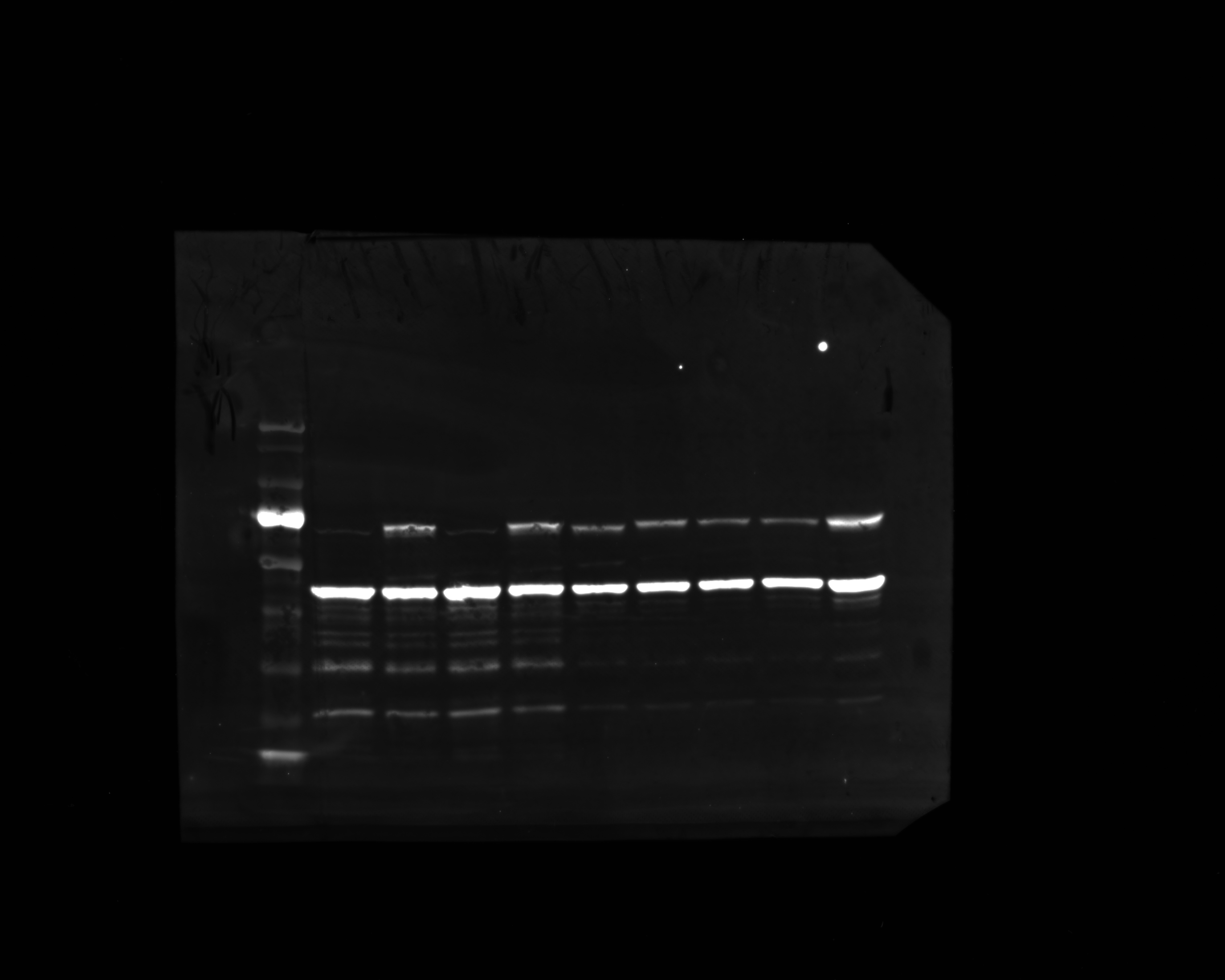

Supplement: Figure 5—source data 1. [file elife-87698-fig5-data1.zip › Figure 5-source data 1/Raw images/Figure 5A-TAX1 Enolase.tif]

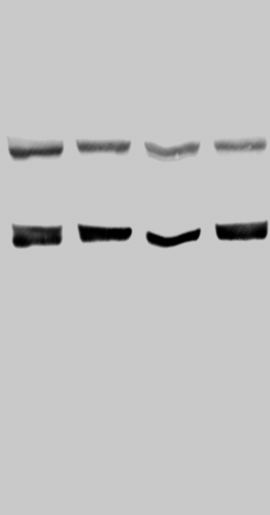

Supplement: Figure 5—source data 2. [file elife-87698-fig5-data2.zip › Figure 5-source data 2/Figure 5B-TTC29Ty1-TAX1HA-RNAiTTC29-antiHA antiEnolase.tif]

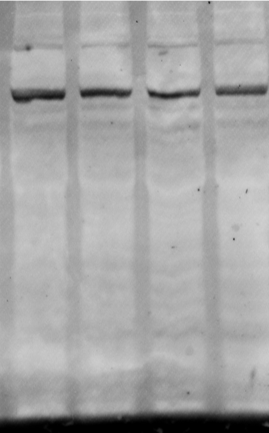

Supplement: Figure 5—source data 2. [file elife-87698-fig5-data2.zip › Figure 5-source data 2/Figure 5B-TTC29Ty1-TAX1HA-RNAiTTC29-antiTY1.tif]

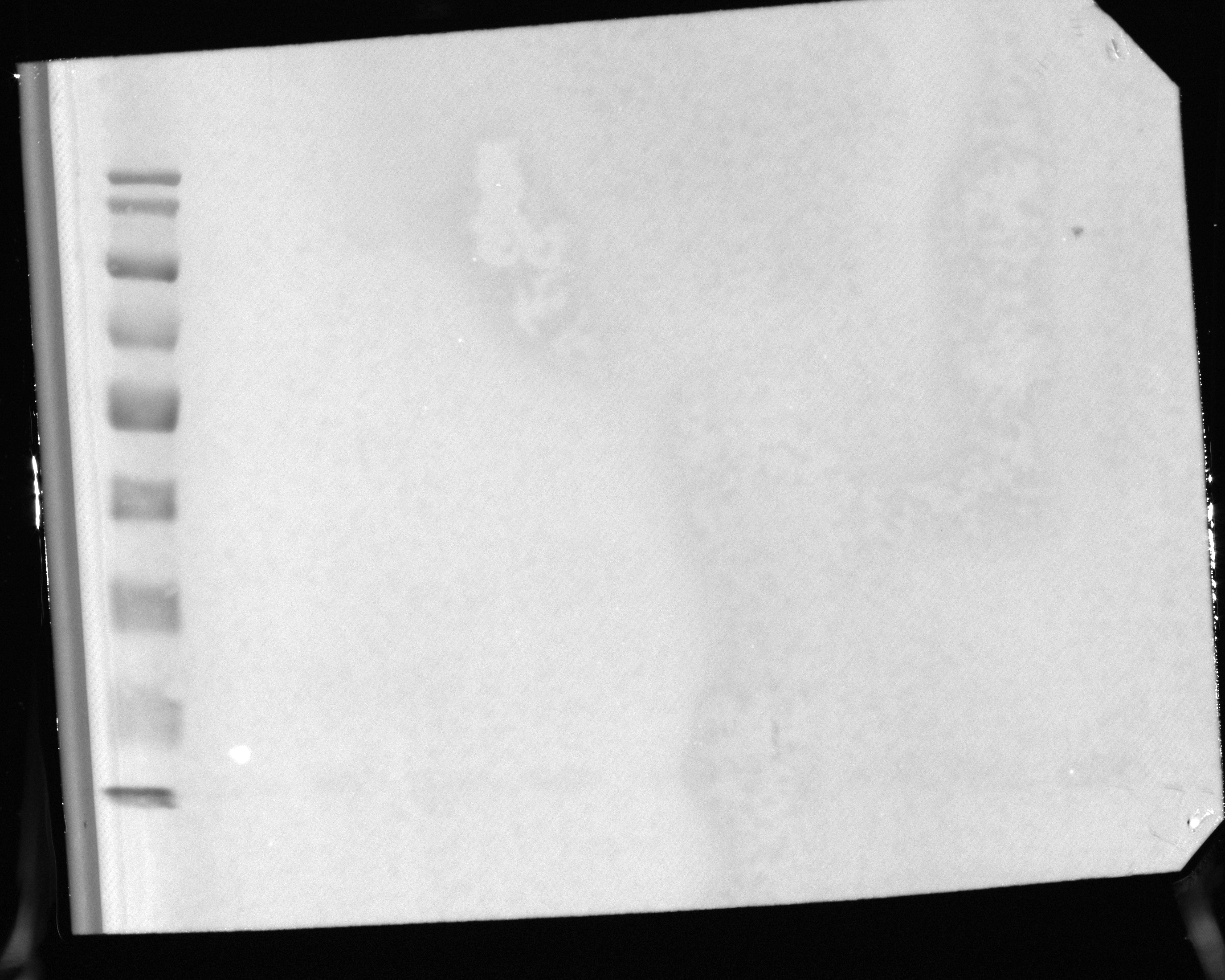

Supplement: Figure 5—source data 2. [file elife-87698-fig5-data2.zip › Figure 5-source data 2/Raw images/Figure 5B-MW.tif]

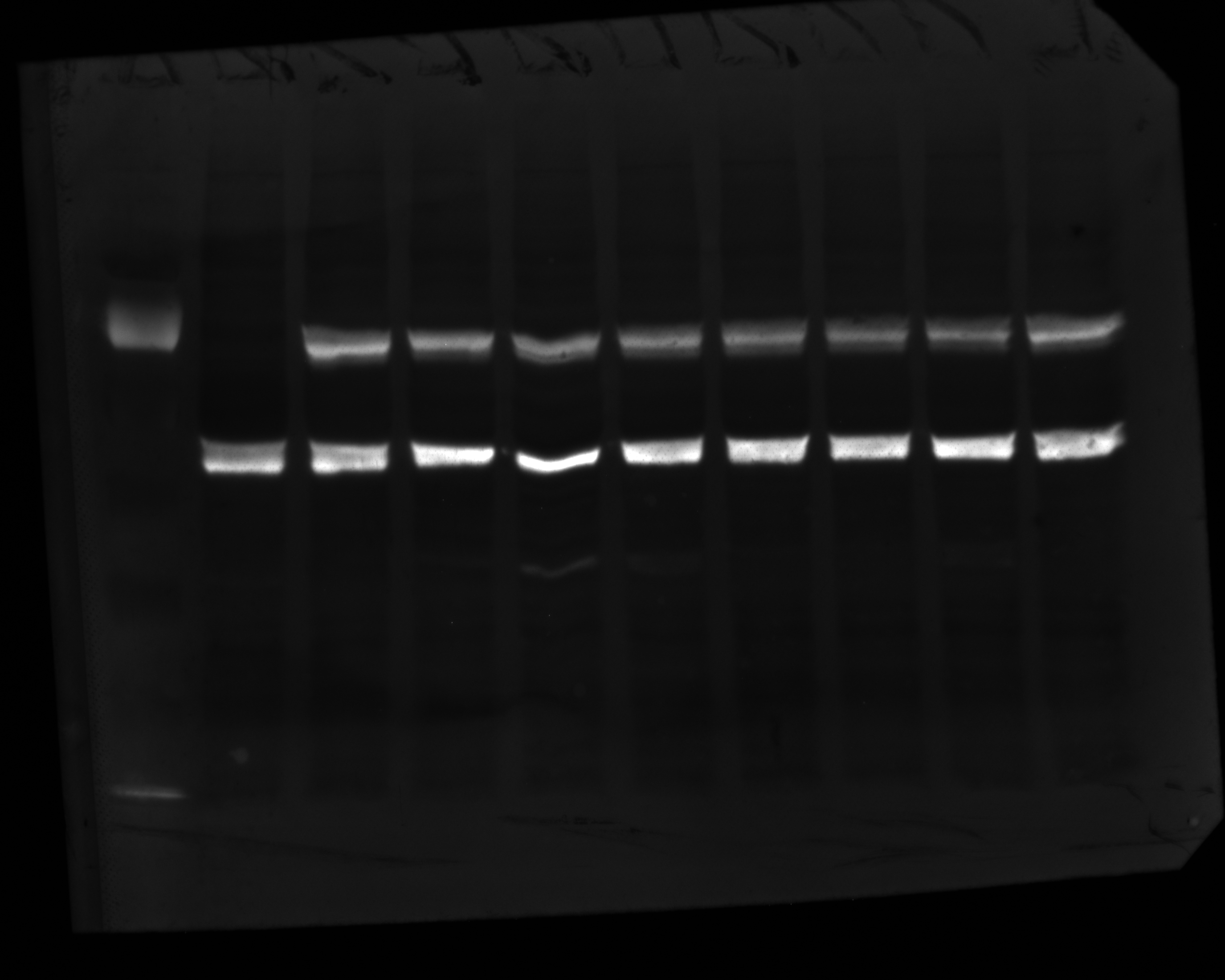

Supplement: Figure 5—source data 2. [file elife-87698-fig5-data2.zip › Figure 5-source data 2/Raw images/Figure 5B-TAX1 Enolase.tif]

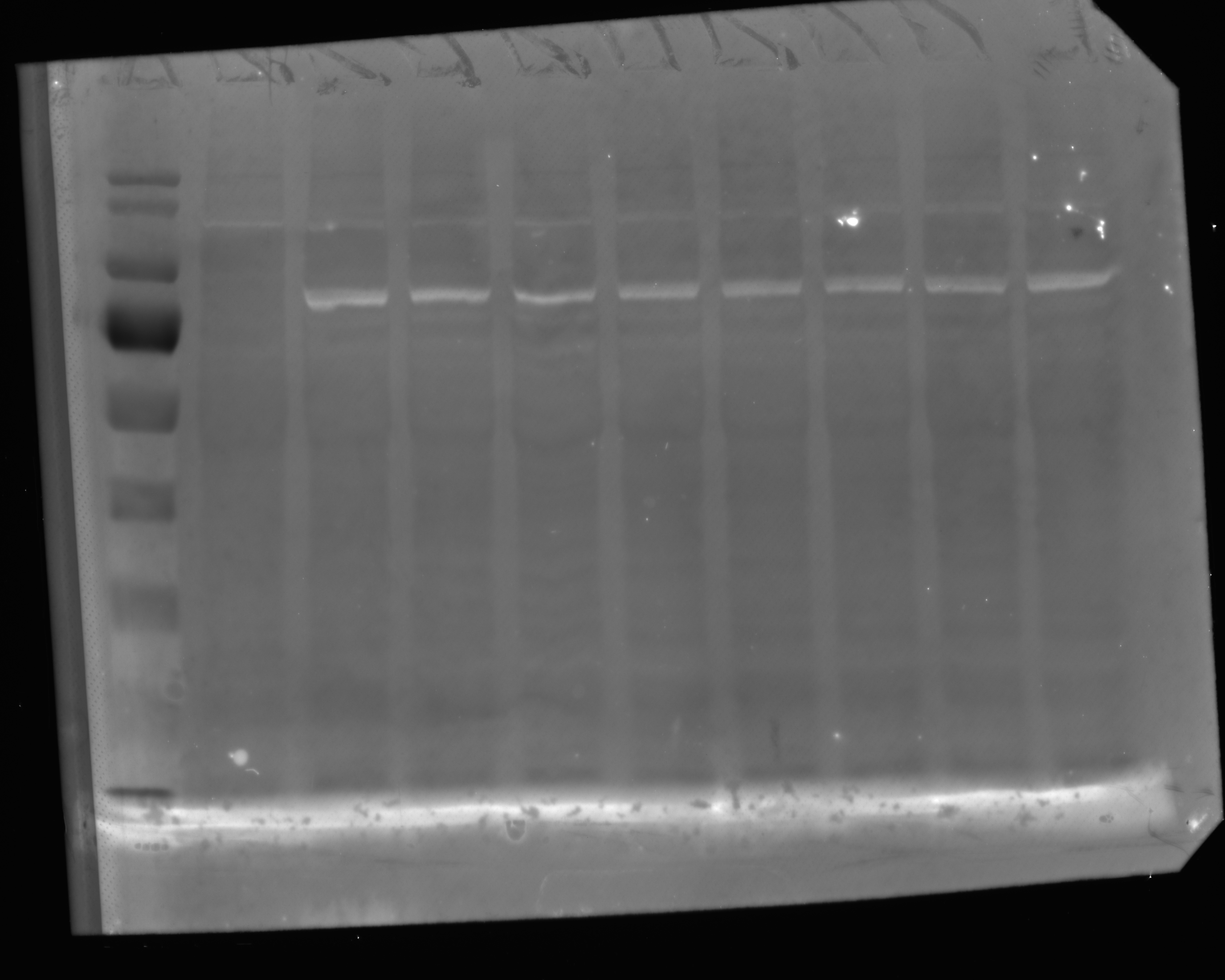

Supplement: Figure 5—source data 2. [file elife-87698-fig5-data2.zip › Figure 5-source data 2/Raw images/Figure 5B-TTC29.tif]

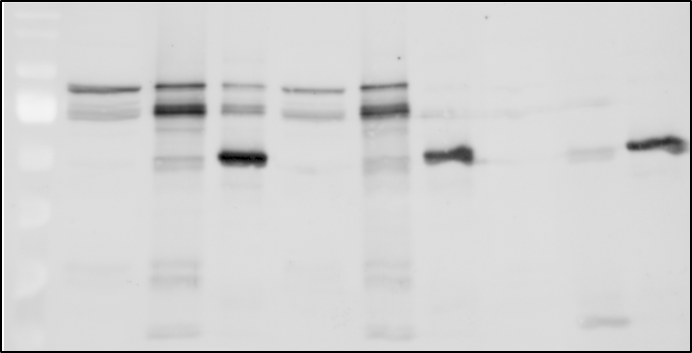

Supplement: Figure 5—source data 3. [file elife-87698-fig5-data3.zip › Figure 5-source data 3/Figure 5E-antiTY1.tif]

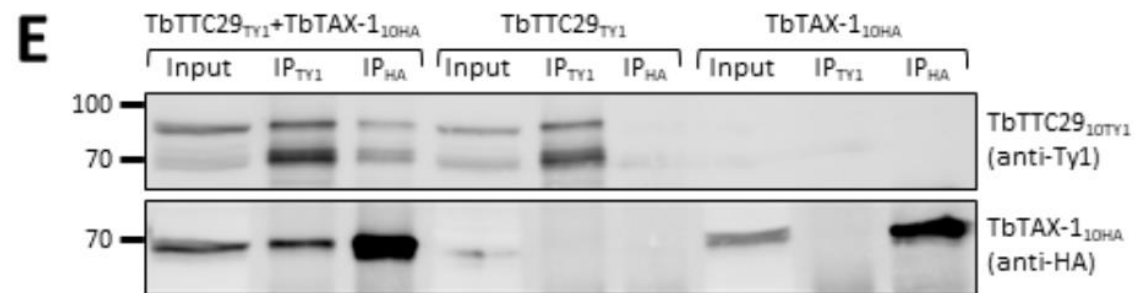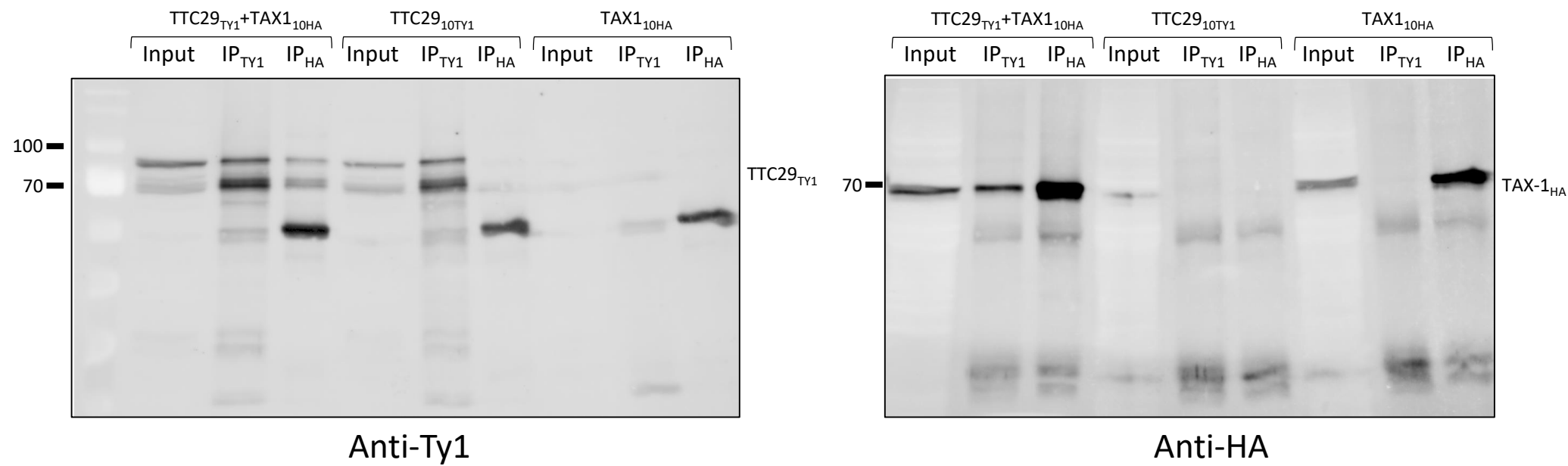

Supplement: Figure 5—source data 3. [file elife-87698-fig5-data3.zip › Figure 5-source data 3/Figure 5E-uncropped blots.pdf]

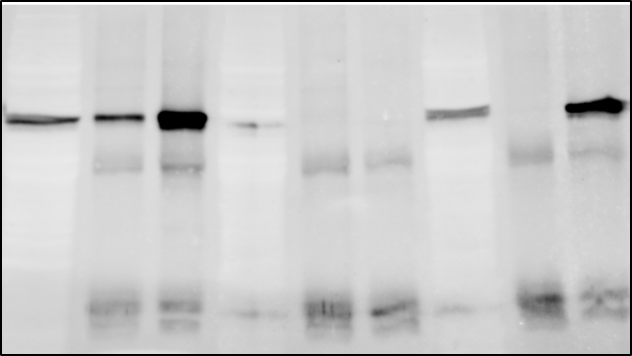

Supplement: Figure 5—source data 3. [file elife-87698-fig5-data3.zip › Figure 5-source data 3/Figure 5E-antiHA.tif]

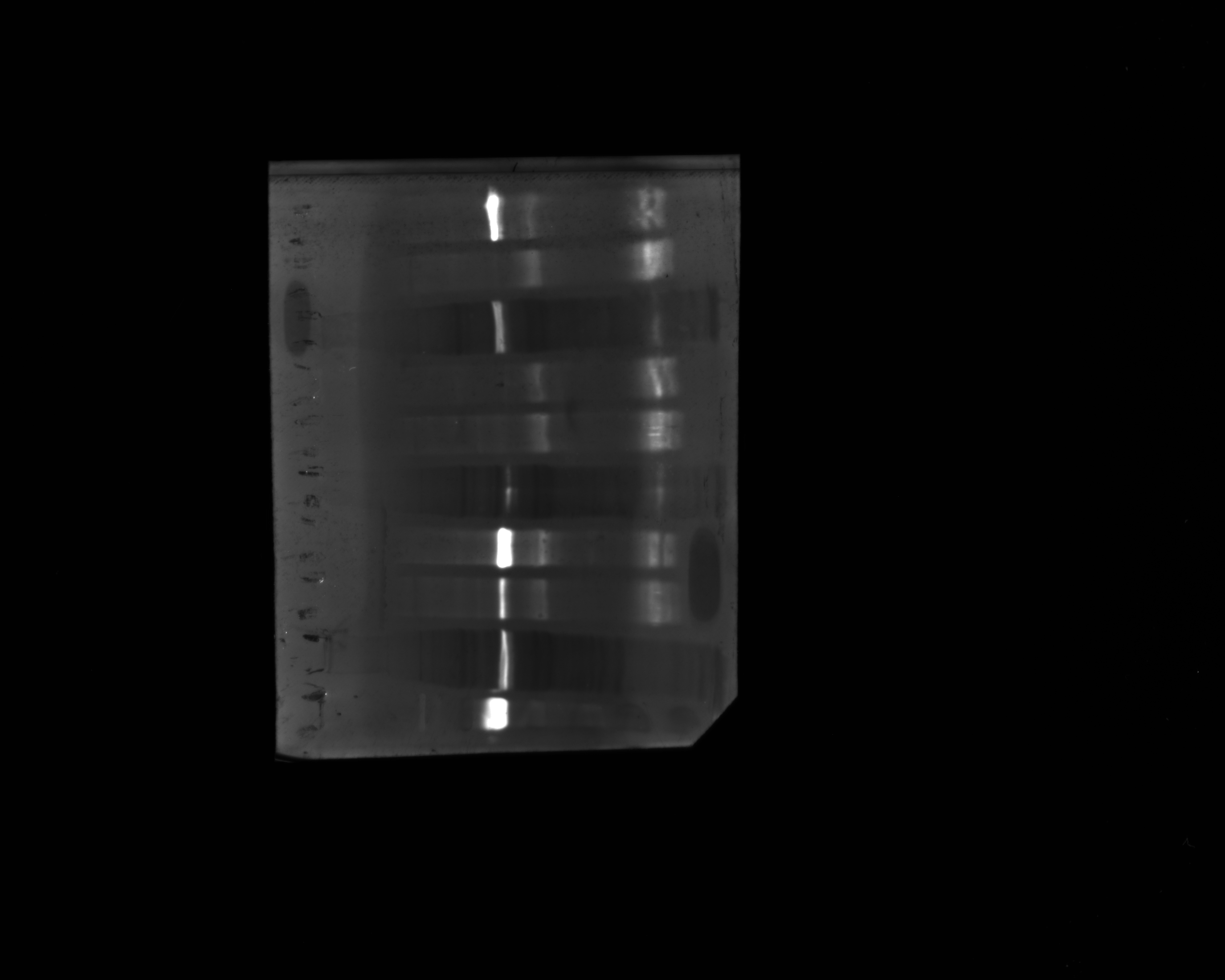

Supplement: Figure 5—source data 3. [file elife-87698-fig5-data3.zip › Figure 5-source data 3/Raw images/Figure 5E-TAX1.tif]

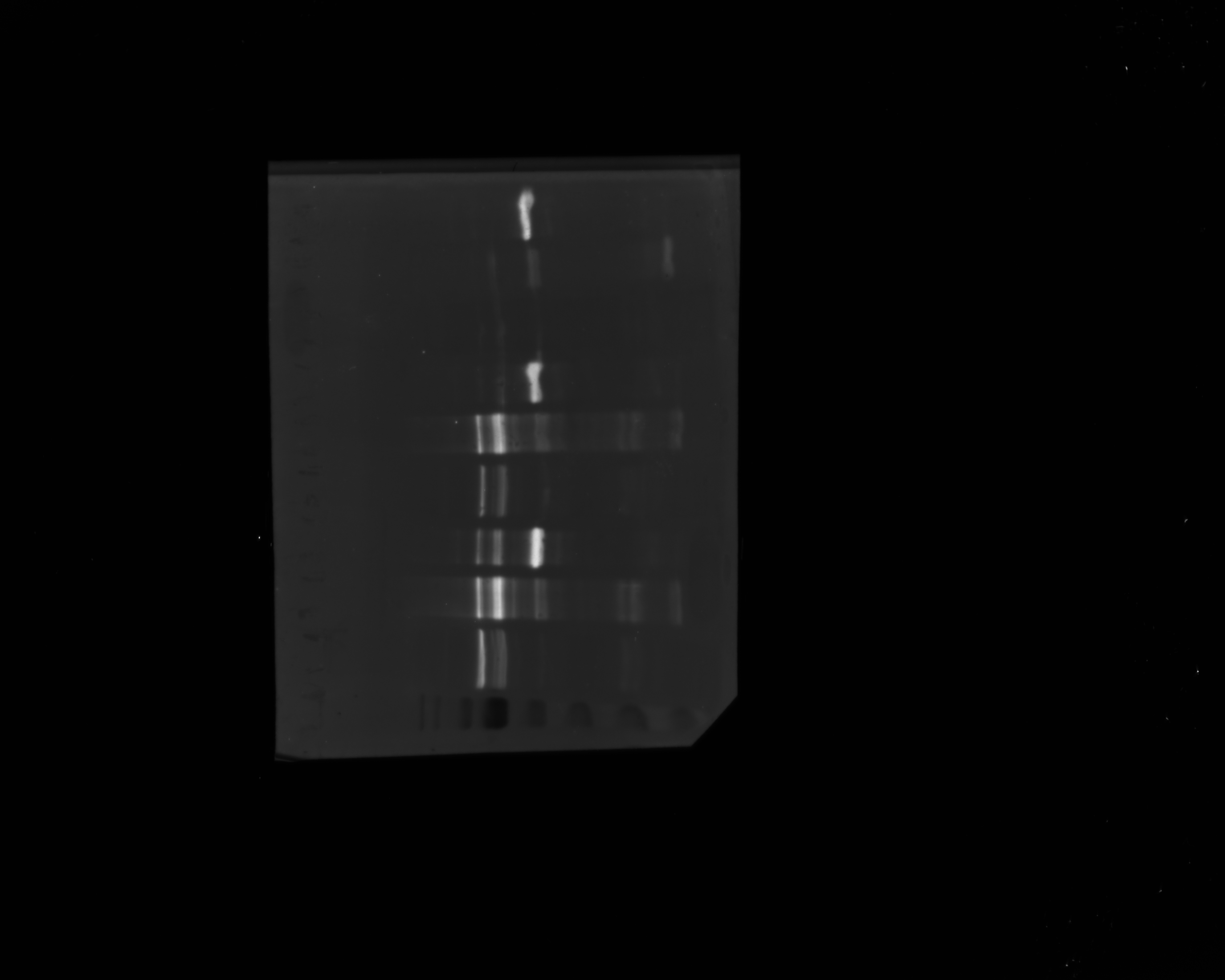

Supplement: Figure 5—source data 3. [file elife-87698-fig5-data3.zip › Figure 5-source data 3/Raw images/Figure 5E-TTC29.tif]
