# Supplementary material for: Novel axonemal protein ZMYND12 interacts with TTC29 and DNAH1, and is required for male fertility and flagellum function
Source: eLife. 2023 Nov 7;12:RP87698. doi: 10.7554/eLife.87698 (PMC10629824; doi:10.7554/eLife.87698)
Supplement: Figure 5—source data 2. [file elife-87698-fig5-data2.zip › Figure 5-source data 2/Figure 5B-uncropped blots.pdf]

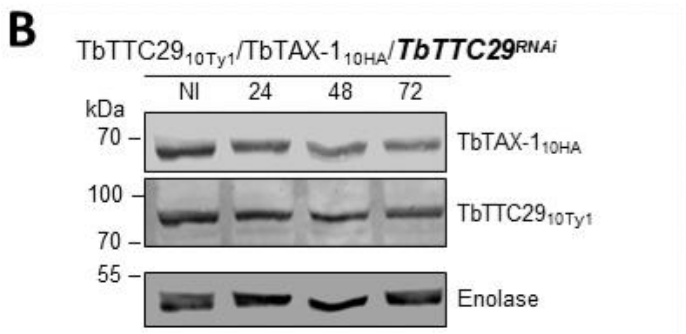
B
TbTTC29$_{10Ty1}$/TbTAX-1$_{10HA}$/***TbTTC29***^{RNAi}
NI 24 48 72
kDa
70 –
TbTAX-1$_{10HA}$
100 –
TbTTC29$_{10Ty1}$
70 –
55 –
Enolase

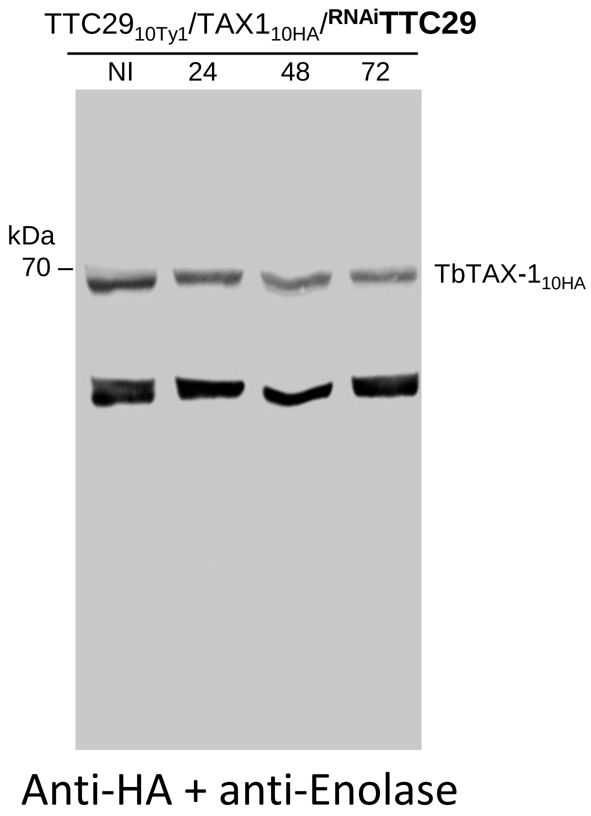
TTC29$_{10Ty1}$/TAX1$_{10HA}$/^{RNAi}**TTC29**
NI 24 48 72
kDa
70 –
TbTAX-1$_{10HA}$
Anti-HA + anti-Enolase

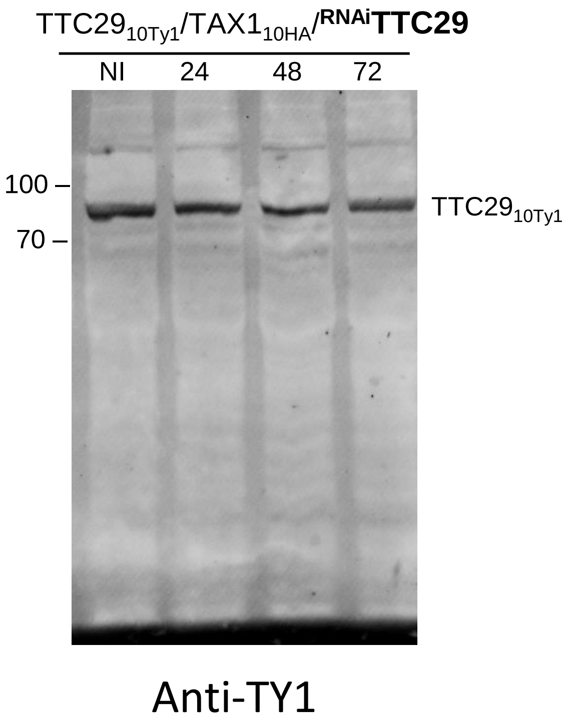
TTC29$_{10Ty1}$/TAX1$_{10HA}$/^{RNAi}**TTC29**
NI 24 48 72
100 –
70 –
TTC29$_{10Ty1}$
Anti-TY1
