# Supplementary material for: Novel axonemal protein ZMYND12 interacts with TTC29 and DNAH1, and is required for male fertility and flagellum function
Source: eLife. 2023 Nov 7;12:RP87698. doi: 10.7554/eLife.87698 (PMC10629824; doi:10.7554/eLife.87698)
Supplement: Supplementary file 1. [file elife-87698-supp1.docx]

**Supplementary File 1.** Proteins co-immunoprecipitated with TbTAX-1 and TbTTC29 identified by mass spectrometry analyses.

| **TbTAX-1_HA_ immunoprecipitation** | | |
| --- | --- | --- |
| **Accession**  **number** | **Protein** | **Fold-enrichment** |
| Tb927.9.10370 | TbTAX-1 (Broadhead et al., 2006) | 20.6 |
| Tb927.3.1990 | TbTTC29 (Lorès et al., 2019) | 20.3 |
| Tb927.11.8160 | Dynein Heavy Chain putative.  DNAH1 ortholog putative (Dean et al., 2016) | 6.66 |
| Tb927.10.15350 | Histone H3 variant V | 6.25 |
| Tb927.10.14680 | Ribosome biogenesis, putative | 5.88 |
| Tb927.11.3880 | TrypARP,  Actin putative(Ersfeld and Gull, 2001) | 5.7 |
| Tb927.11.3830 | Hypothetical protein, conserved | 4.42 |
| Tb927.10.8940 | Flagellum targeting protein kharon1 | 4.24 |
| Tb927.11.970 | Hypothetical protein, conserved | 4.1 |
| Tb927.10.170 | Pseudouridine synthase, Cbf5p | 3.7 |
| Tb927.9.12610 | Glycerol kinase, glycosomal | 3.7 |
| Tb927.2.2950 | Nop14-like family, putative | 3.5 |
| Tb11.v5.0732.1 | Hexokinase, putative | 3.1 |
| Tb927.11.16720 | Mitotic cyclin 6, CYC6 | 2.4 |
| Tb927.11.8870 | DEAD-box ATP-dependent RNA helicase, MHEL61 | 2.3 |

| **TbTTC29_TY1_ immunoprecipitation** | | |
| --- | --- | --- |
| **Accession**  **number** | **Protein** | **Fold-enrichment** |
| Tb927.3.1990 | TbTTC29 (Lorès et al., 2019) | 27.3 |
| Tb927.11.8160 | Dynein Heavy Chain putative.  DNAH1 ortholog putative (Dean et al., 2016) | 9.2 |
| Tb927.9.10370 | TbTAX-1 (Broadhead et al., 2006) | 8.6 |
| Tb927.11.11330 | HSP70 | 6. 5 |
| Tb927.11.3880 | TrypARP,  Actin putative(Ersfeld and Gull, 2001) | 5.88 |
| Tb927.3.1790 | Pyruvate dehydrogenase E1 beta subunit, putative | 2.4 |
