# Supplementary material for: Novel axonemal protein ZMYND12 interacts with TTC29 and DNAH1, and is required for male fertility and flagellum function
Source: eLife. 2023 Nov 7;12:RP87698. doi: 10.7554/eLife.87698 (PMC10629824; doi:10.7554/eLife.87698)
Supplement: Supplementary file 3. [file elife-87698-supp3.docx]

**Supplementary File 3.** Primer sequences for Sanger sequencing verification of *ZMYND12* variants.

| **Primers** | **Primer sequence (5’-3’)** | **Tm (°C)** | **Product length (bp)** |
| --- | --- | --- | --- |
| *ZMYND12*-F-Ex4 | AGGCTGCTGTGAACATTCCT | 57.3 | 250 |
| *ZMYND12*-R-Ex4 | AATGCTTGAACTCGGGTCGG | 59.4 |  |
