# Supplementary material for: Novel axonemal protein ZMYND12 interacts with TTC29 and DNAH1, and is required for male fertility and flagellum function
Source: eLife. 2023 Nov 7;12:RP87698. doi: 10.7554/eLife.87698 (PMC10629824; doi:10.7554/eLife.87698)
Supplement: Supplementary file 4. [file elife-87698-supp4.docx]

**Supplementary File 4**. *ZMYND12* MLPA probes used in this work.

| **MLPA probes** | **Upstream hybridizing sequence (5’-3’)** | **Downstream hybridizing sequence (5’-3’)** | **Total probe length (nt)** |
| --- | --- | --- | --- |
| ***ZMYND12* specific probes** | | | |
| exons 5 | GCTACTTCCACCTGGCTAATATATTCTATGACCTT | AAAAAGTTGGACCTGGCAGACACATTGTACACCAA | 112 |
| exon 6 | GGTCTCTGAGATCTGGCATGCATATTTGAAC | AATCACTATCAAGTCCTCTCACAGGCTCACATCCA | 108 |
| exon 8 | CTCAGTCTAGCCAAAGAACAACAGCTTGA | TGTCCATGAGCAAAGCACCATTCAAGAGT | 98 |

For each probe, the unique hybridizing sequence of the two half-probes is given. Total probe length represents the total length of amplification product (nt=nucleotides) = forward primers + upstream hybridizing sequence + downstream hybridizing sequence + reverse primers.
