# Supplementary material for: Novel axonemal protein ZMYND12 interacts with TTC29 and DNAH1, and is required for male fertility and flagellum function
Source: eLife. 2023 Nov 7;12:RP87698. doi: 10.7554/eLife.87698 (PMC10629824; doi:10.7554/eLife.87698)
Supplement: Supplementary file 5. [file elife-87698-supp5.docx]

**Supplementary File 5.** Primers used for RT-qPCR detection of *ZMYND12* in human tissue extracts.

| **Primer names** | **Primer sequences (5’-3’)** | **Tm** |
| --- | --- | --- |
| ZMYND12_RT_F | TACTGCATCGGAATCTGGGAC | 59 °C |
| ZMYND12_RT_R | GCCAGATGATAACGGGCCTC |  |
| RPL6_RTqPCR_F | TCCATTCGTCAGAGCAAACA | 56 °C |
| RPL6_RTqPCR_R | TACGGAGCAGCGCAAGAT |  |
| RPL27_RTqPCR_F | AGAGTACCTTGTGGGCAT | 56 °C |
| RPL27_RTqPCR_R | TGATGGCACCTCAGATCGC |  |

Tm: melting temperature
