# Supplementary material for: Novel axonemal protein ZMYND12 interacts with TTC29 and DNAH1, and is required for male fertility and flagellum function
Source: eLife. 2023 Nov 7;12:RP87698. doi: 10.7554/eLife.87698 (PMC10629824; doi:10.7554/eLife.87698)
Supplement: Supplementary file 6. [file elife-87698-supp6.docx]

**Supplementary File 6.** Primary antibodies used in immunofluorescence experiments with human samples

| Primary antibodies | Reference | Species | Localization | Dilution |
| --- | --- | --- | --- | --- |
| Human experiments | | | | |
| AKAP4 | Sigma-Aldrich® HPA020046 | Rabbit | Fibrous sheath | 1/100 |
| CFAP70 | Sigma-Aldrich® HPA062453 | Rabbit | Unknown (base of ODA?) | 1/50 |
| DNAH1 | Sigma-Aldrich® HPA036806 | Rabbit | IDA | 1/25 |
| DNAH17 | Sigma-Aldrich® HPA024354 | Rabbit | ODA | 1/200 |
| DNAH8 | Sigma-Aldrich® HPA028447 | Rabbit | ODA | 1/200 |
| DNAI1 | Sigma-Aldrich® HPA021649 | Rabbit | ODA | 1/200 |
| DNALI1 | Sigma-Aldrich® HPA028305 | Rabbit | IDA | 1/100 |
| GAS8 | Sigma-Aldrich® HPA041311 | Rabbit | N-DRC | 1/100 |
| RSPH1 | Sigma-Aldrich® HPA017382 | Rabbit | RS | 1/100 |
| SPAG6 | Sigma-Aldrich® HPA020046 | Rabbit | CPC | 1/500 |
| TTC29 | Sigma-Aldrich® HPA061473 | Rabbit | Unknown  (IDA? IFT?) | 1/100 |
| WDR66 | Sigma-Aldrich® HPA040005 | Rabbit | CSC | 1/20 |
| ZMYND12(PA5-69715) | Sigma-Aldrich® HPA062453 | Rabbit | Unknown | 1/100 |
| β-tubulin | Cell signal 2128 | Rabbit | microtubules | 1/500 |
| β-tubulin | Sigma-Aldrich® T4026 | Mouse | microtubules | 1/500 |

Abbreviations: ODA, Outer Dynein Arms; IDA, Inner Dynein Arms; RS, Radial Spokes; FS, Fibrous sheath; CPC, Central Pair Complex; N-DRC: Nexin-Dynein Regulatory Complex, CSC: calmodulin-associated and spoke-associated complex
