# Supplementary material for: Novel axonemal protein ZMYND12 interacts with TTC29 and DNAH1, and is required for male fertility and flagellum function
Source: eLife. 2023 Nov 7;12:RP87698. doi: 10.7554/eLife.87698 (PMC10629824; doi:10.7554/eLife.87698)
Supplement: Supplementary file 7. [file elife-87698-supp7.docx]

**Supplementary File 7.** Primary antibodies used in immunofluorescence experiments with *Trypanosoma* cells

| **Antibody** | **Provider & Reference** | **Dilution IF** | **Dilution U-ExM** | **WB Dilution** | **IP Dilution** |
| --- | --- | --- | --- | --- | --- |
| **Primary antibodies** |  |  |  |  |  |
| Anti-PFR2 | (Coutton et al., 2018) | 1:500 | - | - | - |
| Anti-TbSAXO | (Dacheux et al., 2012) | 1:1000 | - | 1:1000 |  |
| Anti-Myc | Mouse monoclonal 9E10 | 1:1000 | - | - | - |
| Anti-Myc | Sigma c-3956 | - | - | 1:1000 | - |
| Anti-Ty1 (purified supernatant) | (Brookman et al., 1995) | 1:2000 | 1:500 | 1:10,000 | 1:33 |
| Anti-HA | Genetex GTX628902 | - | - | - | 1:50 |
| Anti-Enolase | (Hannaert et al., 2003) | - | - | 1:25,000 | - |
| Anti-Tubulin | (Woods et al., 1989) | - | - | 1:1000 | - |
| **Secondary antibodies** |  |  |  |  |  |
| Anti-mouse FITC | Sigma F2012 | 1:100 | - | - | - |
| Anti-rabbit Alexa fluor 594 | Molecular Probes A21201 | 1:200 | - | - | - |
| Anti-rabbit Alexa fluor A647 | Molecular Probes A31573 | - | 1:500 | - | - |
| Anti-mouse Alexa fluor A488 | Molecular Probes A11001 | - | 1:500 | - | - |
| Anti-mouse HRP | Jackson 115-035-062 | - | - | 1:10,000 | - |
| Anti-rabbit HRP | Sigma A-9169 | - | - | 1:10,000 | - |
| Anti-rabbit StarBright Blue 700 | Bio-Rad 12004162 | - | - | 1:2500 | - |
| Anti-mouse StarBright Blue 520 | Bio-Rad 12005867 | - | - | 1:2500 | - |
